# Comparative analysis of the plastid and mitochondrial genomes of *Artemisia giraldii* Pamp.

**DOI:** 10.1038/s41598-022-18387-2

**Published:** 2022-08-17

**Authors:** Jingwen Yue, Qianqi Lu, Yang Ni, Pinghua Chen, Chang Liu

**Affiliations:** 1 Key Laboratory of Ministry of Education for Genetics, Breeding and Multiple Utilization of Crops, National Engineering Research Center of Sugarcane, College of Agriculture, Fujian Agriculture and Forestry University, No.15, Shangxiadian Road, Fuzhou, 350002 Fujian People’s Republic of China; 2 Institute of Medicinal Plant Development, Chinese Academy of Medical Sciences, Peking Union Medical College, No. 151, Malianwa North Road, Haidian District, Beijing, 100193 People’s Republic of China

**Keywords:** Genomic analysis, Comparative genomics

## Abstract

*Artemisia giraldii* Pamp. is an herbaceous plant distributed only in some areas in China. To understand the evolutionary relationship between plastid and mitochondria in *A. giraldii*, we sequenced and analysed the plastome and mitogenome of *A. giraldii* on the basis of Illumina and Nanopore DNA sequencing data. The mitogenome was 194,298 bp long, and the plastome was 151,072 bp long. The mitogenome encoded 56 genes, and the overall GC content was 45.66%. Phylogenetic analysis of the two organelle genomes revealed that *A. giraldii* is located in the same branching position. We found 13 pairs of homologous sequences between the plastome and mitogenome, and only one of them might have transferred from the plastid to the mitochondria. Gene selection pressure analysis in the mitogenome showed that *ccm*Fc, *nad*1, *nad*6, *atp*9, *atp*1 and *rps*12 may undergo positive selection. According to the 18 available plastome sequences, we found 17 variant sites in two hypervariable regions that can be used in completely distinguishing 18 *Artemisia* species. The most interesting discovery was that the mitogenome of *A. giraldii* was only 43,226 bp larger than the plastome. To the best of our knowledge, this study represented one of the smallest differences between all sequenced mitogenomes and plastomes from vascular plants. The above results can provide a reference for future taxonomic and molecular evolution studies of Asteraceae species.

## Introduction

*Artemisia* is one of the largest and most widely distributed genera in the family of Asteraceae. It is a heterogeneous genus consisting of more than 500 different species distributed mainly in Europe, Asia and North America^1,2^. These species are perennial, biennial and annual herbs or small shrubs^3,4^. Its pungent odor and bitter taste are due to terpenoids and sesquiterpene lactones^5^. Some *Artemisia* species are cultivated as crops, whereas others are used in preparing tea, tonic, alcoholic beverages and medicines^6^. Various biochemically active secondary metabolites have been identified in *Artemisia* species, including essential oils, flavonoids, terpenoids, esters and other substances^4,7–9^, which are potential bioactive compounds for developing novel herbal drugs against multiple diseases, such as cancer^10^, malaria^11^, hepatitis, inflammation^12^ and fungal, bacterial^13^ and viral infections^14^. Researchers have extracted artemisinin from *Artemisia annua* and demonstrated its antimalarial effects^15^. Tu et al. converted artemisinin into a drug that has saved millions of lives worldwide^16^, thus winning the 2015 Nobel Prize in medicine. These researchers have confirmed the medicinal value of *Artemisia* species and its potential use in bio-exploration.

*Artemisia giraldii* Pamp. is one of the 186 *Artemisia* species found in China. It is an herbaceous plant distributed only in some areas of China (e.g., Henan, Hebei, Gansu, Ningxia, Shannxi and Sichuan Provinces). Studies on *A. giraldii* are few and have mainly focus on its chemical composition, geographical distribution^17^ and community^18^. The main chemicals in *Artemisia* are terpenoids, flavonoids, coumarins, caffeoylquinic acids, sterols and acetylenes. Two flavones and several monoterpenoids and sesquiterpenoids have been isolated from the aerial parts of *A. giraldii*^7,8^. These two flavones named 4′,6,7-trihydroxy-3′,5′-dimethoxyflavone and 5′,5-dihydroxy-3′,4′,8-trimethoxyflavone showed antibiotic activity against *Escherichia coli*, *Sarcina lutea*, *Pseudomonas aeruginosa* and *Aspergillus flavus*^8^. A monoterpene, called santolinylol, which has antifungal activity, has been isolated from *A. giraldii*^19,20^. The flowering parts of *A. giraldii* are rich in essential oils. Studies have shown that these essential oils exhibit strong fumigant activity against *Sitophilus zeamais* adults and possessed substantial contact toxicity against maize weevils^21^.

Molecular breeding, genetic engineering and synthetic biology of *Artemisia* species have attracted considerable interest, which are critical to obtaining active materials efficiently. The first steps for genetic studies include sequencing and analysing the nuclear and organelle genomes.

Mitochondria and plastids originate from bacterial endosymbionts^22^. The convergent evolution of mitochondria and plastid can be observed between distantly related species, the same strain and even within the same cell. However, although mitochondrial and plastid genomes follow similar evolutionary paths, mitochondrial genomes have evolved much further^23^. The mitochondrial genome (mitogenome) is more complex than the plastid genome and more severe gene loss, more extensive and refined forms of post-transcriptional editing and processing, more gene isoforms and a wider range of gene fragmentation in most photosynthetic plants^24,25^. However, the number of plastid genes is not larger than that of mitochondrial genes in some plants. In some non-photosynthetic plants, such as *Hypopitys monotropa*^26^ or *Rhopalocnemis phalloides*^27^, the plastomes showed considerable gene loss and size reduction. The plastome size decreases up to 110–200 kb in autotrophic plants^28^. Co-extension/coexistence of mitochondrial and plastid genomes was observed in various species, and in most cases, plastid DNA was overtaken by mitochondrial DNA^29^. We can identify the interaction between the two organelles from the comparative analysis of mitochondrial and chloroplast genomes of the same species.

The animal mitogenome is normally a circular, compact molecule about 17 kb long with little variation in size. It contains about 13 protein-coding genes (PCGs), two ribosomal RNAs (rRNA) genes and 22 or 23 transfer RNA (tRNA) genes among bilaterians, with a few exceptions^30^. Although much larger mitochondrial genomes have occasionally been found, they are usually the product of duplicating portions of the mtDNA rather than variation in gene content^31^. Unlike the relatively simple animal mitochondrial genomes, non-parasitic flowering plant mitochondrial genomes were large and complex^32–34^. They exhibit a wide range of variations in size, sequence alignment and repeat content, but the coding sequences are highly conserved (typically 24 core genes and 17 variable genes)^35,36^. Usually, the mitogenome was represented as a monomeric circle with no mention of other forms^37,38^, as circular mapping is a convenient indicator of genome content and sequencing completion. Thus, the circular map appears in published plant mitochondrial genomes^39^. However, plant mitochondrial DNAs appear as linear and multi-branched molecules under electrophoresis and microscopy. At the same time, some studies have also proposed that plant mitochondria are non-circular. They are a collection of multiple forms, including circular, linear and branching molecules. Some of these molecules might represent the intermediate molecule of replication or recombination^40^. Multiple forms can also be called isomers of the genome. The cause of isomers may be the frequent recombination of some repetitive sequences in the plant mitochondrial genome promoting rearrangement of the genome^41,42^, which is also indirectly indicated by the near-complete disruption of gene order among closely related species^43,44^. Cytoplasmic male sterility (CMS) is the most evident and widespread phenotype associated with plant mitogenomic rearrangements (CMS). CMS has long been of interest to plant breeders because the male-sterile phenotype contributes to hybrid seed production. Mining whole mitogenome sequences can complement the experimental approaches^39,45^. In particular, they can reveal the origin, expression and evolution of CMS genes and the effect of CMS on mitogenome evolution.

Seven thousand three hundred sixty-three complete plastomes and 423 plant mitogenomes have been recorded in the GenBank Organelle Genome database (https://www.ncbi.nlm.nih.gov/genome/browse/) (last updated: December 20, 2021). The structural complexity of mitochondria results in significantly more difficulty in their genome assembly. Only a few mitochondria mitogenomes have been reported. Until now, no mitogenome in the *Artemisia* genus has been reported. This deficit has limited our understanding of the evolution and functioning of the mitochondria in this genus. Here, we assembled and annotated the plastome and mitogenome of *A. giraldii* for the first time. We analysed the gene content, repeat sequence and selection pressure of the *A. giraldii* mitogenome. In addition to these, we attempted to understand the evolving relationship between the plastomes and mitogenomes of Asteraceae species by constructing phylogenetic trees of 10 Asteraceae species. Lastly, we analysed the homologous sequence between the two organelle genomes. The results obtained from this study provide the first account of the mitogenome structure and shed light on the interaction between the mitogenome and plastome.

## Materials and methods

### Plant materials and DNA extraction and sequencing

We collected fresh *A. giraldii* Pamp. Leaves from the Institute of Medicinal Plant Development (IMPLAD), Beijing, China. Then, the total genomic DNA (accession number: implad201910017) was extracted using a DNA extraction Kit (Tiangen Biotech, Beijing, China) and stored in a refrigerator at − 80 °C. A DNA sequence library was constructed with 1 ug of DNA by using a NEBNext library building kit and sequenced with a 2500 platform (Illumina, San Diego, CA, USA). Clean data were obtained by removing low-quality sequences with Trimmomatic software^46^ under the following conditions: sequences with more than 50% bases with quality values (Q) of < 19 and more than 5% ‘N’ bases. The plant sample used for Illumina short‐read sequencing was subsequently used for Oxford Nanopore sequencing. Raw reads obtained by Nanopore sequencing were filtered to remove reads with Q of < 7. Genomic DNA was prepared using the CTAB method and purified with a QIAGEN genomic kit (Cat# 13343, QIAGEN) according to the standard operating procedure provided by the manufacturer. About 700 ng of DNA was used in library construction and then sequenced on a Nanopore PromethION sequencer instrument (Oxford Nanopore Technologies, UK) at the Genome Center of Grandomics (Wuhan, China).

### Genome assembly and annotation

GetOrganelle^47^ was used in assembling the organelle genomes. We first used the Illumina data alone to assemble the plastome. The parameters applied for plastome were ‘-R 15 -k 21,45,65,85,105 -F embplant_pt’. Then, we applied a hybrid strategy combining Illumina and Nanopore reads to assemble the mitogenome. GetOrganelle was used in extracting mitochondrial genome reads from Illumina whole-genome sequence (WGS) data. We then assembled the extracted reads into a unitig graph. All the ‘edges’ of the unitig graph had the same coverage depth, suggesting the absence of plastid and nuclear sequences, which tend to show significantly higher or lower coverage depths. The unitig graph contained multiple double-bifurcation structures (‘>  =  <’, DBSs) resulting from the presence of repeat sequences in the genome. To resolve the sequence path around these DBS, we constructed all possible sequences around the DBSs and mapped them to the Nanopore reads with minimap2 tool^48^. For each DBS, we selected the sequence path with the largest number of Nanopore reads mapped as the dominant sequence path. Finally, we identified a cyclic path on the unitig graph covering all the ‘edges’. This path corresponded to a circular DNA sequence, which was considered the mitogenome.

The plastome was annotated using CPGAVAS2^49^, and the reference genome was *Chrysanthemum indicum* (NC_020320.1)^50^. The diagrams of cis-splicing and trans-splicing genes in the plastome were created using CPGview-RSG (http://www.herbalgenomics.org/cpgview). The mitogenome was annotated using MGAVAS (http://www.1kmpg.cn/mgavas) and GeSeq (https://chlorobox.mpimp-golm.mpg.de/geseq.html)^51^, and its reference genome was *C. indicum* (MH716014.1)^52^. We annotated the mitogenome using MGAVAS (http://www.1kmpg.cn/mgavas/) and tRNAscan-SE^53^ with default settings to confirm the annotations. We used Apollo^54^ to manually correct the annotation problems and OrganellarGenomeDRAW (OGDRAW) (v1.3.1)^55^ to draw a genome map. Then, we submitted the organelle genome sequences and annotations to GenBank by BankIt (https://www.ncbi.nlm.nih.gov/WebSub/) and obtained accession numbers OK128342 for the plastome and NC_064134.1 for the mitogenome.

### Homology sequence analysis between plastid and mitochondrion

Sequence similarity comparison between the plastome (OK128342) and mitogenome (NC_064134.1) was carried out for the identification of homologous sequences between two organelles. BLASTN was used, and the e-value cutoff was 1*e*–5^56^. The final results were visualised using the Circos package implemented in TBtools^57,58^.

### Repeat elements analysis

The microsatellite sequence repeats were identified by using Misa (https://webblast.ipk-gatersleben.de/misa/) with the parameters ‘1-10 2-5 3-4 4-3 5-3 6-3’^59^. The tandem repeats were identified using TRF with the following parameters: ‘2 7 7 80 10 50 500 -f -d -m’^60^. The dispersed repeats were identified using REPuter web server (https://bibiserv.cebitec.uni-bielefeld.de/reputer/) with the following parameters: hamming distance, 3; maximum computed repeats, 5000; and minimal repeat size, 30 and filtered at an e-value of 1*e*−4^61^. Visualisation was conducted according to the procedure for homologous sequence analysis.

### Phylogenetic inference analysis

The plastome and mitogenomes of *A. giraldii* combined with 11 Asteraceae species were used in phylogenetic analysis. Two *Solanum* genus species were selected as outgroup taxa. The common genes of 12 species were extracted using Phylosuite (v1.1.16)^62^. From the plastome, we extracted the coding sequences from 67 common genes (*atp*A, *atp*B, *atp*E, *atp*F, *atp*H, *ccs*A, *cem*A, *mat*K, *ndh*A, *ndh*B, *ndh*C, *ndh*D, *ndh*E, *ndh*F, n*dh*G, *ndh*H, *ndh*I, *ndh*J, *ndh*K, *pet*A, *pet*B, *pet*D, *pet*G, *pet*L, *pet*N, *psa*A, *psa*B, *psa*C, *psa*I, *psa*J, *psb*A, *psb*D, *psb*E, *psb*F, *psb*H, *psb*I, *psb*J, *psb*K, *psb*M, *psb*N, *psb*T, *rbc*L, *rpl*2, *rpl*14, *rpl*16, *rpl*20, *rpl*22, *rpl*32, *rpl*33, *rpl*36, *rpo*A, *rpo*B, *rpo*C1, *rpo*C2, *rps*2, *rps*3, *rps*4, *rps*7, *rps*8, *rps*11, *rps*12, *rps*15, *rps*16, *rps*18, *rps*19, *ycf*3 and *ycf*4) from 10 Asteraceae species and two outgroup taxa for phylogenetic analysis. From the mitogenome, we extracted 29 orthologous mitochondrial genes (*atp*1, *atp*4, *atp*6, *atp*8, *atp*9, *cox*1, *cox*2, *cox*3, *ccm*B, *ccm*C, *ccm*Fc, *ccm*Fn, *cyt*b, matR, *mtt*B, *nad*1, *nad*2, *nad*3, *nad*4, *nad*4L, *nad*5, *nad*6, *nad*7, *nad*9, *rpl*10, *rps*3, *rps*4, *rps*12 and *rps*13) from the same set of species for analysis. Then, we aligned the coding sequences with MAFFT (v7)^63^ and concatenated them with Phylosuite (v1.1.16). We used Gblocks with default parameters to optimise the alignment of the concatenated sequences^64^. The phylogenetic tree was built using the maximum-likelihood method implemented in IQ-TREE (v2)^65^ and visualised using iTOL (v5; https://itol.embl.de/)^66^. Bootstrap analysis was performed using UFBoot with 1000 replicates^65^. The best model was selected using jModelTest (v2.1.0)^67^ according to Bayesian information criterion. TVM + G was found to be the best model for plastome and mitogenome analyses. We performed Bayesian inference (BI) analysis using MrBayes (v3.2.7)^68^. The BI tree was visualised using iTOL (v5)^66^.

### Selective pressure analysis of *A. giraldii* mitogenome

We used EasyCodeML (v1.4) software^69^ to conduct the selective pressure analysis of 28 protein-coding genes in the mitogenome. The running model was ‘Preset (Nested Models)’. The site model in EasyCodeML can be used in identifying positively selected sites in a multiple-sequence alignment^70^. The required inputs for analysing selection are aligned sequences in PAML format and a tree file in Newick format. Firstly, we aligned each gene from 10 species with MAFFT (v7)^63^ and converted the alignment into PAML format by using the ‘Seqformat Convertor’ tool in EasyCodeML (v1.4). Then, we used IQ-TREE (v2)^65^ to generate a tree file in Newick format. Finally, we ran the CodeML with the following parameters: nt = 0 and icode = 0’. On the basis of the lnL and np values of the null model (M0, M1a, M7 and M8a) and alternative model (M3, M2a and M8), the likelihood ratio test (LRT) p-value of each PCG was calculated. Then, the p-values were adjusted using the Benjamini–Hochberg correction method^71^. Genes with adjusted p-values of < 0.05 were considered positively selected.

### Molecular marker development

To discover universal primers that can be used in distinguishing the *Artemisia* species, we downloaded the 17 plastome sequences of *Artemisia* species from GenBank. They were analysed using ecoPrimers^72^ with the following parameters: ‘-l 300 -L 600 -e 0 -3 2 -t species -U -f -O 25’. Here, ‘-l 300’ specified the minimum barcode length as 300, excluding primers. ‘-L 600’ specified the maximum barcode length as 600, excluding primers. ‘-e 0’ specified the maximum number of mismatches allowed per primer as 0. ‘-3 2’ specified the number of nucleotides on the 3′ end of the primers as 2, and these primers should have a strict match with their target sequences. ‘-t species’ specified the taxonomic level used for evaluating barcodes and primers as ‘species’. ‘-U’ meant that no multi match of a primer on the same sequence record is allowed. ‘-f’ indicated the removal of data mining step during strict primer identification. ‘-O 25’ specified the primer length to be 25. A custom script was used to extract the regions adjacent to the identified DNA barcode region for designing PCR primers.

### Hypervariable region analysis

To identify the hypervariable regions among the 18 *Artemisia* species, we wrote a custom script to extract the intergenic spacer regions (IGS) from the GenBank files of the 18 plastomes. Firstly, we extracted the IGS sequences using extractseq. Then, we aligned the extracted sequences using clustalw2^73^ with options ‘-type = DNA -gapopen = 10 -gapext = 2’. Finally, we calculated the genetic distance of the intergenic regions using the K2p evolution model implemented in the distmat program from the EMBOSS package^74^ with the parameter ‘-nucmethod 2’. Fourteen hypervariable IGS were identified (Fig. 6). To verify whether these molecular markers can distinguish the 18 *Artemisia* species, we extracted the top three most variable IGS regions from 18 *Artemisia* species for the alignment.

### Ethics approval and consent to participate

We collected fresh leaf materials from *A. giraldii* for this study. No specific permits were required from the local government for the collection. In addition, we conducted the study in compliance with relevant institutional, national and international guidelines and legislation. We prepared the voucher specimens and deposited them in the Institute of Medicinal Plant Development (Beijing, China) with the accession number implad201910017.

## Results

### DNA sequencing, genome assembly and validation

In the Illumina sequencing data, a total of 21,579,647 sequences was generated, and the total number of bases was 3,236,947,050. The average read length was 150 bp. In the Nanopore sequencing data, a total of 10.225 Gb of 1,800,259 reads were obtained, and 8.227 Gb of 1,389,001 reads had Q of > 7, which were used in subsequent analysis. The average length of the remaining reads was 5.923 kb, N50 was 14.074 kb and the longest read was 114.470 kb. We used two strategies to assemble the plastome. In the first strategy, we used Illumina data alone. In the second strategy, we used the Illumina and Nanopore reads. The assembled results were identical except that the small single-copy (SSC) region was inverted between the two assemblies (Supplementary Fig. S1A). In the mitogenome assembly, we used Illumina and Nanopore reads.

We mapped the Illumina reads to the assembly results to obtain the coverage depth of the plastome and examine the quality of the assembly (Supplementary Fig. S2). To determine the coverage depth of the mitogenome, we mapped the Illumina reads to the hybrid assembly results (Supplementary Fig. S3). The average coverage depth was 121× for the mitogenome and 430× for the plastome. For locations with low coverage depths in the mitogenome and plastome, we used Tablet software^75^ to visualise read cover in the genome. All low-coverage locations had spanned reads (Supplementary Figs. S2 and S3). We found more than 30 reads that covered the plastome locations with low coverage depths. By contrast, we found more than 10 reads that covered the mitogenome locations with low coverage depths. We used Bandage^76^ to visualise the structure of the *A. giraldii* plastome (Supplementary Fig. S4A) and mitogenome (Supplementary Fig. S4B). The plastome was a typical circular sequence containing a large single-copy (LSC) region, a pair of identical inverted repeats (IRs) and an SSC region (Supplementary Fig. S4A).

The unitig graph of the mitogenome showed a branched polymeric structure (Supplementary Fig. S4B). Different contigs (Supplementary Fig. S4B, left side) were linked to form a master chromosome (Supplementary Fig. S4B, right side). The principle chromosome can undergo rearrangement through repeat-mediated recombination, generating chromosomes with different rearrangements, called isomers^40^. We manually removed non-mitochondrial nodes from the graph according to the stratified coverage depth, and the repeat paths were resolved by aligning with the Nanopore long reads. Finally, a circular mitochondrial molecule was obtained (Supplementary Fig. S4). The master chromosome encoded 54 genes: 32 PCGs, 3 rRNAs and 21 tRNAs. The quantities were consistent with those found in other Asteraceae species.

### General features of the* A. giraldii* organelle genomes

**Figure 1:**
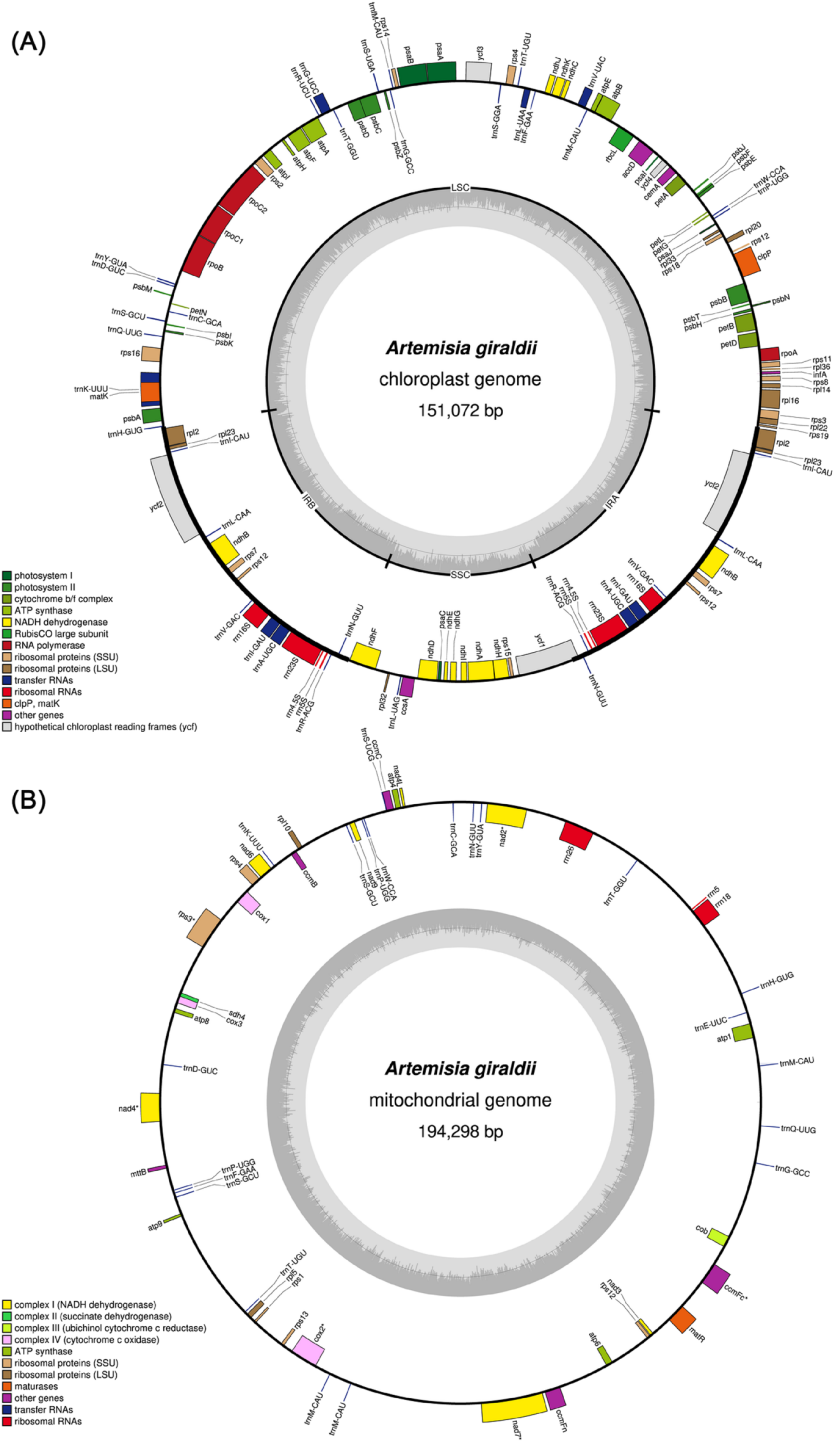
The circular maps of the organelle genomes of *A. giraldii*. (**A**) The circular map of the plastome. (**B**) The circular map of the mitogenome. The functions of the different colored genes on the map are shown on the left. The dark gray region in the inner circle indicates the GC content. The circular maps of two organelle genomes were drawn by Geseq (https://chlorobox.mpimp-golm.mpg.de/geseq.html).

To understand the characteristics of the mitogenome and plastome of *A. giraldii*, we analysed their general features. The entire length of the plastome was 151,072 bp, and it was divided into four regions: an LSC region of 82,838 bp, an SSC region of 18,316 bp and a pair of identical 24,959 bp IRs (Fig. 1A). A total of 109 unique genes were found in the *A. giraldii* plastome: 78 PCGs, 27 tRNA genes, and 4 rRNA genes (Supplementary Table S1). Among these genes, 19 genes (*rpl*16, *pet*D, *pet*B, *trn*V-UAC, *trn*L-UAA, *trn*G-UCC, *atp*F, *rpo*C1, *rps*16, *trn*K-UUU and *rpl*2) had one intron, and two genes (*clp*P, *ycf*3) had two introns (Supplementary Table S2). Eleven cis-splicing genes (*rpl*16, *pet*D, *pet*B, *clp*P, *ycf*3, *atp*F, *rpo*C1, *rps*16, *rpl*2, *ndh*B and *ndh*A) were found in the *A. giraldii* plastome (Supplementary Fig. S5), and all these genes were PCGs. The cis-splicing genes *rpl*2 and *ndh*B had two introns. *rps*12 was the only trans-splicing gene identified (Supplementary Fig. S6).

The total length of PCGs in *A. giraldii* plastome was 78,009 bp, representing 51.64% of the whole length of the plastome sequence. By contrast, the size of the rRNA was 9046 bp, and the size of the tRNA was 2693 bp, representing 5.99% and 1.78% of the total length of the *A. giraldii* plastome sequence, respectively. The GC content analysis showed that the overall GC content was 37.47%. In particular, the GC content for the protein-coding regions, rRNA genes and tRNA genes was 37.78%, 55.10% and 52.73%, respectively. The GC content in the LSC, SSC and IR regions was 35.56%, 30.78% and 43.09%, respectively.

The total length of the *A. giraldii* mitogenome was 194,298 bp. The base composition of the entire mitogenome was A (27.26%), G (22.75%), T (27.08%) and C (22.90%). The entire GC content was 45.66%. We annotated 32 PCGs in the mitogenome (Fig. 1B). According to these functions, these 32 genes can be divided into 10 classes: ATP synthase (*atp*1, *atp*4, *atp*6, *atp*8 and *atp*9), cytochrome (*ccm*B, *ccm*C, *ccm*Fc and *ccm*Fn), ubichinol cytochrome c reductase (*Cob*), cytochrome c oxidase (*cox*1, *cox*2 and *cox*3), maturases (*mat*R), transport membrane protein (*mtt*B), NADH dehydrogenase (*nad*1, *nad*2, *nad*3, *nad*4, *nad*4L, *nad*5, *nad*6, *nad*7 and *nad*9), large subunit of ribosome (*rpl*5, *rpl*10), small submit of ribosome (*rps*1, *rps*3, *rps*4, *rps*12 and *rps*13) and succinate dehydrogenase (*sdh*4; Table 1).

**Table 1:** Gene composition in the *A. giraldii* mitogenome.

Group of genes	Name of genes
ATP synthase	*atp*1, *atp*4, *atp*6, *atp*8, *atp*9
Cytochrome c biogenesis	*ccm*B, *ccm*C, *ccm*Fc*, *ccm*Fn
Ubiquinol cytochrome c reductase	*Cob*
Cytochrome c oxidase	*cox*1, *cox*2*, *cox*3
Maturases	*mat*R
Transport membrane protein	*mtt*B
NADH dehydrogenase	*nad*1*, *nad*2*, *nad*3, *nad*4*, *nad*4L, *nad*5*, *nad*6, *nad*7*, *nad*9
Large subunit of ribosome	*rpl*5, *rpl*10
Small subunit of ribosome	*rps*1, *rps*3*, *rps*4, *rps*12, *rps*13
Succinate dehydrogenase	*Sdh*4
Ribosomal RNAs	*rrn*5, *rrn*18, *rrn*26
Transfer RNAs	*trn*Y-GUA, *trn*W-CCA, *trn*S-GCU (×2), *trn*P-UGG, *trn*N-GUU, *trn*M-CAU (×3), *trn*K-UUU, *trn*H-GUG, *trn*G-GCC, *trn*F-GAA, *trn*E-UUC, *trn*D-GUC, *trn*C-GCA, *trn*T-GGU, *trn*T-UGU, *trn*P-UGG (×2), *trn*Q-UUG

‘*’ genes that contain introns.

### Comparison of genomic features with the other nine Asteraceae mitogenomes

**Figure 2:**
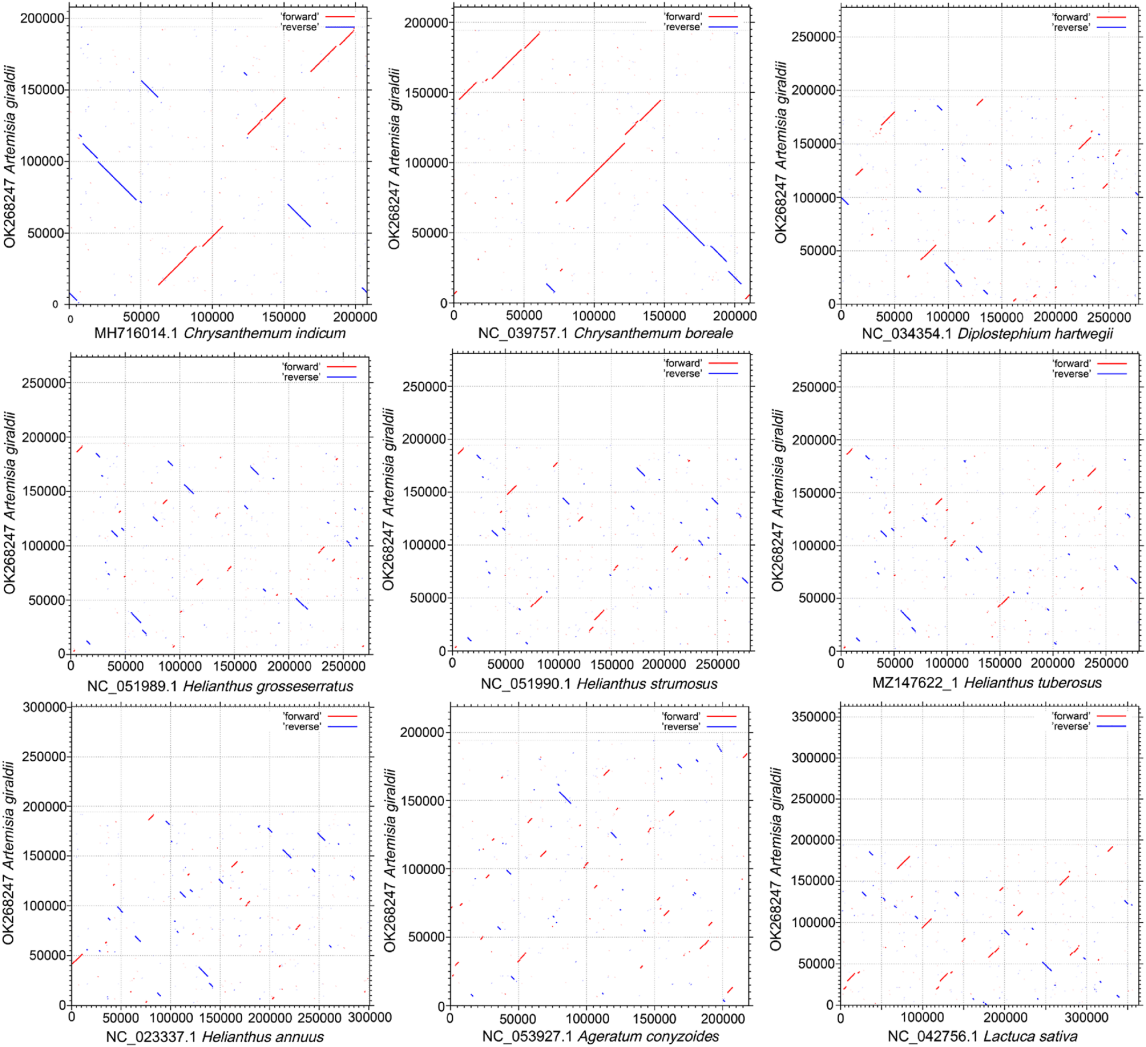
The dotplot graphs of collinearity between the mitogenomes of the *A. giraldii* and nine Asteraceae species. The vertical axis represents the *A. giraldii* mitogenome. The horizontal axis represents the nine Asteraceae mitogenomes, respectively. The red and blue lines showed the homologous regions in the forward and reverse direction between the *A. giraldii* and nine Asteraceae species, respectively. These dotplot graphs were drawn by MAFFT online service (https://mafft.cbrc.jp/alignment/server/).

Angiosperm mitogenomes vary greatly in genome structure, gene content and constitution. Variations in mitogenome size can be explained mostly by difference in length among intergenic regions^25^. We compared the length, GC content and PCG number of *A. giraldii* with the mitogenomes from nine other published Asteraceae species: *Lactuca sativa*, *Diplostephium hartwegii*, *Chrysanthemum boreale*, *C indicum*, *Ageratum conyzoides*, *Helianthus grosseserratus*, *Helianthus annuus*, *Helianthus tuberosus* and *Helianthus strumosus* (Table 2). The length of these 10 mitogenomes ranged from 194,298 to 363,324 bp. The largest mitogenome was from the *L. sativa* (363,324 bp), and the smallest was from the *A. giraldii* (194,298 bp) in this study. The length of *A. giraldii* was similar to two *Chrysanthemum* species, and they were all relatively small in the Asteraceae species. The GC content was relatively similar in terms of size, ranging from 44.89 to 45.66%. Meanwhile, we collated the number of PCGs in the 10 mitogenomes. The number of genes ranged from 24 *in H. annuus* to 35 *in D. hartwegii*. We determined the collinearity between *A. giraldii* and nine Asteraceae species by using the MAFFT (v7) online service (https://mafft.cbrc.jp/alignment/server/)^77^ to identify rearrangement among them. Using *A. giraldii* as a reference, dotplot analysis showed synteny fragment across all species (Fig. 2). Compared with the other seven Asteraceae species, *C. indicum* and *C. boreale* had larger synteny fragments. The largest fragments were about 27 kb in *C. indicum* and 42 kb in *C. boreale*. However, compared with the synteny fragments of the other seven Asteraceae species, the synteny fragments were smaller.

**Table 2:** Comparison of mitogenome and plastome in terms of size, GC content and number of PCGs in 10 Asteraceae plants.

Species	Mitogenome size (bp)	Plastome size (bp)	Size difference	GC content (%)	Number of PCGs
*Lactuca sativa*	363,324	152,765	210,559	45.35	32
*Diplostephium hartwegii*	277,718	151,994	125,724	44.89	35
*Chrysanthemum boreale*	211,002	151,012	59,990	45.36	35
*Chrysanthemum indicum*	208,097	150,972	57,125	45.41	33
*Artemisia giraldii*	194,298	151,072	43,226	45.66	32
*Ageratum conyzoides*	219,198	151,325	67,873	45.4	30
*Helianthus grosseserratus*	273,543	151,017	122,526	45.06	31
*Helianthus annuus*	300,945	151,104	149,841	45.05	27
*Helianthus tuberosus*	281,287	151,047	130,240	45.21	32
*Helianthus strumosus*	281,056	151,044	130,012	45.37	32

### Repeat sequence analysis

**Figure 3:**
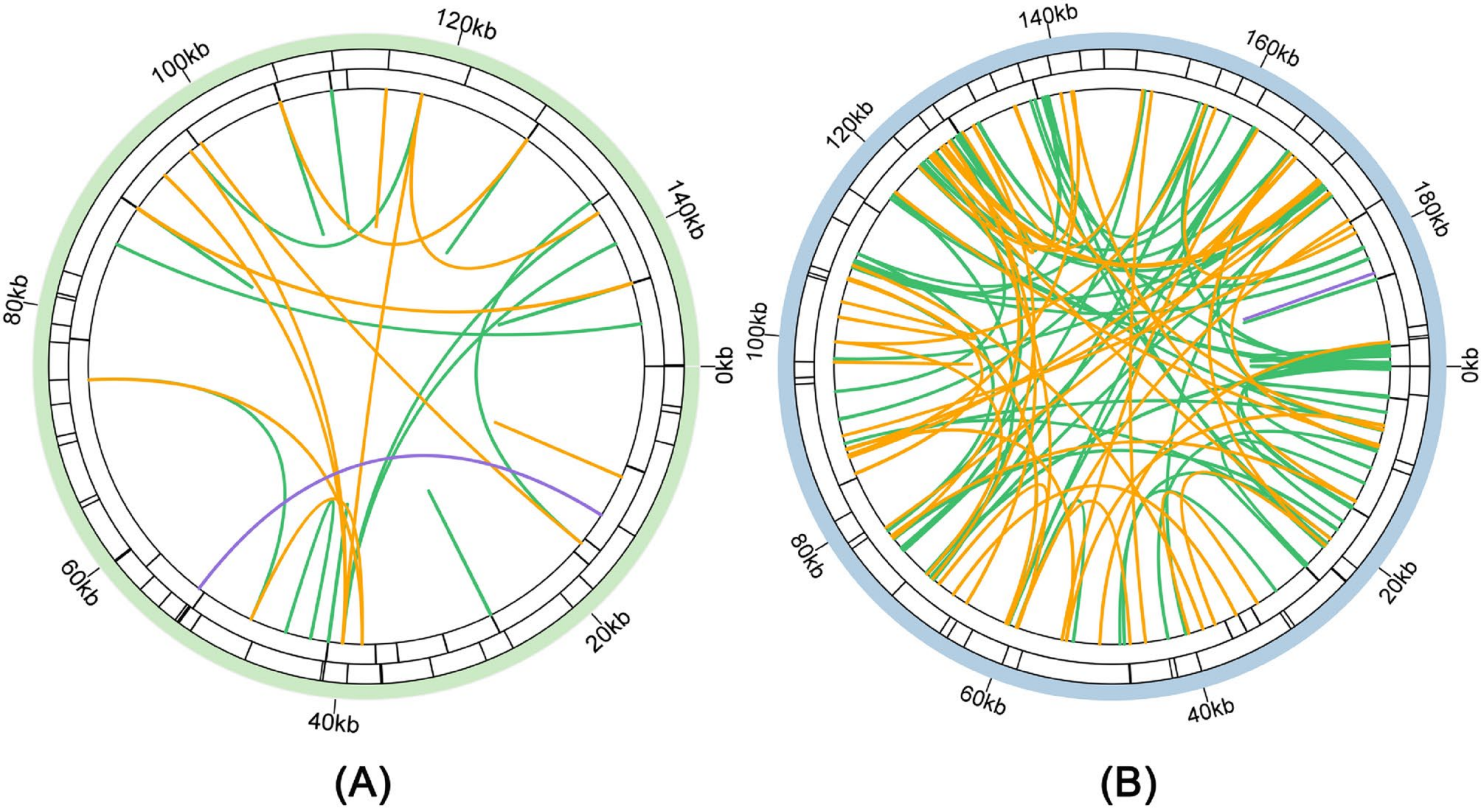
The repeat sequences of the *A. giraldii* organelle genomes. (**A**) The repeat sequences in the plastome. (**B**) The repeat sequences in the mitogenome. The first circle shows the dispersed repeats connected with green, orange, and purple arcs from the center going outward. The green, orange, and purple arcs represent the forward repeats, palindromic repeats, and reverse repeats, respectively. The next circle shows the tandem repeats as short bars. The third circle shows the microsatellite sequences as short bars. The scale is shown on the outermost circle, with intervals of 20 kb. The repeat sequences of the *A. giraldii* organelle genomes were visualized using the Circos package implemented in the TBtools.

In addition to difference in intergenic region, diversity in mitogenome size can be attributed to a large number of repeat sequences and foreign fragments^43,78^. Therefore, we analysed three common types of repeated sequences. Microsatellites (simple repeat sequences, SSRs), also called tandem repeats of 1–6 bp, are abundant in the genomes of higher organisms and usually show high levels of polymorphism^79^. Therefore, they are generally used as molecular markers for identifying similar species^80^. SSRs can be classified into different types according to repeat unit. For instance, SSRs are classified into mono-, di-, tri-, tetra-, penta- and hexanucleotide repeats according to the length of their major repeat units^81^. We identified 36 SSRs in the plastid sequence and 51 SSRs in the mitochondrial sequence (Fig. 3, Supplementary Tables S3, S4). The most abundant SSRs in the plastome were single-nucleotide SSRs, including 19(A) and 12(T), accounting for 79.49% of the total SSRs. However, the SSRs in the *A. giraldii* mitogenome were dominated by tetranucleotide polymers, which accounted for 43.14% of all repeats. The types of SSRs in the mitogenomes were more evenly represented than in the plastomes.

Tandem repeat sequences exist in the DNA of all organisms whose genomes have been sequenced. These sequences consist of multiple contiguous repeat units and exhibit extremely high mutation rates in eukaryotes and prokaryotes because they tend to gain or lose repeat units^82^. We identified 23 tandem repeats in the plastome and 15 in the mitogenome (Supplementary Tables S5, S6). The repeats can be further tested for their suitability as DNA fingerprinting markers.

In the *A. giraldii* plastome, we identified 38 dispersed repeats: 18 forward repeats, 19 palindromic repeats and 1 reverse repeat (Supplementary Table S7). All the dispersed repeats in the plastome were less than 100 bp, the longest was 60 bp and the shortest was 30 bp. However, the number of dispersed repeats in the mitogenome was larger than those in the plastome. In the mitogenome, we found 135 dispersed repeats comprising 85 forward repeats, 49 palindromic repeats and 1 reverse repeat. They accounted for 62.96%, 36.30% and 0.74% of all dispersed repeats, respectively (Supplementary Table S8). The length of the dispersed repeat sequences ranged from 30 to 248 bp, but only 17 were longer than 100 bp.

### Analysis of homologous sequences between two organelles

**Figure 4:**
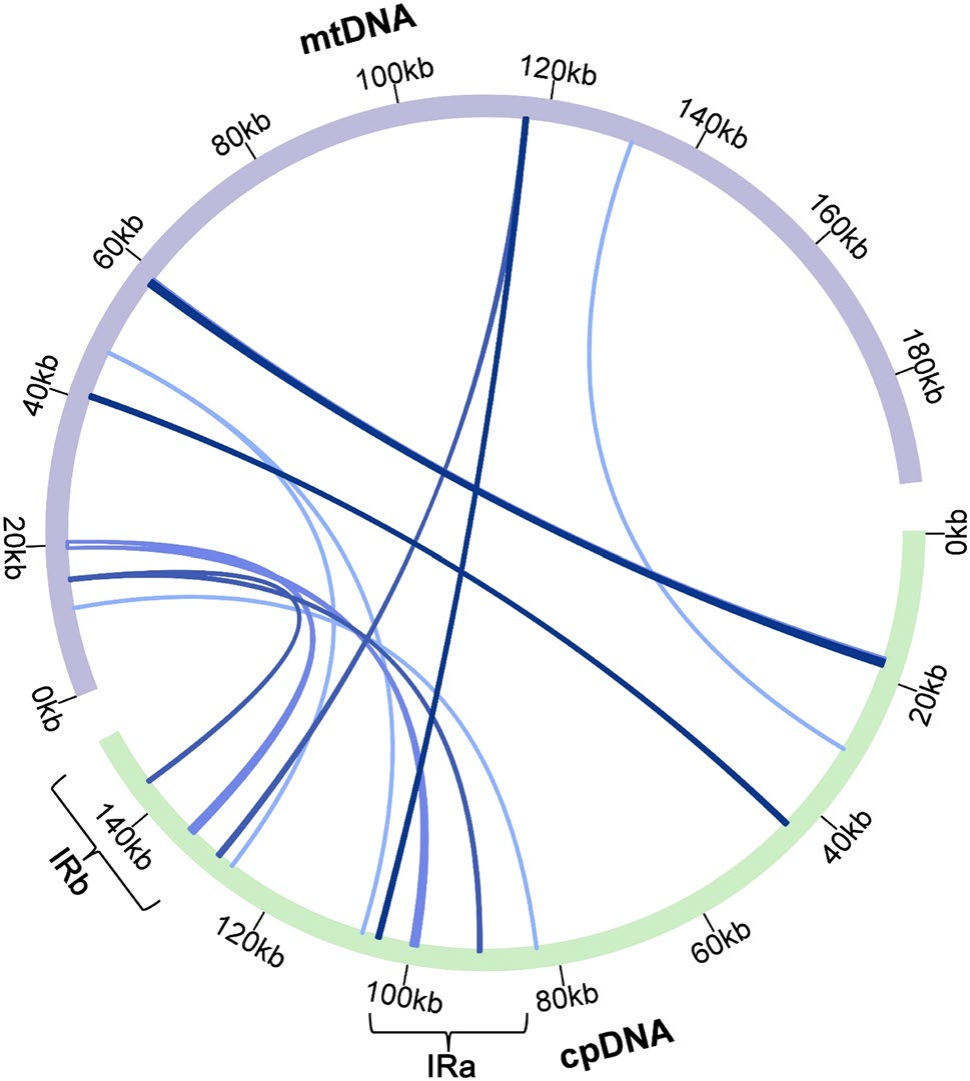
The homologous DNA sequences between the plastome and mitogenome of *A. giraldii*. The homologous DNA fragments were identified by comparing the plastome and the mitogenome sequences using the program BLASTn with the e-value cutoff of 1e-05. The purple and green circles represent the mitogenome (mtDNA) and plastome (cpDNA), respectively, and the inner blue arcs show the homologous DNA fragments. The scale is shown on the outermost circle, with intervals of 20 kb. The homologous sequences between the *A. giraldii* organelle genomes were visualized using the Circos package implemented in the TBtools.

The transfer of mitochondrial and plastid DNAs to the nucleus has been considered a part of the ongoing genome evolution and influences eukaryote evolution^83,84^. This process not only occurs from the organelle to the nucleus but also from the plastid DNA to the mitochondrial DNA^85,86^. For example, the plastid gene *rbc*L is transferred to the mitogenome numerous times during angiosperm evolution, and all evaluated sequences are pseudogenes^87^. To investigate whether plastid DNA is transferred to mitochondrial DNA, we used BLASTN^56^ to identify potential homologous sequences between the plastome and mitogenome in *A. giraldii*, and the cutoff e-value was 1e-05. Nine DNA fragments were found between two organelle genomes (Fig. 4, Supplementary Table S9). The total length of the nine fragments was 4806 bp and accounted for 2.47% of the whole mitogenome. The longest fragment was 888 bp in the mitogenome, and the shortest was 79 bp. The location of the nine homologous fragments in the mitochondrial and plastid genomes is shown in Supplementary Table S9.

### Phylogenetic inference analysis

**Figure 5:**
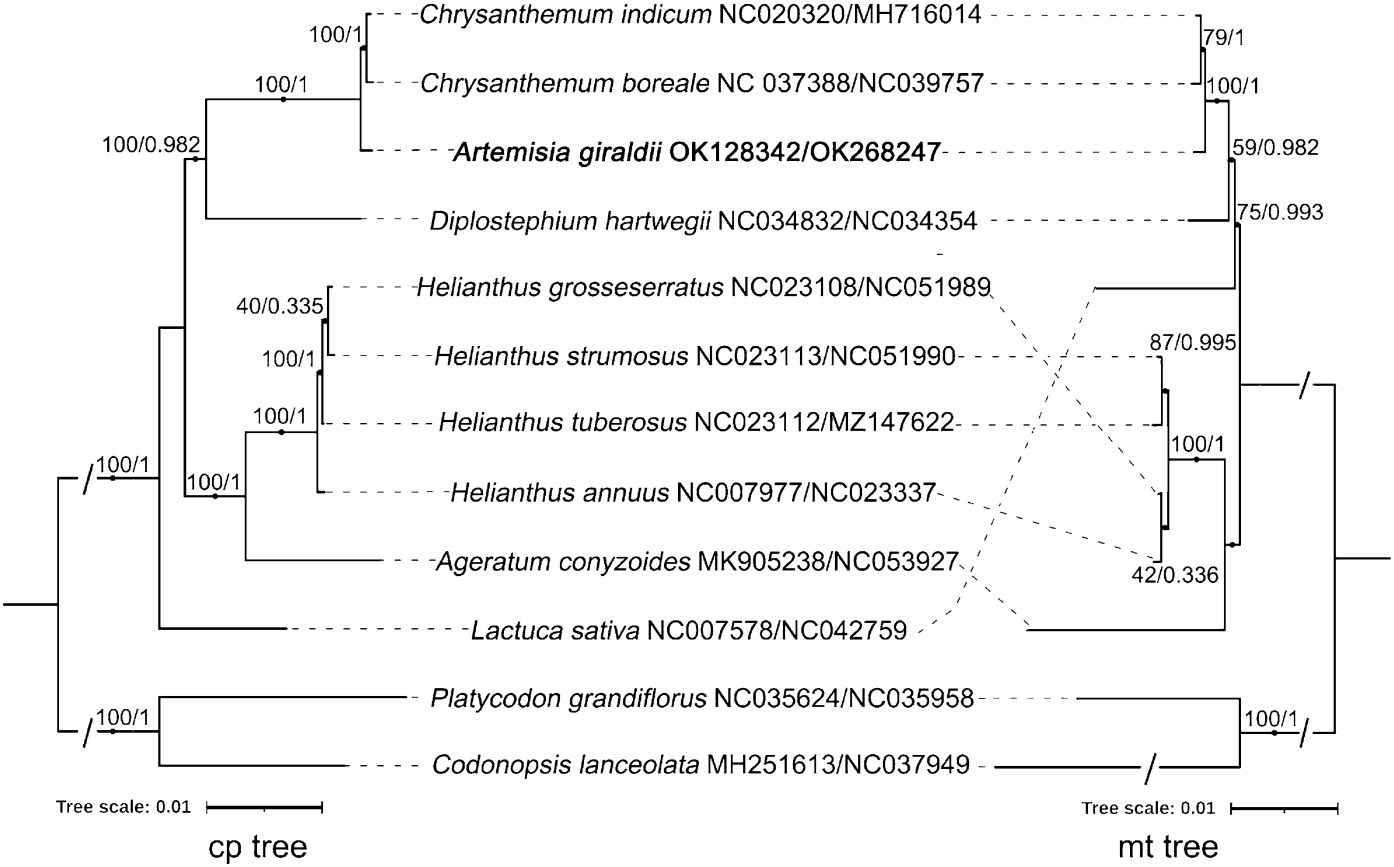
The phylogenetic relationships among *A. giraldii* and nine Asteraceae species using the maximum likelihood (ML) and Bayesian Inference (BI) methods. The sequence obtained from this study is highlighted in bold. The left is the phylogenetic tree constructed based on the coding sequences of 67 PCGs from the plastome. In contrast, on the right is the phylogenetic tree based on the coding sequences of 29 PCGs from the mitogenome. The numbers indicate the bootstrap values for the ML tree and Bayesian inference (BI) posterior probabilities for the BI tree, separated with a slash. The GenBank accession numbers of the plastomes and mitogenomes are shown after the Latin name of the related species, respectively. The length of the branch corresponds to the frequency of base substitutions. The phylogenetic trees constructed by the maximum likelihood (ML) and Bayesian Inference (BI) methods were visualized by iTOL (v5) (https://itol.embl.de/).

We constructed phylogenetic trees with the concatenated PCG sequences, using the maximum likelihood (ML) and BI methods (Fig. 5). The phylogenetic trees constructed with plastome and mitogenome sequences had minor differences in topological structures. In both trees, the 12 species were first divided into two main clades: a large clade composed of 10 Asteraceae species and a small clade composed of two outgroup species. *A. giraldii* was closely related to *C. indicum* and *C. boreale* in the two trees. In the mitochondrial genome tree, *H. grosseserratus* and *H. annuus* were clustered on one branch, and *H. strumosus* and *H. tuberosus* was clustered on another branch. However, in the plastome tree, *H. annuus* and *H. tuberosus* were separated into different branches, whereas *H. grosseserratus* and *H. strumosus* were clustered in a clade. The second difference was that *L. sativa* was located in different positions in the two trees. In the plastid tree, *L. sativa* was located in the outermost clade formed by the Asteraceae family. In the mitochondrial tree, *L. sativa* was located within the clade formed by the Asteraceae species (Fig. 5).

### Selective pressure analysis of *A. giraldii* mitogenomic genes

To determine which genes are subject to positive selection, we calculated the LRT p-value based on the lnL and np values of the null and alternative models for 28 protein-coding genes in the mitogenome. Then the likelihood ratio test (LRT) p-values were adjusted (Supplementary Table S10). The detailed analysis results can be found in Supplementary Table S11. The adjusted p-value of *ccm*Fc, *nad*1, *nad*6, *atp*9, *atp*1 and *rps*12 is below 0.05, suggesting these six genes are subject to positive selection.

### Molecular marker development

Based on the 18 plastome sequences of *Artemisia* species, we found one molecular marker for distinguishing among 18 *Artemisia* species (Supplementary Table S12). It was a pair of highly conserved regions that can be used for primer design. The regions amplified by the primer pairs contained one or more SNP and INDEL sites that can be used in distinguishing among the 18 *Artemisia* species. However, the lengths of the regions were about 30 kb, which is extremely long for practical uses.

### Analysis of hypervariable regions

**Figure 6:**
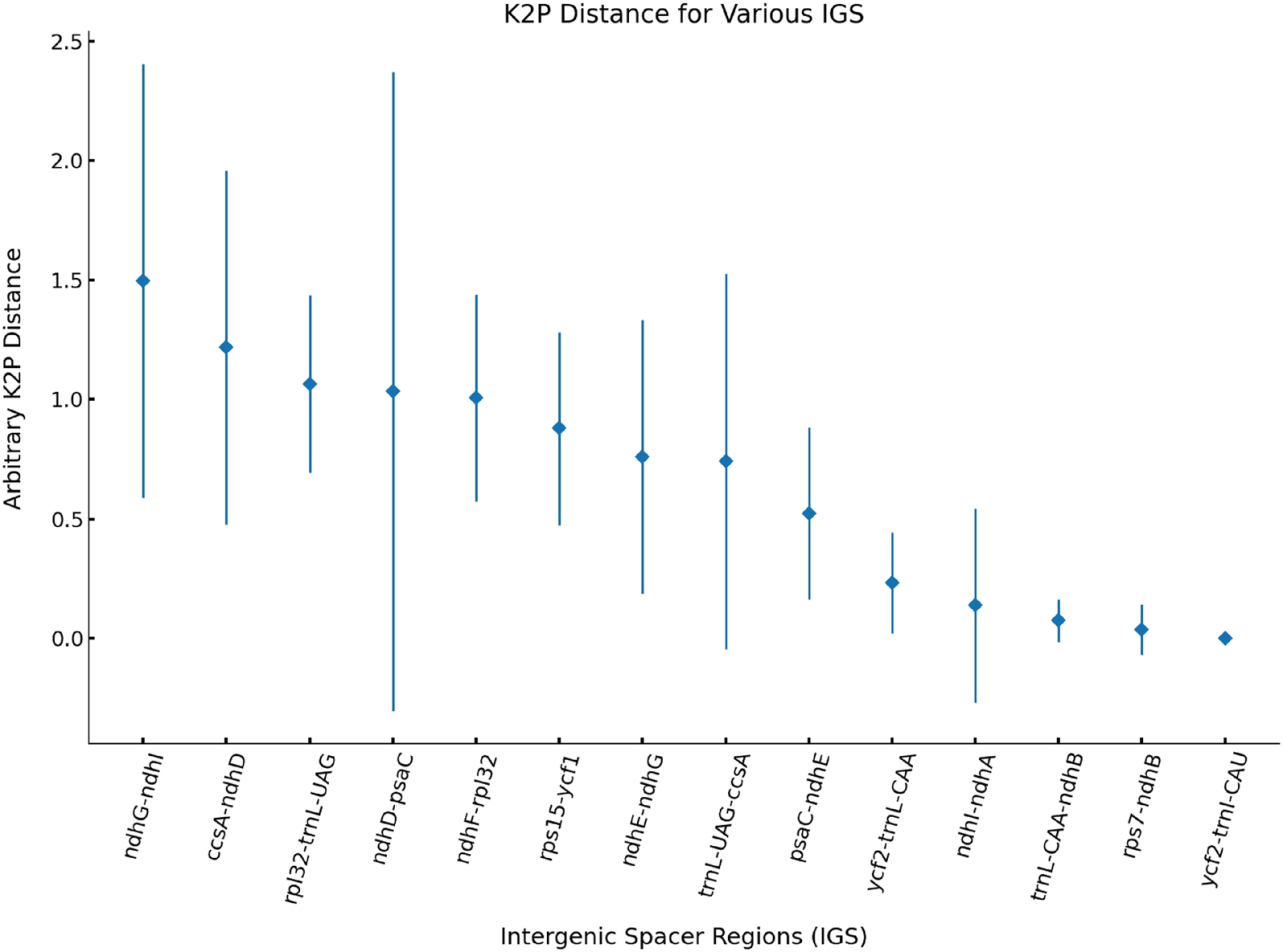
The hypervariable regions between the *Artemisia* genus. The horizontal direction represents the intergenic spacer regions that are highly variable among the 18 *Artemisia* species. The vertical direction is the arbitrary K2P distance of these regions. The square in the middle of each line represents the main distance of each intergenic spacer region.

A total of 14 IGS were hypervariable regions (Fig. 6). The top three regions: *ndh*G*-ndh*I, *ccs*A*-ndh*D and *rpl*32-*trn*L-UAG had K2p values of 1.50, 1.22 and 1.06, respectively. We first extracted the top three hypervariable regions and aligned them (Supplementary Fig. S7). However, the only two variant sites in *ccs*A*-ndh*D regions also existed in *rpl*32-*trn*L-UAG regions. Hence, we selected two regions: *ndh*G*-ndh*I and *rpl*32-*trn*L-UAG for molecular marker development. The variant sites in the two hypervariable regions can be used in distinguishing among the 18 species completely, including 11 SNPs and six indel sites (Supplementary Fig. S7). As indicated in Supplementary Fig. S7A, SNP 1–6 can be used in distinguishing among *Artemisia. hallaisanensis, Artemisia absinthium* var. *calcigena, Artemisia frigida*, *Artemisia maritima, Artemisia argyi* and *Artemisia fukudo* with other 17 species. Indel 1–3 can be used in discriminating among *Artemisia freyniana, Artemisia lactiflora* and *Artemisia gmelinii*. As demonstrated in Supplementary Fig. S7B, SNP7-11 can be used in identifying *A. frigida*, *Artemisia capillaris*, *Artemisia stolonifera*, *Artemisia montana* and *Artemisia scoparia*. Indel 4 and indel 5 can be used in identifying *Artemisia selengensis* and *A. annua* with other 17 species. After distinguishing among above 16 species, the remaining two species, *Artemisia ordosica* and *Artemisia tangutica* can be distinguished from each other by using indel 6.

## Discussion

*Artemisia giraldii* is a medicinal plant primarily used as a source of traditional medicines. Obtaining its genomic information is the critical step for understanding the biosynthesis of its active components. As the first step, we sequenced and assembled the mitogenome and plastome of *A. giraldii* in the current study. Then, we analysed the mitogenome and plastome's general features and compared them in detail.

In the plastome, two copies of IRs separate SSC and LSC regions^88^. When an IR region is present, homologous recombination occurs between the two copies and results in the frequent ‘flip’ inversion of the SSC region between the two copies, thus allowing two heterogeneous genomic orientations to coexist in a single plant with approximately the same frequency^89,90^.

In this study, we used two strategies to assemble the plastome of *A. giraldii*. The two strategies generated two assemblies that were identical, except that the SSC region was inverted (Supplementary Fig. S1A). The reverse and complement of the SSC region in the plastome assembly from Illumina and Nanopore data generated an assembly identical to that assembled by Illumina data (Supplementary Fig. S1B). Coverage depth is an indicator used in evaluating the correctness of an assembly in the mitochondria and the plastid genome assembling process. The drop of coverage depth is often considered a sign of misassembly. We observed several regions with low depths (Supplementary Figs. S2A and S3A,B). To determine whether assembling problems occurred, we visually examined the regions. The mapped results (Supplementary Figs. S2B and S3C) showed the reads sufficiently covered cover the regions, suggesting that the regions were correctly assembled. Further examination showed that the regions were AT rich. The AT-rich regions tend to be highly polymorphic and are error prone for long-read sequencing and result in a low coverage depth^91^.

The mitogenome of plants is much larger than the plastome^92^ because of frequent exchange with nuclear and chloroplast DNA^93^, repeat sequences, AT-rich non-coding regions, large introns and non-coding sequences^94^. The mitogenome size commonly ranges from 200 to 2400 kb in angiosperms^95^. By contrast, the plastome size commonly ranges from 100 to 200 kb. We compared the sizes of the mitogenomes and plastomes of plants released in GenBank to determine if the small difference between the two organelles is unusual. Our results showed a small difference in size between the mitogenome and plastome in *A. giraldii* among the 318 species having both mitogenomes and plastomes released in GenBank by August 1st, 2022 (Supplementary Table S13).

The size difference between the mitochondria and plastid genomes in *A. giraldii* was extremely small, only 43,226 bp, compared with the size difference in other species. Among the 318 species, 95 showed the smaller difference between mitogenome and plastome sizes than *A. giraldii*. 94 of the 95 species were algae and mosses. The only angiosperm plant having a smaller size difference was *Bidens pilosa* from Asteraceae, with 1236 bp. Actually, its size difference was the smallest among all pairs of mitogenomes and plastomes in this study. These observations suggested that mitogenome expansion develops along with plant evolution.

Among the Asteraceae species, *A. giraldii* had the second smallest difference. The other seven Asteraceae species, *Bidens parviflora*, *Bidens biternate, Bidens bipinnata*, *Chrysanthemum indicum, Chrysanthemum boreale, Bidens tripartite,* and *Ageratum conyzoides*, also had small size differences between their two organelle genomes, which were 44,511, 46,989, 46,990, 57,125, 59,990, 66,297 and 67,873 bp, respectively. This result indicated that small size difference is a common phenomenon in Asteraceae. The cause of this phenomenon has not yet been reported, and thus the specific mechanisms need to be further explored.

**Figure 7:**
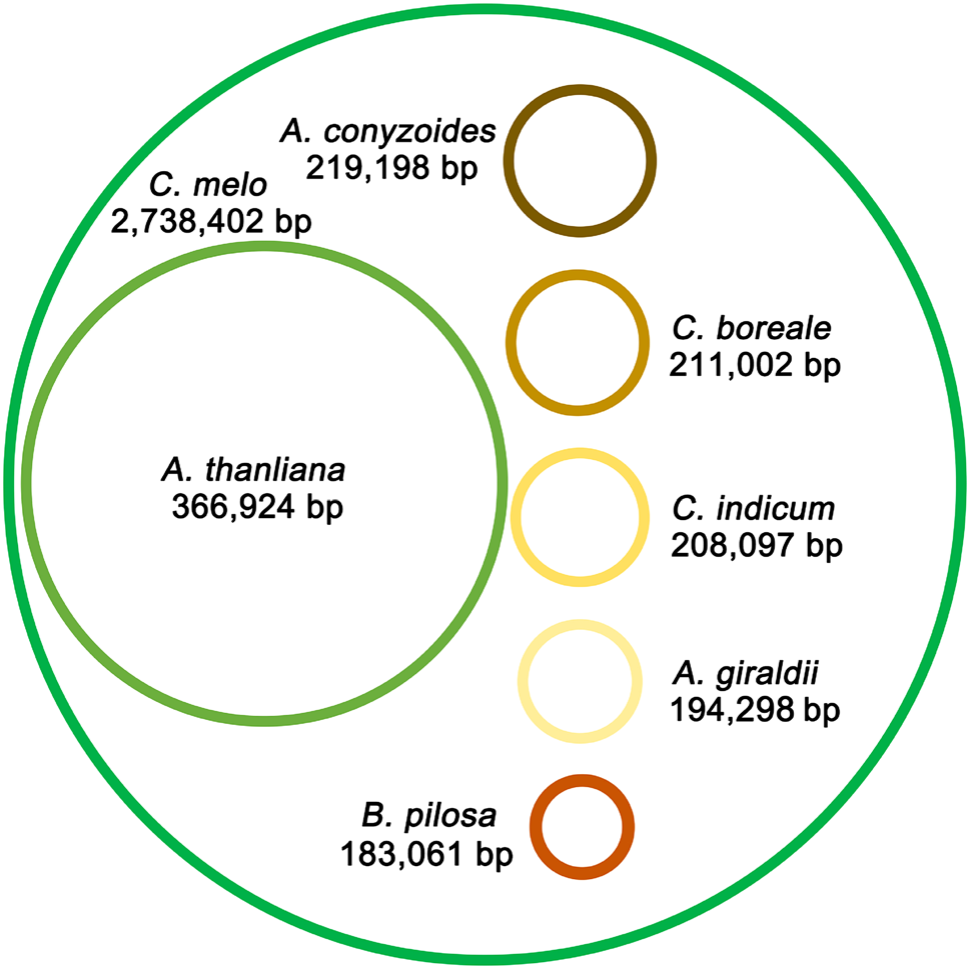
Comparison of mitogenome size between different species. Mitogenome sizes vary greatly among different plants. The outermost circle represents the size of the *Cucumis melo* mitogenome. The sizes of the circles are not drawn to scale.

We drew a figure to show the sizes of the seven most representative mitogenomes. The largest known mitogenome was obtained from *Cucumis melo*. The smallest known angiosperm mitogenome was obtained from *Bidens pilosa*. The sizes of four Asteraceae mitogenomes were in between (Fig. 7). The mitogenomes of different plants differ greatly in size.

We analysed the homologous sequence between mitogenome and plastome. Sequence migration is common in plants^96^. The plastid or nuclear DNA fragments can be inserted into mitochondrial DNA, resulting in an expanding mitogenome. These cp-derived mtDNAs can contain complete or partial PCG sequences^87,97^ and some tRNA sequences^86^. Frequently, these transfer sequences have no functions. We found nine homologous fragments between the plastid DNA and mitochondrial DNA. The total length of the nine fragments was 4806 bp and accounted for 2.47% of the whole mitogenome. To determine whether these homologous sequences originated from their common ancestor (vertical transfer) or were transferred from plastid to mitochondria (horizontal transfer), we determined whether these homologous sequences were present in the plastome and mitogenome of *C. boreale* with BLASTN. We found homologous sequences for eight fragments: F1, F2, F3, F5, F6, F7, F8 and F9 (Supplementary Table S9) in the plastome and mitogenome of *C. boreale*. We only found a homologous sequence for fragment F4 in the plastome of *C. boreale*. Therefore, we speculated that eight homologous fragments (F1, F2, F3, F5, F6, F7, F8 and F9) may have originated from their common ancestor and have been preserved throughout evolution. Another homologous fragment (F4) may have been transferred from the plastome to the mitogenome in *A. giraldii*. Thus, we suspected that a low degree of DNA exchange between the mitochondria and plastid DNAs is responsible for the low level of mitogenome expansion in *A. giraldii*.

Compared with plastomes and nuclear genes, the mitogenome has been rarely used in reconstruct phylogenies partly because of the slower nucleotide substitution rate and the difficulty of complete assembly and direct alignment^98,99^. We used the sequences of common genes to construct mitochondrial and plastid trees with ML and BI methods. *A. giraldii* was placed in the same locations in both trees. However, the plastid and mitochondrial trees differed in topology, particularly in the branch containing *L. sativa* and four *Helianthus* species. In the plastid tree, the *L. sativa* was located in the outermost clade formed by the Asteraceae family. In the mitochondrial tree, *L. sativa* was located within the clade formed by the Asteraceae species.

According to the taxonomy (https://www.ncbi.nlm.nih.gov/Taxonomy/Browser/wwwtax.cgi), *L. sativa* belongs to the Cichorioideae, whereas the other nine Asteraceae species belong to Asteroideae. Hence, the plastid tree was more in line with the taxonomic classification compared with the mitochondrial tree. *L. sativa* and Asteroideae species are located in different branches of the phylogenetic tree^100,101^. To further understand the relationship of mitochondrial genomes among 10 Asteraceae species, we aligned the mitogenome of *A. giraldii* (NC_064134.1) by using the BLASTN suite in NCBI (https://blast.ncbi.nlm.nih.gov/Blast.cgi). The results showed that the sequence similarity between 10 Asteraceae species was consistent with those shown in the mitochondrial tree (Supplementary Table S14). Compared with the four *Helianthus* species and *A. conyzoides*, the sequence similarity between *A. giraldii* and *L. sativa* was higher.

Previous report and sequence alignment results confirm the incongruence between the plastome tree and mitogenome tree for *L. sativa*. We hypothesised that the difference in topology between the two trees results from the inconsistent evolutionary rates of the plastome and mitogenome. Further analysis of the mitogenome of *L. sativa* is required to elucidate the incongruence. However, the support value between *H. grosseserratus* and *H. strumosus* in the plastome and between *H. grosseserratus* and *H. annuus* were less than 50 because of the high sequence similarity among *Helianthus* species, making the branches inseparable. The *A. giraldii* reported in this study had the same branch structure in the two trees and had a high support value, suggesting high credibility for its evolutionary relationship. The closest relatives to *A. giraldii* were *C. indicum* and *C. boreale*. This result is consistent with their taxonomic relationship, as they both belong to Artemisiinae. The collinearity results confirmed this conclusion. *C. indicum* and *C. boreale* had a larger synteny fragment than the *A. giraldii* mitogenome. Overall, the results revealed that the gene orders on the mitogenomes of the 10 Asteraceae species differed significantly.

Most mitochondrial genes are highly conserved and have undergone neutral and negative selection. The selective pressure analysis is commonly used in identifying positively or negatively selected genes to adapt to a particular lifestyle. In this analysis, the adjusted p-values of *ccm*Fc, *nad*1, *nad*6, *atp*9, *atp*1 and *rps*12 were below 0.05, suggesting that these genes underwent positive selection in the evolution process. The other 22 genes were more conserved and not subject to positive selection. The adjusted p-values of *ccm*Fc and *nad*1 were 0, suggesting that they are subject to strong positive selection. *ccm*Fc was a protein similar to the C-terminal part of the bacterial *ccm*F. It is involved in cytochrome c maturation and is present in a large-sized complex in wheat mitochondria^102^. *nad*1 is one of the NADH dehydrogenases and plays an important role in mitochondrial electron transport^103^. Given the limited availability of mitogenomes in *Artemisia*, we used the plastome sequences of 18 *Artemisia* species to predict one pair of primers that potentially amplify a variable DNA region to distinguish among 18 *Artemisia* species. However, the length of the predicted amplified fragment was extremely long to validate. We concluded that this molecular marker may not be applicable to distinguish them. Instead, we analysed the hypervariable regions of the 18 species to obtain available molecular markers. Owing to the large number of species, the variant site in one hypervariable region cannot be used in distinguishing 18 species from one another. The variant site in *ccs*A-*ndh*D is present in *rpl*32-*trn*L-UAG, and thus the 17 variant sites in the two hypervariable regions (*ndh*G*-ndh*I and *rpl*32-*trn*L-UAG) were combined (11 SNPs and six indels). We were able to completely distinguish among 18 *Artemisia* species (Supplementary Fig. S7). Further experimental verification of these molecular markers is needed.

## Conclusions

In this study, we assembled the mitogenome and plastome of *A. giraldii* for the first time. Phylogenetic analysis showed that the branch locations of *A. giraldii* in the phylogenetic trees constructed with the mitochondrial and plastid protein sequences were identical, suggesting the possible co-evolution of the genomes from the two organelles. Homologous sequence analysis identified nine homologous fragments between two organelles, and one fragment might have transferred from the plastome into the mitogenome. This study may provide a reference for studying the evolutionary relationship between mitochondria and plastids in Asteraceae species.

## Supplementary Information

Supplementary Information.

## Data Availability

The plastome and mitogenome sequences of *A. giraldii* reported in this article are available in GenBank (https://www.ncbi.nlm.nih.gov/) with accession numbers OK128342 and NC_064134.1, respectively. The raw data have been submitted to the SRA database (BioSample: SAMN25050459; BioProject: PRJNA798221; SRA: SRR17652243). The sample has been deposited in the Institute of Medicinal Plant Development (Beijing, China) with accession number implad201910017.

## References

[1] Bremer K, Humphries CJ (1993). Generic monograph of the Asteraceae-Anthemideae. Bull. Nat. Hist. Museum Bot. Ser..

[2] Martín J, Torrell M, Korobkov A, Vallès J (2003). Palynological features as a systematic marker in *Artemisia* L. and related genera (Asteraceae, Anthemideae)-II: Implications for Subtribe Artemisiinae delimitation. Plant Biol..

[3] Watson LE, Bates PL, Evans TM, Unwin MM, Estes JR (2002). Molecular phylogeny of subtribe Artemisiinae (Asteraceae), including Artemisia and its allied and segregate genera. BMC Evol. Biol..

[4] Iranshahi, M., Emami, S. A. & MAHMOUD, S. M. Detection of sesquiterpene lactones in ten Artemisia species population of Khorasan provinces. (2007).

[5] Abad MJ, Bedoya LM, Apaza L, Bermejo P (2012). The *Artemisia* L. genus: A review of bioactive essential oils. Molecules.

[6] Koul B, Taak P, Kumar A, Khatri T, Sanyal I (2018). The *Artemisia* genus: A review on traditional uses, phytochemical constituents, pharmacological properties and germplasm conservation. J. Glycomics Lipidomics.

[7] Zheng W, Tan R, Yang L, Liu Z (1996). A new antimicrobial sesquiterpene lactone from *Artemisia giraldii*. Spectrosc. Lett..

[8] Zheng W, Tan R, Yang L, Liu Z (1996). Two flavones from *Artemisia giraldii* and their antimicrobial activity. Planta Med..

[9] Obistioiu D (2014). Chemical characterization by GC-MS and in vitro activity against *Candida albicans* of volatile fractions prepared from *Artemisia dracunculus*, *Artemisia abrotanum*, *Artemisia absinthium* and *Artemisia vulgaris*. Chem. Cent. J..

[10] Shafi G (2012). *Artemisia absinthium* (AA): A novel potential complementary and alternative medicine for breast cancer. Mol. Biol. Rep..

[11] Mojarrab M, Emami S, Gheibi S, Taleb A, Heshmati Afshar F (2016). Evaluation of anti-malarial activity of *Artemisia **turcomanica* and *A. **kopetdaghensis* by cell-free β-hematin formation assay. Res. J. Pharmacogn..

[12] Taherkhani M (2014). In vitro cytotoxic activity of the essential oil extracted from *Artemisia absinthium*. Iran. J. Toxicol..

[13] Altunkaya, A., Yıldırım, B., Ekici, K. & Terzioğlu, Ö. Determining essential oil composition, antibacterial and antioxidant activity of water wormwood extracts. (2018).

[14] Rajeshkumar P, Hosagoudar V (2012). Mycorrhizal fungi of *Artemisia japonica*. Bull. Basic Appl. Plant Biol..

[15] Klayman DL (1985). Qinghaosu (artemisinin): An antimalarial drug from China. Science.

[16] Tu Y (2011). The discovery of artemisinin (qinghaosu) and gifts from Chinese medicine. Nat. Med..

[17] Liu F, Yang J, Zhang P (2012). Relationships between geographical distribution of *Artemisia giraldii* and cli-mate. J. Arid Land Resour. Environ..

[18] Zhicheng Z, Donglin J, Ming Y (1997). Preliminary study on the biomass of *Artemisia giraldii* community. Grassland China.

[19] Tan R (1999). Mono-and sesquiterpenes and antifungal constituents from *Artemisia* species. Planta Med..

[20] Zheng W, Tan R, Liu Z (1996). A analysis of terpenoids in petrol extracts of eight *Artemisia* species. J.-Nanjing Univ. Nat. Sci. Ed..

[21] Chu S-S, Liu Z-L, Du S-S, Deng Z-W (2012). Chemical composition and insecticidal activity against *Sitophilus **zeamais* of the essential oils derived from *Artemisia giraldii* and *Artemisia **subdigitata*. Molecules.

[22] Gray MW (1992). The endosymbiont hypothesis revisited. Int. Rev. Cytol..

[23] Hikosaka K (2010). Divergence of the mitochondrial genome structure in the apicomplexan parasites, Babesia and Theileria. Mol. Biol. Evol..

[24] Smith DR, Keeling PJ (2015). Mitochondrial and plastid genome architecture: Reoccurring themes, but significant differences at the extremes. Proc. Natl. Acad. Sci..

[25] Mower JP, Sloan DB, Alverson AJ (2012). Plant mitochondrial genome diversity: The genomics revolution. Plant Genome Divers..

[26] Shtratnikova VY, Schelkunov MI, Penin AA, Logacheva MD (2020). Mitochondrial genome of the nonphotosynthetic mycoheterotrophic plant *Hypopitys*
*monotropa*, its structure, gene expression and RNA editing. PeerJ.

[27] Yu R (2022). The minicircular and extremely heteroplasmic mitogenome of the holoparasitic plant Rhopalocnemis phalloides. Curr. Biol..

[28] Yudina SV (2021). Comparative analysis of plastid genomes in the non-photosynthetic genus *Thismia* reveals ongoing gene set reduction. Front. Plant Sci..

[29] Smith DR (2010). The *Dunaliella** salina* organelle genomes: Large sequences, inflated with intronic and intergenic DNA. BMC Plant Biol..

[30] Ladoukakis ED, Zouros E (2017). Evolution and inheritance of animal mitochondrial DNA: Rules and exceptions. J. Biol. Res.-Thessaloniki.

[31] Boore JL (1999). Animal mitochondrial genomes. Nucleic Acids Res..

[32] Quetier F, Vedel F (1977). Heterogeneous population of mitochondrial DNA molecules in higher plants. Nature.

[33] Bendich AJ (1993). Reaching for the ring: The study of mitochondrial genome structure. Curr. Genet..

[34] Sloan DB (2013). One ring to rule them all? Genome sequencing provides new insights into the ‘master circle’ model of plant mitochondrial DNA structure. New Phytol..

[35] Adams KL, Qiu Y-L, Stoutemyer M, Palmer JD (2002). Punctuated evolution of mitochondrial gene content: High and variable rates of mitochondrial gene loss and transfer to the nucleus during angiosperm evolution. Proc. Natl. Acad. Sci..

[36] Adams KL, Palmer JD (2003). Evolution of mitochondrial gene content: Gene loss and transfer to the nucleus. Mol. Phylogenet. Evol..

[37] Palmer JD, Shields CR (1984). Tripartite structure of the *Brassica campestris* mitochondrial genome. Nature.

[38] Lonsdale DM, Hodge TP, Fauron CM-R (1984). The physical map and organisation of the mitochondrial genome from the fertile cytoplasm of maize. Nucleic Acids Res..

[39] Mower JP, Case AL, Floro ER, Willis JH (2012). Evidence against equimolarity of large repeat arrangements and a predominant master circle structure of the mitochondrial genome from a monkeyflower (*Mimulus **guttatus*) lineage with cryptic CMS. Genome Biol. Evol..

[40] Kozik A (2019). The alternative reality of plant mitochondrial DNA: One ring does not rule them all. PLoS Genet..

[41] Maréchal A, Brisson N (2010). Recombination and the maintenance of plant organelle genome stability. New Phytol..

[42] Mackenzie, S. A. The unique biology of mitochondrial genome instability in plants. *Plant Mitochondria.* 36 (2007).

[43] Alverson AJ (2010). Insights into the evolution of mitochondrial genome size from complete sequences of *Citrullus **lanatus* and *Cucurbita pepo* (Cucurbitaceae). Mol. Biol. Evol..

[44] Ogihara Y (2005). Structural dynamics of cereal mitochondrial genomes as revealed by complete nucleotide sequencing of the wheat mitochondrial genome. Nucleic Acids Res..

[45] Kempken F, Pring D (1999). Progress in Botany.

[46] Bolger AM, Lohse M, Usadel B (2014). Trimmomatic: A flexible trimmer for Illumina sequence data. Bioinformatics.

[47] Jin J-J (2020). GetOrganelle: A fast and versatile toolkit for accurate de novo assembly of organelle genomes. Genome Biol..

[48] Li H (2016). Minimap and miniasm: Fast mapping and de novo assembly for noisy long sequences. Bioinformatics.

[49] Shi L (2019). CPGAVAS2, an integrated plastome sequence annotator and analyzer. Nucleic Acids Res..

[50] Xia Y (2016). The complete chloroplast genome sequence of *Chrysanthemum indicum*. Mitochondrial DNA Part A.

[51] Tillich M (2017). GeSeq–versatile and accurate annotation of organelle genomes. Nucleic Acids Res..

[52] Wang S (2018). Assembly of a complete mitogenome of *Chrysanthemum **nankingense* using Oxford Nanopore long reads and the diversity and evolution of Asteraceae mitogenomes. Genes.

[53] Chan PP, Lowe TM (2019). Gene Prediction.

[54] Lewis SE (2002). Apollo: A sequence annotation editor. Genome Biol..

[55] Greiner S, Lehwark P, Bock R (2019). OrganellarGenomeDRAW (OGDRAW) version 1.3.1: Expanded toolkit for the graphical visualization of organellar genomes. Nucleic Acids Res..

[56] Chen Y, Ye W, Zhang Y, Xu Y (2015). High speed BLASTN: An accelerated MegaBLAST search tool. Nucleic Acids Res..

[57] Zhang H, Meltzer P, Davis S (2013). RCircos: An R package for Circos 2D track plots. BMC Bioinform..

[58] Chen C (2020). TBtools: An integrative toolkit developed for interactive analyses of big biological data. Mol. Plant.

[59] Beier S, Thiel T, Münch T, Scholz U, Mascher M (2017). MISA-web: A web server for microsatellite prediction. Bioinformatics.

[60] Benson G (1999). Tandem repeats finder: A program to analyze DNA sequences. Nucleic Acids Res..

[61] Kurtz S (2001). REPuter: The manifold applications of repeat analysis on a genomic scale. Nucleic Acids Res..

[62] Zhang D (2020). PhyloSuite: An integrated and scalable desktop platform for streamlined molecular sequence data management and evolutionary phylogenetics studies. Mol. Ecol. Resour..

[63] Katoh K, Standley DM (2013). MAFFT multiple sequence alignment software version 7: Improvements in performance and usability. Mol. Biol. Evol..

[64] Castresana J (2000). Selection of conserved blocks from multiple alignments for their use in phylogenetic analysis. Mol. Biol. Evol..

[65] Minh BQ (2020). IQ-TREE 2: New models and efficient methods for phylogenetic inference in the genomic era. Mol. Biol. Evol..

[66] Ivica L, Peer B (2021). Interactive Tree Of Life (iTOL) v5: An online tool for phylogenetic tree display and annotation. Nucleic Acids Res..

[67] Darriba D, Taboada GL, Doallo R, Posada D (2012). jModelTest 2: More models, new heuristics and parallel computing. Nat. Methods.

[68] Ronquist F (2012). MrBayes 3.2: Efficient Bayesian phylogenetic inference and model choice across a large model space. Syst. Biol..

[69] Gao F (2019). EasyCodeML: A visual tool for analysis of selection using CodeML. Ecol. Evol..

[70] Yang Z, Nielsen R (2002). Codon-substitution models for detecting molecular adaptation at individual sites along specific lineages. Mol. Biol. Evol..

[71] Thissen D, Steinberg L, Kuang D (2002). Quick and easy implementation of the Benjamini–Hochberg procedure for controlling the false positive rate in multiple comparisons. J. Educ. Behav. Stat..

[72] Tiayyba R (2011). ecoPrimers: Inference of new DNA barcode markers from whole genome sequence analysis. Nucleic Acids Res..

[73] Larkin MA (2007). Clustal W and Clustal X version 2.0. Bioinformatics.

[74] Rice P, Longden I, Bleasby A (2000). EMBOSS: The European molecular biology open software suite. Trends Genet..

[75] Milne I (2013). Using Tablet for visual exploration of second-generation sequencing data. Brief. Bioinform..

[76] Wick RR, Schultz MB, Zobel J, Holt KE (2015). Bandage: Interactive visualization of de novo genome assemblies. Bioinformatics.

[77] Katoh K, Rozewicki J, Yamada KD (2019). MAFFT online service: Multiple sequence alignment, interactive sequence choice and visualization. Brief. Bioinform..

[78] Park S (2015). Dynamic evolution of Geranium mitochondrial genomes through multiple horizontal and intracellular gene transfers. New Phytol..

[79] Ellegren H (2004). Microsatellites: Simple sequences with complex evolution. Nat. Rev. Genet..

[80] Guang X-M (2019). IDSSR: An efficient pipeline for identifying polymorphic microsatellites from a single genome sequence. Int. J. Mol. Sci..

[81] Fan H, Chu J-Y (2007). A brief review of short tandem repeat mutation. Genomics Proteomics Bioinform..

[82] Bichara M, Wagner J, Lambert I (2006). Mechanisms of tandem repeat instability in bacteria. Mutat. Res. Fundam. Mol. Mech. Mutagen..

[83] Richly E, Leister D (2004). NUMTs in sequenced eukaryotic genomes. Mol. Biol. Evol..

[84] Huang CY, Grunheit N, Ahmadinejad N, Timmis JN, Martin W (2005). Mutational decay and age of chloroplast and mitochondrial genomes transferred recently to angiosperm nuclear chromosomes. Plant Physiol..

[85] Sugiyama Y (2005). The complete nucleotide sequence and multipartite organization of the tobacco mitochondrial genome: Comparative analysis of mitochondrial genomes in higher plants. Mol. Genet. Genomics.

[86] Notsu Y (2002). The complete sequence of the rice (*Oryza sativa* L.) mitochondrial genome: Frequent DNA sequence acquisition and loss during the evolution of flowering plants. Mol. Genet. Genomics.

[87] Cummings MP, Nugent JM, Olmstead RG, Palmer JD (2003). Phylogenetic analysis reveals five independent transfers of the chloroplast gene rbcL to the mitochondrial genome in angiosperms. Curr. Genet..

[88] Knox EB (2014). The dynamic history of plastid genomes in the *Campanulaceae* sensu lato is unique among angiosperms. Proc. Natl. Acad. Sci..

[89] Palmer JD (1983). Chloroplast DNA exists in two orientations. Nature.

[90] Stein DB, Palmer JD, Thompson WF (1986). Structural evolution and flip-flop recombination of chloroplast DNA in the fern genus *Osmunda*. Curr. Genet..

[91] Delahaye C, Nicolas J (2021). Sequencing DNA with nanopores: Troubles and biases. PLoS ONE.

[92] Dong S (2018). The complete mitochondrial genome of the early flowering plant *Nymphaea **colorata* is highly repetitive with low recombination. BMC Genomics.

[93] Timmis JN, Ayliffe MA, Huang CY, Martin W (2004). Endosymbiotic gene transfer: Organelle genomes forge eukaryotic chromosomes. Nat. Rev. Genet..

[94] Unseld M, Marienfeld JR, Brandt P, Brennicke A (1997). The mitochondrial genome of *Arabidopsis thaliana* contains 57 genes in 366,924 nucleotides. Nat. Genet..

[95] Kubo T, Newton KJ (2008). Angiosperm mitochondrial genomes and mutations. Mitochondrion.

[96] Wang X-C, Chen H, Yang D, Liu C (2018). Diversity of mitochondrial plastid DNAs (MTPTs) in seed plants. Mitochondrial DNA Part A.

[97] Clifton SW (2004). Sequence and comparative analysis of the maize NB mitochondrial genome. Plant Physiol..

[98] Van de Paer C, Bouchez O, Besnard G (2018). Prospects on the evolutionary mitogenomics of plants: A case study on the olive family (*Oleaceae*). Mol. Ecol. Resour..

[99] Vargas OM, Ortiz EM, Simpson BB (2017). Conflicting phylogenomic signals reveal a pattern of reticulate evolution in a recent high-Andean diversification (Asteraceae: Astereae: Diplostephium). New Phytol..

[100] Kim J-K (2016). The complete chloroplast genome sequence of the *Taraxacum officinale* FH Wigg (Asteraceae). Mitochondrial DNA Part B.

[101] Walker JF, Zanis MJ, Emery NC (2014). Comparative analysis of complete chloroplast genome sequence and inversion variation in *Lasthenia*
*burkei* (Madieae, Asteraceae). Am. J. Bot..

[102] Giegé P, Rayapuram N, Meyer EH, Grienenberger JM, Bonnard G (2004). CcmFC involved in cytochrome c maturation is present in a large sized complex in wheat mitochondria. FEBS Lett..

[103] Weiss H, Friedrich T, Hofhaus G, Preis D (1991). The respiratory-chain NADH dehydrogenase (complex I) of mitochondria. EJB Rev..

