# Supplementary material for: Comparative analysis of the plastid and mitochondrial genomes of Artemisia giraldii Pamp
Source: Sci Rep. 2022 Aug 17;12:13931. doi: 10.1038/s41598-022-18387-2 (PMC9385723; doi:10.1038/s41598-022-18387-2)
Supplement: Supplementary file 1 — Supplementary Information. [file 41598_2022_18387_MOESM1_ESM.pdf]

Supplementary information

Title:

Comparative analysis of the plastid and mitochondrial genomes of *Artemisia giraldii* Pamp.

Authors:

Jingwen Yue[1, 2#], Qianqi Lu[1#], Yang Ni[2], Pinghua Chen[1*], Chang Liu[2*]

Author affiliation:

[1]College of Agriculture, Fujian Agriculture and Forestry University, Fuzhou, Fujian Province, Fuzhou 350002, P. R. China.

[2]Institute of Medicinal Plant Development, Chinese Academy of Medical Sciences, Peking Union Medical College, Beijing, China

[#] **These authors contributed equally to this work**

[*] **Corresponding Author:**

Pinghua Chen: No.15, Shangxiadian Road, Fuzhou, Fujian province, 350002, P.R.China.

Email: phcemail@126.com (PHC); Phone: +86-0591-83789177, Fax: +86-0591-83768242;

Chang Liu: No. 151, Malianwa North Road, Haidian District, Beijing 100093, P.R.China

Email address: cliu6688@yahoo.com; Phone: +86-10-57833111; Fax: +86-10-62899715;

Table and figure legend:

Table S1 List of Genes of the *A. giraldii* plastome.

Category of genes	Group of genes	Name of genes
	rRNA	*rrn*4.5S (×2), *rrn*5S (×2), *rrn*16S (×2), *rrn*23S (×2)
	tRNA	*trn*A-UGC (×2), *trn*C-GCA, *trn*D-GUC, *trn*E-UUC, *trn*F-GAA, *trn*fM-CAU, *trn*G-GCC, *trn*G-UCC, *trn*H-GUG, *trn*I-CAU (x2), *trn*I-GAU (x2), *trn*K-UUU, *trn*L-CAA (x2), *trn*L-UAA, *trn*L-UAG, *trn*M-CAU, *trn*N-GUU (x2), *trn*P-UGG, *trn*Q-UUG, *trn*R-UCU, *trn*R-ACG (x2), *trn*S-GCU, *trn*S-GGA, *trn*S-UGA, *trn*T-GGU, *trn*T-UGU, *trn*V-GAC (x2), *trn*V-UAC, *trn*W-CCA, *trn*Y-GUA
photosynthesis	Subunits of ATP synthase	*atp*A, *atp*B, *atp*E, *atp*F, *atp*H, *atp*I
	Subunits of photosystem II	*Ihb*A, *psb*A, *psb*B, *psb*C, *psb*D, *psb*E, *psb*F, *psb*I, *psb*J, *psb*K, *psb*M, *psb*N, *psb*T, *ycf*3
	Subunits of cytochrome b/f complex	*pet*A, *pet*B, *pet*D, *pet*G, *pet*L, *pet*N
	Subunits of photosystem I	*psa*A, *psa*B, *psa*C, *psa*I, *psa*J
	Subunit of rubisco	*rbc*L
	NADH dehydrogenase-like complex	*ndh*A, *ndh*B (×2), *ndh*C, *ndh*D, *ndh*E, *ndh*F, *ndh*G, *ndh*H, *ndh*I, *ndh*J, *ndh*K
Self-replication	Large subunit of ribosome	*rpl*14, *rpl*16, *rpl*2 (×2), *rpl*20, *rpl*22, *rpl*23 (×2), *rpl*32, *rpl*33, *rpl*36
	DNA dependent RNA polymerase	*rpo*A, *rpo*B, *rpo*C1, *rpo*C2
	Small subunit of ribosome	*rps*11, *rps*12 (×2), *rps*14, *rps*15, *rps*16, *rps*18, *rps*19, *rps*2,

		*rps*3, *rps*4, *rps*7 (×2), *rps*8
Other genes	Subunit of Acetyl-CoA-carboxylase	*acc*D
	c-type cytochrome synthesis gene	*ccs*A
	Envelop membrane protein	*cem*A
	Protease	*clp*P
	Translational initiation	*inf*A
	Maturase	*mat*K
Unknown	Conserves open reading frames	*ycf*1, *ycf*2 (×2), *ycf*4, *ycf*15 (×2)

Table S2 The lengths of introns and exons for the splitting genes in the *A. giraldii* plastome.

Gene	Strand	Start	End	Exon I	Intron I	Exon II	Intron II	Exon III
*rpl*16	+	1575	3001	9	1019	399		
*pet*D	-	6459	7616	8	675	475		
*pet*B	-	7805	9215	6	763	642		
*clp*P	+	12135	14135	71	797	294	613	226
*trn*V-UAC	+	31152	31798	38	572	37		
*trn*L-UAA	-	35748	36258	37	424	50		
*ycf*3	+	39062	41005	124	702	230	735	153
*trn*G-UCC	+	52130	52929	23	730	47		
*atp*F	-	54941	56194	145	699	410		
*rpo*C1	-	64108	66909	432	732	1638		
*rps*16	+	76539	77646	40	871	197		
*trn*K-UUU	+	78491	81111	37	2549	35		
*rpl*2	-	82954	84443	397	662	431		
*ndh*B	-	92992	95194	777	670	756		
*trn*I-GAU	+	100718	101570	43	775	35		
*trn*A-UGC	+	101635	102519	38	812	35		
*ndh*A	-	117567	119720	553	1062	539		
*trn*A-UGC	-	131392	132276	38	812	35		
*trn*I-GAU	-	132341	133193	43	775	35		
*ndh*B	+	138717	140919	777	670	756		
*rpl*2	+	149468	150957	397	662	431		

Table S3 Microsatellite repeats in the *A. giraldii* plastome. The structure of Microsatellite repeats is presented as the repeat unit surrounded by parenthesis followed by the number of the repeat unit in the repeat sequence.

Type	Structure of Microsatellite Repeats	Size (bp)	Start	End
p1	(A)15	15	3102	3116
p1	(A)12	12	3606	3617
p1	(A)10	10	6025	6034
p1	(A)16	16	13549	13564
p3	(AAT)6	18	18670	18687
p1	(A)13	13	20750	20762
p1	(A)11	11	26215	26225
p1	(A)15	15	28439	28453
p1	(A)12	12	32643	32654
c	(T)11cttattactagttctatatcgct agattcatcgttatgttctataatattta acagttatttgaaattctattctttaatta aataaattaa(AT)7	117	36553	36669
p1	(A)11	11	39247	39257
c	(AT)6c(T)10	23	41087	41109
p1	(T)11	11	41281	41291
p2	(AT)6	12	47212	47223
c	(A)12(TA)6	24	51869	51892
p4	(TATT)5	20	52951	52970
p1	(T)10	10	53086	53095
p1	(A)12	12	53303	53314
p1	(A)15	15	54890	54904
p1	(A)10	10	56816	56825
p1	(A)11	11	59633	59643

p1	(T)10	10	59749	59758
p1	(T)10	10	64450	64459
p1	(T)10	10	64876	64885
p1	(T)10	10	69497	69506
p1	(A)16	16	70182	70197
p2	(TA)6	12	72601	72612
p1	(A)10	10	74477	74486
p1	(G)11	11	77412	77422
p1	(T)10	10	78793	78802
p1	(A)10	10	80870	80879
p1	(T)11	11	81155	81165
p1	(T)10	10	82911	82920
p1	(A)10	10	106094	106103
p1	(T)11	11	110687	110697
p1	(A)11	11	115075	115085
p1	(T)15	15	121396	121410
p1	(T)10	10	127808	127817
p1	(A)10	10	150991	151000

Table S4 Microsatellite repeats in the *A. giraldii* mitogenome. The structure of Microsatellite repeats is presented as repeat units surrounded by parenthesis, follow with the number of the repeat unit in the repeat sequence.

Type	Structure of Microsatellite Repeats	Size (bp)	Start	End
p4	(GCCC)3	12	2906	2917
p4	(TTAA)4	16	9791	9806
p4	(AAGA)3	12	10787	10798
p4	(GGCG)3	12	21332	21343
p4	(AATC)3	12	22939	22950
p1	(T)11	11	23040	23050
p4	(TTCA)3	12	29626	29637
p4	(GGAA)3	12	30077	30088
p4	(CTTG)3	12	39743	39754
p1	(A)10	10	42133	42142
p1	(A)10	10	42655	42664
p4	(TGAA)3	12	46740	46751
p1	(A)10	10	46830	46839
p1	(T)10	10	58008	58017
p4	(GAAA)3	12	59513	59524
p2	(CT)5	10	63616	63625
p2	(AG)5	10	65488	65497
p2	(TA)6	12	66320	66331
p3	(CTT)4	12	72232	72243
p1	(C)10	10	78253	78262
p4	(CATT)3	12	78926	78937
p2	(TC)5	10	81420	81429
p2	(AG)5	10	95406	95415
p1	(G)10	10	96053	96062

p2	(CT)5	10	97628	97637
p2	(TA)5	10	106136	106145
p1	(T)10	10	106459	106468
p4	(TTGG)3	12	107161	107172
p4	(GCCG)3	12	112369	112380
p2	(AG)5	10	115573	115582
p3	(GAA)4	12	122262	122273
p1	(A)10	10	125449	125458
p1	(C)11	11	127199	127209
p5	(ATATT)3	15	131076	131090
p4	(AATC)3	12	133519	133530
p4	(AAGC)3	12	136422	136433
p4	(GGGC)3	12	139392	139403
p4	(TTAC)3	12	142562	142573
p1	(A)11	11	144927	144937
p4	(GACC)3	12	148201	148212
p2	(TA)5	10	153452	153461
p1	(T)11	11	156651	156661
p2	(GA)6	12	158878	158889
p4	(ATAA)3	12	161399	161410
p4	(TTCG)3	12	166010	166021
p1	(T)10	10	168365	168374
p5	(CTAGT)3	15	171351	171365
p1	(A)11	11	177186	177196
p4	(ACCG)3	12	190270	190281
p4	(TCAT)3	12	191292	191303
p1	(A)10	10	191532	191541

Table S5 Tandem repeats in the *A. giraldii* plastome.

Start and End Sites of the Tandem Repeats	Period Size (bp)	Copy Number	Consensus Size (bp)	Percent Matches	Indels	Score	Bases number				Extropy (0-2)
							A	G	C	T	
8704-8769	13	5.1	13	74	17	57	12	6	4	77	1.1
8735-8774	15	2.7	15	100	0	80	7	5	5	82	0.94
16032-16072	21	2	21	95	0	73	34	9	24	31	1.88
17483-17518	17	2.1	17	100	0	72	16	19	5	58	1.58
26578-26627	24	2.1	24	96	0	91	32	18	14	36	1.9
31080-31114	15	2.3	15	90	0	52	37	14	14	34	1.86
35144-35179	15	2.4	15	86	13	56	19	0	19	61	1.35
36984-37017	17	2	17	100	0	68	11	17	0	70	1.16
41048-41092	9	5.1	9	78	5	56	40	0	11	48	1.39
41044-41096	26	2	26	100	0	106	37	0	11	50	1.38
52716-52758	22	2	22	90	4	70	18	6	25	48	1.73
57784-57808	12	2.1	12	100	0	50	24	8	8	60	1.52
77782-77829	15	3.3	15	85	2	71	25	10	12	52	1.71
90067-90144	18	4.3	18	95	0	129	29	10	26	33	1.89
90067-90144	9	8.7	9	68	0	57	29	10	26	33	1.89
97723-97747	10	2.5	10	100	0	50	40	8	0	52	1.31
105734-105795	32	1.9	32	96	0	115	41	20	8	29	1.81
110290-110340	25	2	25	100	0	102	60	3	19	15	1.5
111720-111745	11	2.4	11	100	0	52	53	11	7	26	1.63
128116-128177	32	1.9	32	96	0	115	29	8	20	41	1.81
136164-136188	10	2.5	10	100	0	50	52	0	8	40	1.31
143767-143844	18	4.3	18	95	0	129	33	26	10	29	1.89
143780-143843	9	7.1	9	70	0	56	34	28	7	29	1.85

Table S6 Tandem repeats in the *A. giraldii* mitogenome.

Start and End Sites of the Tandem Repeats	Period Size (bp)	Copy Number	Consensus Size (bp)	Percent Matches	Indels	Score	Bases number				Extropy (0-2)
							A	G	C	T	
3469-3524	28	2	28	100	0	112	21	7	21	50	1.72
16425-16475	25	2	25	100	0	102	49	27	19	3	1.66
24778-24863	21	4.2	21	76	11	97	10	10	25	53	1.67
24785-24875	21	4.6	21	78	10	77	12	12	24	51	1.72
32401-32450	25	2	25	100	0	100	24	24	16	36	1.94
34026-34069	21	2.1	21	91	0	70	38	20	13	27	1.9
35680-35722	18	2.4	18	81	18	54	34	20	25	18	1.96
84423-84460	19	2	19	100	0	76	52	21	10	15	1.72
115241-115274	18	1.9	17	88	5	50	35	20	29	14	1.93
127724-127832	36	3	36	100	0	218	8	42	30	19	1.8
127728-127822	18	5.3	18	64	26	58	9	43	29	17	1.81
137363-137403	20	2	20	86	9	57	53	21	2	21	1.57
177435-177474	16	2.6	16	88	4	55	5	27	30	37	1.78
184442-184520	39	2	39	95	0	140	22	17	27	31	1.97
192089-192139	25	2	25	100	0	102	49	27	19	3	1.66

Table S7 Dispersed repeats in the *A. giraldii* plastome.

The repeat length of the first part	The starting site of the first part	Matching direction	The repeat length of the second part	The starting site of the second part	interval distance of repeats	E-value
60	90066	F	60	90084	-3	4.46E-21
60	90066	P	60	143766	-3	4.46E-21
60	90084	P	60	143784	-3	4.46E-21
60	143766	F	60	143784	-3	4.46E-21
48	9793	P	48	9793	0	8.10E-20
45	90081	F	45	90099	0	5.19E-18
45	90081	P	45	143766	0	5.19E-18
45	90099	P	45	143784	0	5.19E-18
39	96842	F	39	118145	0	2.12E-14
39	118145	P	39	137029	0	2.12E-14
41	39812	P	41	96840	-1	1.63E-13
41	39812	F	41	137029	-1	1.63E-13
39	39812	P	39	118145	-1	2.48E-12
43	115025	P	43	115025	-3	2.76E-11
42	90066	F	42	90102	-3	1.03E-10
42	90066	P	42	143766	-3	1.03E-10
42	90102	P	42	143802	-3	1.03E-10
42	143766	F	42	143802	-3	1.03E-10
33	110282	F	33	110307	-2	4.13E-07
30	38132	P	30	74369	-1	5.01E-07
30	105733	F	30	105765	-1	5.01E-07
30	105733	P	30	128115	-1	5.01E-07
30	105765	P	30	128147	-1	5.01E-07

30	128115	F	30	128147	-1	5.01E-07
35	39813	P	35	93791	-3	9.61E-07
35	39813	F	35	140084	-3	9.61E-07
32	41043	F	32	41069	-2	1.55E-06
32	42625	F	32	44849	-2	1.55E-06
30	16533	P	30	98003	-2	2.18E-05
30	16533	F	30	135877	-2	2.18E-05
30	38132	P	30	48053	-2	2.18E-05
30	90079	F	30	90115	-2	2.18E-05
30	90079	P	30	143765	-2	2.18E-05
30	90115	P	30	143801	-2	2.18E-05
31	48053	F	31	74369	-3	1.69E-04
30	13551	R	30	53284	-3	6.10E-04
30	26573	F	30	26597	-3	6.10E-04
60	90066	F	60	90084	-3	4.46E-21

F: forward repeats; P: palindromic repeats; R: reverse repeats

Table S8 Dispersed repeats in the *A. giraldii* mitogenome.

The repeat length of the first part	The starting site of the first part	Matching direction	The repeat length of the second part	The starting site of the second part	interval distance of repeats	E-value
248	16305	F	248	191969	0	5.19E-140
228	108425	F	228	138184	0	5.71E-128
214	71057	F	214	156336	-2	3.14E-114
195	74926	F	195	162478	-3	1.38E-100
169	74952	F	169	162504	-2	2.42E-87
159	156384	F	159	194139	0	1.99E-86
159	71105	F	159	194139	-2	2.25E-81
146	130162	F	146	180576	0	1.33E-78
142	201	F	142	117799	0	3.42E-76
153	190	F	153	117788	-3	1.29E-75
113	19346	F	113	76441	0	9.85E-59
112	0	F	112	156438	-1	1.32E-55
107	70089	F	107	117630	0	4.03E-55
114	74749	F	114	162304	-2	1.43E-54
105	0	F	105	194193	0	6.45E-54
115	0	F	115	71159	-3	4.10E-53
101	7024	P	101	134628	0	1.65E-51
98	60341	F	98	162213	0	1.06E-49
79	8811	P	79	118080	0	2.91E-38
73	127723	F	73	127759	0	1.19E-34
77	127357	F	77	127950	-2	1.22E-32
76	53659	P	76	87807	-3	3.53E-30
64	6584	F	64	87822	0	3.12E-29

56	129616	P	56	134727	0	2.04E-24
58	6587	P	58	53659	-1	2.22E-23
52	87753	P	52	173437	0	5.23E-22
61	84906	P	61	106970	-3	1.94E-21
60	15590	P	60	141219	-3	7.38E-21
53	137912	F	53	193874	-1	2.08E-20
49	71059	P	49	99877	0	3.35E-20
49	87131	P	49	141397	0	3.35E-20
49	99877	P	49	156338	0	3.35E-20
49	124198	P	49	139994	0	3.35E-20
53	11537	F	53	193915	-2	1.62E-18
55	15641	P	55	76879	-3	5.79E-18
44	122669	F	44	166968	0	3.43E-17
50	54158	P	50	162755	-2	9.23E-17
50	60651	F	50	107058	-2	9.23E-17
43	21822	F	43	138548	0	1.37E-16
46	60462	P	46	69345	-1	2.96E-16
46	65604	P	46	126316	-1	2.96E-16
52	86872	P	52	168011	-3	3.12E-16
52	86886	P	52	167997	-3	3.12E-16
42	91259	F	42	155667	0	5.49E-16
42	127322	F	42	127916	0	5.49E-16
40	137925	F	40	193887	0	8.78E-15
39	71022	F	39	156302	0	3.51E-14
39	121996	P	39	149142	0	3.51E-14
38	18254	F	38	192536	0	1.41E-13
38	125589	P	38	128188	0	1.41E-13
41	11549	F	41	193927	-1	2.70E-13

41	49921	P	41	191603	-1	2.70E-13
44	193091	F	44	193169	-2	2.92E-13
37	127723	F	37	127795	0	5.62E-13
46	70540	F	46	173893	-3	8.79E-13
39	15611	P	39	141219	-1	4.11E-12
35	60666	F	35	107073	0	8.99E-12
34	54174	P	34	162755	0	3.60E-11
34	97628	P	34	97628	0	3.60E-11
43	70549	F	43	173902	-3	4.57E-11
43	77584	F	43	110058	-3	4.57E-11
43	109267	F	43	172436	-3	4.57E-11
40	29065	F	40	47210	-2	6.17E-11
40	184441	F	40	184480	-2	6.17E-11
37	59372	P	37	177152	-1	6.24E-11
33	7179	F	33	121937	0	1.44E-10
42	97994	F	42	172018	-3	1.70E-10
35	76580	P	35	126457	-1	9.44E-10
37	86901	P	37	167997	-2	3.37E-09
34	193101	F	34	193179	-1	3.67E-09
39	38291	P	39	108634	-3	8.67E-09
39	74833	F	39	162388	-3	8.67E-09
30	9246	F	30	166894	0	9.21E-09
30	21262	F	30	88485	0	9.21E-09
30	65620	P	30	126316	0	9.21E-09
30	89376	P	30	177974	0	9.21E-09
30	102679	P	30	104420	0	9.21E-09
33	21586	P	33	167293	-1	1.42E-08
33	31545	P	33	39918	-1	1.42E-08

38	108990	F	38	181984	-3	3.20E-08
38	126692	F	38	162301	-3	3.20E-08
35	192894	F	35	193022	-2	4.82E-08
37	52898	F	37	59455	-3	1.18E-07
37	74873	F	37	162428	-3	1.18E-07
34	74750	F	34	126696	-2	1.82E-07
34	77612	F	34	110086	-2	1.82E-07
31	13372	F	31	24498	-1	2.14E-07
31	77995	P	31	171748	-1	2.14E-07
36	70530	F	36	173883	-3	4.33E-07
36	74891	F	36	162446	-3	4.33E-07
30	20618	F	30	47696	-1	8.29E-07
35	74855	F	35	162410	-3	1.59E-06
35	94338	F	35	124518	-3	1.59E-06
32	77618	F	32	149393	-2	2.57E-06
34	3316	F	34	5148	-3	5.81E-06
34	60718	P	34	107166	-3	5.81E-06
34	167290	P	34	168062	-3	5.81E-06
31	34256	P	31	67371	-2	9.64E-06
31	38254	P	31	108698	-2	9.64E-06
31	44829	P	31	150179	-2	9.64E-06
31	74865	F	31	162420	-2	9.64E-06
31	157505	P	31	178766	-2	9.64E-06
31	183736	R	31	183736	-2	9.64E-06
33	20644	P	33	123608	-3	2.12E-05
33	75166	F	33	162718	-3	2.12E-05
33	75182	F	33	162734	-3	2.12E-05
30	3468	F	30	3496	-2	3.61E-05

30	9052	P	30	173602	-2	3.61E-05
30	16424	F	30	16449	-2	3.61E-05
30	16424	F	30	192113	-2	3.61E-05
30	16449	F	30	192088	-2	3.61E-05
30	40252	F	30	172404	-2	3.61E-05
30	109047	F	30	182041	-2	3.61E-05
30	109280	F	30	172449	-2	3.61E-05
30	125697	P	30	171498	-2	3.61E-05
30	137042	F	30	159404	-2	3.61E-05
30	192088	F	30	192113	-2	3.61E-05
32	70275	P	32	129568	-3	7.71E-05
32	122332	F	32	122389	-3	7.71E-05
32	193042	F	32	193132	-3	7.71E-05
31	95	F	31	71254	-3	2.79E-04
31	21263	F	31	136545	-3	2.79E-04
31	52906	F	31	59463	-3	2.79E-04
31	70567	F	31	173920	-3	2.79E-04
31	110089	F	31	149390	-3	2.79E-04
31	123137	F	31	172818	-3	2.79E-04
30	9574	F	30	42290	-3	1.01E-03
30	21545	P	30	36826	-3	1.01E-03
30	24806	F	30	24827	-3	1.01E-03
30	46560	P	30	59447	-3	1.01E-03

F: forward repeats; P: palindromic repeats; R: reverse repeats

Table S9 Homologous fragments between the *A. giraldii* organelle genomes.

ID	Length(bp)	mtDNA		mt_region	cpDNA		cp_region	E-value	Identities
		Start	End		Start	End			
F1	888	19604	20462	partial(*rrn*18)	99147<br>134764	100010<br>133901	partial(*rrn*16S)<br>partial(*rrn*16S)	1.19E-80	73.761
F2	808	57981	58769	*trn*W-CCA	18363	17583	*trn*W-CCA, *pet*G, *pet*L	1.80E-148	79.332
F3	411	40570	40980	IGS(*rrn*26-*nad*2)	44058	43648	partial(*psb*A, *psb*B)	0	99.513
F4	398	116775	117152	nad5	129127<br>104784	129520<br>104391	partial(*rrn*23S)<br>partial(*rrn*23S)	8.81E-112	85.930
F5	256	15259	15505	IGS(*trn*H-GUG-*rrn*18)	143459<br>90452	143204<br>90707	partial(*ycf*2)<br>partial(*ycf*2)	4.16E-100	92.969
F6	176	58863	59038	*trn*P-UGG	17459	17284	*trn*P-UGG	9.52E-57	89.266
F7	84	47182	47265	*trn*N-GUU	106837<br>127074	106919<br>126992	*trn*N-GUU<br>*trn*N-GUU	9.86E-32	96.429
F8	80	11374	11453	*trn*H-GUG	82753	82832	*trn*H-GUG	9.86E-32	97.500
F9	79	131786	131864	*trn*M-CAU	30978	30900	*trn*M-CAU	3.54E-31	97.468

cp: plastid; mt: mitochondria

Table S10 Benjamini-Hochberg correction for multiple testing of 28 protein-coding genes.

Gene	p-value	Rank	(I/M)Q
*ccm*Fc	0.000000000	1	0
*nad*1	0.000000000	2	0
*nad*6	0.000000025	3	2.33333E-07
*atp*9	0.000002213	4	0.000015491
*atp*1	0.000004432	5	2.48192E-05
*rps*12	0.000044192	6	0.000206229
*nad*9	0.014740124	7	0.058960496
*nad*7	0.037335562	8	0.130674467
*nad*4	0.089072289	9	0.277113788
*mat*R	0.215648179	10	0.603814901
*cyt*b	0.330115066	12	0.770268487
*cox*2	0.329861635	11	0.839647798
*ccm*B	0.999983000	28	0.999983
*rps*13	0.999980000	27	1.037016296
*nad*4L	0.999941002	26	1.076859541
*nad*3	0.999881007	25	1.119866728
*nad*2	0.999831014	24	1.166469516
*ccm*Fn	0.647920031	15	1.209450725
*atp*8	0.999761029	23	1.217100383
*ccm*C	0.999740034	22	1.272396407
*cox*1	0.647068576	14	1.294137152
*mtt*B	0.999706043	21	1.332941391
*rpl*10	0.635590367	13	1.368963867
*rpl*5	0.999640065	20	1.399496091
*nad*5	0.999222303	19	1.472538131
*cox*3	0.999030470	18	1.554047398
*rps*4	0.945699891	17	1.55762335

i: the individual p-value's rank; m: total number of tests; p: p-value

Table S11 The likelihood ratio test (LRT) p-value based on the lnL and np values of the null model and the alternative model for 28 protein-coding genes in the mitogenome. The Bayes Empirical Bayes (BEB) analysis under model M8 is used to identify codons under positive selection. The column "Positive sites" lists the amino acids with BEB scores higher than 0.5. The sites with BEB values higher than 0.95 are indicated with an asterisk and the sites with BEB values higher than 0.99 are characterized by two asterisks. In the column "Positive sites", three characters are a group, the first number represents the position of the codon, the second letter represents the amino acid encoded by the codon, and the third number represents the BEB value.

Site model (SM)										
Gene name	Model	np	Ln L	Estimates of parameters				Model compared	LRT P-value	Positive sites
*atp*1	M3	23	-2204.388915	p:	0.00000	0.98566	0.01434			[]
				ω:	0.00000	0.03453	39.19239			
	M0	19	-2222.958395	ω0:	0.35583			M0 vs. M3	0.000000169	Not Allowed
	M2a	22	-2204.389250	p:	0.98565	0.00000	0.01435			[]
				ω:	0.03453	1.00000	39.19375			
	M1a	20	-2216.716010	p:	0.84046	0.15954		M1a vs. M2a	0.000004432	Not Allowed
				ω:	0.00000	1.00000				
	M8	22	-2204.421663	p0=0.98272	p=0.00500	q=2.97258		M7 vs.M8	0.000001148	105 M 0.693, 200 D 0.701 ,248 L 0.995**, 355 S 0.983*, 446 L 0.992**, 469 R 0.715, 507 P 0.978*
				(p1= 0.01728)	ω= 33.86456					

	M7	20	-2218.098875	p=	0.01832	q=	0.04085			Not Allowed
	M8a	21	-2216.716731	p0=0.84046	p=0.00500	q=3.91034		M8a vs.M8	0.000000709	Not Allowed
				(p1= 0.15954)	ω= 1.00000					
*atp*6	M3	23	-1080.100437	p:	0.78503	0.15885	0.05612			[]
				ω:	0.00000	0.00000	1.63380			
	M0	19	-1082.241002	ω0:	0.08388			M0 vs. M3	0.369293972	Not Allowed
	M2a	22	-1080.100437	p:	0.94388	0.00000	0.05612			[]
				ω:	0.00000	1.00000	1.63378			
	M1a	20	-1080.203400	p:	0.91902	0.08098		M1a vs. M2a	0.902160353	Not Allowed
				ω:	0.00000	1.00000				
	M8	22	-1080.100432	p0=0.94387	p=0.00500	q=1.71695				114 I 0.778
				(p1= 0.05613)	ω= 1.63377					
	M7	20	-1081.208554	p=	0.04107	q=	0.25518	M7 vs.M8	0.330178454	Not Allowed
	M8a	21	-1080.203391	p0=0.91902	p=0.00820	q=10.41206		M8a vs.M8	0.649985822	Not Allowed
				(p1= 0.08098)	ω= 1.00000					
*atp*8	M3	23	-682.042031	p:	0.98157	0.00509	0.01335			[]
				ω:	0.17637	0.17643	0.17644			
	M0	19	-682.041726	ω0:	0.17637			M0 vs. M3	0.999999953	Not Allowed
	M2a	22	-682.041992	p:	1.00000	0.00000	0.00000			[]
				ω:	0.17637	1.00000	1.00000			
	M1a	20	-682.041753	p:	0.99999	0.00001		M1a vs. M2a	0.999761029	Not Allowed
				ω:	0.17637	1.00000				
	M8	22	-682.042505	p0=0.99999	p=21.31681	q=99.00000		M7 vs.M8	0.999951001	
				(p1= 0.00001)	ω= 1.00000					

	M7	20	-682.042456	p=	21.31764	q=	99.00000			Not Allowed
				p0=0.99999	p=21.31669	q=99.00000				
	M8a	21	-682.042424	(p1= 0.00001)	ω= 1.00000			M8a vs.M8	0.989844862	Not Allowed
*atp*9				p:	0.97642	0.00000	0.02358			
	M3	23	-389.655775	ω:	0.00000	0.00000	142.13028			[]
	M0	19	-408.733672	ω0:	0.55238			M0 vs. M3	0.000000104	Not Allowed
				p:	0.97642	0.00000	0.02358			
	M2a	22	-389.655775	ω:	0.00000	1.00000	142.12993			[]
				p:	0.89466	0.10534				
	M1a	20	-402.676744	ω:	0.00000	1.00000		M1a vs. M2a	0.000002213	Not Allowed
				p0=0.97642	p=0.00500	q=3.01274				
	M8	22	-389.655767	(p1= 0.02358)	ω= 142.12916					84 S 1.000**, 85 T 0.967*
	M7	20	-404.168289	p=	0.00500	q=	0.00564	M7 vs.M8	0.000000498	Not Allowed
				p0=0.89467	p=0.00500	q=2.24345				
	M8a	21	-402.676724	(p1= 0.10533)	ω= 1.00000			M8a vs.M8	0.000000334	Not Allowed
*ccm*B				p:	0.04996	0.00297	0.94706			
	M3	23	-843.514835	ω:	0.00000	0.00000	0.00000			[]
	M0	19	-843.515914	ω0:	0.00010			M0 vs. M3	0.999999418	Not Allowed
				p:	1.00000	0.00000	0.00000			
	M2a	22	-843.514873	ω:	0.00000	1.00000	1.00000			[]
				p:	0.99999	0.00001				
	M1a	20	-843.514856	ω:	0.00000	1.00000		M1a vs. M2a	0.999983	Not Allowed
	M8	22	-843.515086	p0=0.99999	p=0.00500	q=1.76962		M7 vs.M8	0.999905005	

				(p1= 0.00001)	ω= 1.00000					
	M7	20	-843.514991	p=	0.00715	q=	1.63528			Not Allowed
				p0=0.99999	p=0.00500	q=1.89715				
	M8a	21	-843.515398	(p1= 0.00001)	ω= 1.00000			M8a vs.M8	0.980070923	Not Allowed
*ccm*C				p:	0.08316	0.77808	0.13876			
	M3	23	-1037.798921	ω:	0.80458	0.80459	0.80460			[]
	M0	19	-1037.798996	ω0:	0.80459			M0 vs. M3	0.999999997	Not Allowed
				p:	1.00000	0.00000	0.00000			
	M2a	22	-1037.799271	ω:	0.80459	1.00000	1.00000			[]
				p:	0.99999	0.00001				
	M1a	20	-1037.799011	ω:	0.80459	1.00000		M1a vs. M2a	0.999740034	Not Allowed
				p0=0.99999	p=99.00000	q=24.11942				4 S 0.562,<br>9 Y 0.552,<br>20 N 0.561,<br>21 F 0.546,<br>94 T 0.556,<br>141 R 0.557,<br>207 R 0.557
	M8	22	-1037.799517	(p1= 0.00001)	ω= 1.00000					
	M7	20	-1037.799291	p=	99.00000	q=	24.10835	M7 vs.M8	0.999774026	Not Allowed
				p0=0.99999	p=99.00000	q=24.10937				
	M8a	21	-1037.799406	(p1= 0.00001)	ω= 1.00000			M8a vs.M8	0.988112228	Not Allowed
*ccm*Fc				p:	0.00015	0.88302	0.11683			
	M3	23	-1723.588965	ω:	0.56559	0.56685	20.21258			[]
	M0	19	-1758.024175	ω0:	1.19240			M0 vs. M3	0.000000000	Not Allowed

				p:	0.88311	0.00006	0.11683			
	M2a	22	-1723.588975	ω:	0.56682	1.00000	20.21277			[]
				p:	0.46633	0.53367				
	M1a	20	-1750.851004	ω:	0.00000	1.00000		M1a vs. M2a	0	Not Allowed
				p0=0.75123	p=0.00500	q=0.94343				35 P 0.577,
	M8	22	-1727.612030	(p1= 0.24877)	ω= 9.03854			M7 vs.M8	0.000000000	53 V 0.555,<br>76 L 0.570,<br>81 Q 0.594,<br>91 R 0.560,<br>136 S 0.948,<br>161 R 0.656,<br>175 C 0.579,<br>179 P 0.565,<br>185 V 0.554,<br>198 S 0.965*,<br>201 G 0.553,<br>209 P 0.558,<br>256 K 0.997**,<br>257 I 0.988*,<br>258 Q 0.668,<br>259 F 0.987*,<br>261 Q 0.944,<br>262 R 0.975*,<br>263 L 0.899,<br>265 L 0.618,

										266 G 0.587,<br>267 S 0.963*,<br>268 E 0.881,<br>269 L 0.948,<br>272 G 0.993**,<br>273 K 0.582,<br>274 E 0.633,<br>275 R 0.589,<br>276 C 0.594,<br>277 C 0.989*,<br>278 L 0.997**,<br>279 Q 0.993**,<br>281 L 0.999**,<br>282 D 0.948,<br>283 Y 0.989*,<br>284 L 0.601,<br>285 H 0.952*,<br>286 G 0.991**,<br>289 F 0.995**,<br>290 H 0.940,<br>291 S 0.992**,<br>292 I 0.556,<br>293 C 0.540
	M7	20	-1751.818026	p=	0.01619	q=	0.01045			Not Allowed
	M8a	21	-1750.850988	p0=0.46633	p=0.00500	q=1.29329		M8a vs.M8	0.000000000	Not Allowed

				(p1= 0.53367)	ω= 1.00000					
				p:	0.73628	0.20597	0.05776			
	M3	23	-2404.808187	ω:	0.00000	3.11036	3.11036			[]
	M0	19	-2405.463947	ω0:	0.80128			M0 vs. M3	0.859417984	Not Allowed
				p:	0.73627	0.00000	0.26373			
	M2a	22	-2404.807763	ω:	0.00000	1.00000	3.11029			[]
				p:	0.27819	0.72181				
	M1a	20	-2405.241751	ω:	0.00000	1.00000		M1a vs. M2a		Not Allowed
				p0=0.73628	p=0.00500	q=1.37881				15 S 0.519,<br>65 N 0.503,<br>151 F 0.510,<br>234 S 0.521,<br>245 D 0.517,<br>287 G 0.522,<br>293 I 0.515,<br>294 R 0.518,<br>302 F 0.510,<br>305 V 0.515,<br>336 A 0.516,<br>409 R 0.523,
*ccm*Fn	M8	22	-2404.806467	(p1= 0.26372)	ω= 3.11041					461 S 0.801
	M7	20	-2405.261304	p=	0.01005	q=	0.00500	M7 vs.M8	0.634551391	Not Allowed
				p0=0.27819	p=0.00920	q=2.00580				
	M8a	21	-2405.241826	(p1= 0.72181)	ω= 1.00000			M8a vs.M8	0.350756613	Not Allowed
*cox*1	M3	23	-2404.806691	p:	0.73627	0.04559	0.21814	M0 vs. M3	0.868716543	[]

				ω:	0.00000	3.11010	3.11030			
	M0	19	-2405.434926	ω0:	0.81245					Not Allowed
	M2a	22	-2404.806684	p:	0.73628	0.00000	0.26372			[]
				ω:	0.00000	1.00000	3.11050			
	M1a	20	-2405.241987	p:	0.27819	0.72181		M1a vs. M2a		Not Allowed
				ω:	0.00000	1.00000				
	M8	22	-2404.806589	p0=0.73628	p=0.00500	q=1.97764				15 S 0.519, 65 N 0.503, 151 F 0.510, 234 S 0.521, 245 D 0.517, 287 G 0.522, 293 I 0.515, 294 R 0.518, 302 F 0.510, 305 V 0.515, 336 A 0.516, 409 R 0.523, 461 S 0.801
				(p1= 0.26372)	ω= 3.11042					
	M7	20	-2405.548515	p=	0.31497	q=	0.00500	M7 vs.M8	0.476195878	Not Allowed
	M8a	21	-2405.242658	p0=0.27815	p=0.00978	q=1.69006		M8a vs.M8	0.350364099	Not Allowed
				(p1= 0.72185)	ω= 1.00000					
*cox*2	M3	23	-778.275153	p:	0.72065	0.20352	0.07583			[]
				ω:	0.00000	2.71728	2.71731			
	M0	19	-780.917407	ω0:	0.63226			M0 vs. M3	0.259330679	Not Allowed

				p:	0.72065	0.00000	0.27935			
	M2a	22	-778.275153	ω:	0.00000	1.00000	2.71728			[]
				p:	0.49329	0.50671				
	M1a	20	-779.384235	ω:	0.00000	1.00000		M1a vs. M2a		Not Allowed
				p0=0.72065	p=0.00500	q=1.28947				128 T 0.732,
	M8	22	-778.275153	(p1= 0.27935)	ω= 2.71728					130 E 0.718, 141 G 0.815, 142 Q 0.744
	M7	20	-779.759885	p=	0.01611	q=	0.01421	M7 vs.M8	0.226563051	Not Allowed
				p0=0.49329	p=0.00807	q=1.54798				
	M8a	21	-779.384233	(p1= 0.50671)	ω= 1.00000			M8a vs.M8	0.136395534	Not Allowed
				p:	0.00000	0.00000	1.00000			
	M3	23	-1103.470972	ω:	0.00000	0.00000	0.59342			[]
	M0	19	-1103.471251	ω0:	0.59341			M0 vs. M3	0.999999961	Not Allowed
				p:	1.00000	0.00000	0.00000			
	M2a	22	-1103.470922	ω:	0.59341	1.00000	1.00000			[]
				p:	0.99999	0.00001				
*cox*3	M1a	20	-1103.471892	ω:	0.59340	1.00000		M1a vs. M2a		Not Allowed
				p0=0.98954	p=99.00000	q=70.74347				100 L 0.548,
	M8	22	-1103.471490	(p1= 0.01046)	ω= 1.00000					189 S 0.560, 220 I 0.553
	M7	20	-1103.471328	p=	99.00000	q=	68.53951	M7 vs.M8	0.999838013	Not Allowed
				p0=0.99999	p=99.00000	q=67.86155				
	M8a	21	-1103.471150	(p1= 0.00001)	ω= 1.00000			M8a vs.M8	0.979196074	Not Allowed

*cyt*b	M3	23	-1657.687509	p:	0.75920	0.14329	0.09751			
				ω:	0.00000	0.00000	3.71338			[]
	M0	19	-1660.833589	ω0:	0.34442			M0 vs. M3	0.178366173	Not Allowed
	M2a	22	-1657.687292	p:	0.90249	0.00000	0.09751			
				ω:	0.00000	1.00000	3.71333			[]
	M1a	20	-1658.795606	p:	0.72628	0.27372				
				ω:	0.00000	1.00000		M1a vs. M2a		Not Allowed
	M8	22	-1657.687148	p0=0.90249	p=0.01015	q=1.84469				82 Y 0.566,<br>104 L 0.890,<br>109 L 0.531,<br>252 P 0.568,<br>345 F 0.864,<br>372 C 0.569
				(p1= 0.09751)	ω= 3.71329					
	M7	20	-1658.919204	p=	0.00817	q=	0.01285	M7 vs.M8	0.291692242	Not Allowed
	M8a	21	-1658.795397	p0=0.72629	p=0.00500	q=2.10762				
				(p1= 0.27371)	ω= 1.00000			M8a vs.M8	0.136542473	Not Allowed
*mat*R	M3	23	-2917.448570	p:	0.82591	0.03950	0.13459			
				ω:	0.00000	0.00000	5.02313			[]
	M0	19	-2919.873463	ω0:	0.66547			M0 vs. M3	0.303060516	Not Allowed
	M2a	22	-2917.448083	p:	0.86542	0.00000	0.13458			
				ω:	0.00000	1.00000	5.02310			[]
	M1a	20	-2918.982190	p:	0.47106	0.52894				
				ω:	0.00000	1.00000		M1a vs. M2a		Not Allowed
	M8	22	-2917.448959	p0=0.86539	p=0.00500	q=1.72565		M7 vs.M8	0.177836801	91 G 0.504,

				(p1= 0.13461)	ω= 5.02255					193 T 0.506,<br>211 S 0.859,<br>235 G 0.503,<br>458 A 0.853,<br>470 V 0.502,<br>494 A 0.505,<br>651 T 0.519,<br>654 L 0.514
	M7	20	-2919.175848	p=	0.00785	q=	0.00730			Not Allowed
				p0=0.47108	p=0.00500	q=1.30081				
	M8a	21	-2918.981488	(p1= 0.52892)	ω= 1.00000			M8a vs.M8	0.079992309	Not Allowed
				p:	0.00000	0.00006	0.99994			
	M3	23	-1034.668275	ω:	0.23662	0.39987	0.40185			[]
	M0	19	-1034.668179	ω0:	0.40187			M0 vs. M3	0.999999995	Not Allowed
				p:	1.00000	0.00000	0.00000			
	M2a	22	-1034.668179	ω:	0.40187	1.00000	1.00000			[]
				p:	0.99999	0.00001				
*mtt*B	M1a	20	-1034.668473	ω:	0.40186	1.00000		M1a vs. M2a		Not Allowed
				p0=0.99999	p=67.21070	q=99.00000				
	M8	22	-1034.668390	(p1= 0.00001)	ω= 1.00000					
	M7	20	-1034.668405	p=	66.56814	q=	99.00000	M7 vs.M8	0.999985000	Not Allowed
				p0=0.99999	p=67.41959	q=99.00000				
	M8a	21	-1034.668388	(p1= 0.00001)	ω= 1.00000			M8a vs.M8	0.998404232	Not Allowed
*nad*1	M3	23	-1266.367764	p:	0.00096	0.90780	0.09124	M0 vs. M3	0.000000000	[]

				ω:	0.00000	0.07876	20.28643			
	M0	19	-1300.104932	ω0:	0.69009					Not Allowed
				p:	0.81370	0.09898	0.08732			
	M2a	22	-1266.321911	ω:	0.00000	1.00000	20.90455			[]
				p:	0.69722	0.30278				
	M1a	20	-1289.122462	ω:	0.00000	1.00000		M1a vs. M2a	0.000000000	Not Allowed
				p0=0.89313	p=0.00500	q=3.97765				128 S 0.994**,<br>129 N 0.752,<br>146 P 0.981*,<br>212 S 0.795,<br>221 R 0.998**,<br>222 D 0.685,<br>223 A 0.845,<br>224 I 0.853,<br>225 N 0.983*,<br>226 S 0.985*,<br>228 L 0.833,<br>230 A 0.835,<br>234 P 1.000**,<br>235 S 0.999**,<br>237 L 0.707,<br>238 P 0.848,<br>239 I 0.981*,<br>243 I 0.999**,
	M8	22	-1266.541109	(p1= 0.10687)	ω= 17.09086			M7 vs.M8	0.000000000	244 P 0.861,

										245 G 0.880,<br>246 S 0.962*,<br>247 I 0.854,<br>248 R 0.999**
	M7	20	-1289.199373	p=	0.01076	q=	0.01732			Not Allowed
				p0=0.69722	p=0.00820	q=1.45152				
	M8a	21	-1289.122434	(p1= 0.30278)	ω= 1.00000			M8a vs.M8	0.000000000	Not Allowed
				p:	0.05409	0.78297	0.16294			
	M3	23	-1263.143582	ω:	0.26468	0.26469	0.26470			[]
	M0	19	-1263.143229	ω0:	0.26469			M0 vs. M3	0.999999938	Not Allowed
				p:	1.00000	0.00000	0.00000			
	M2a	22	-1263.143301	ω:	0.26469	1.00000	1.00000			[]
				p:	0.99999	0.00001				
*nad*2	M1a	20	-1263.143470	ω:	0.26468	1.00000		M1a vs. M2a	0.999831014	Not Allowed
				p0=0.99999	p=13.57880	q=37.48553				
	M8	22	-1263.144773	(p1= 0.00001)	ω= 15.66202					
	M7	20	-1263.143423	p=	35.72878	q=	99.00000	M7 vs.M8	0.998650911	Not Allowed
				p0=0.99999	p=35.72455	q=99.00000				
	M8a	21	-1263.143753	(p1= 0.00001)	ω= 1.00000			M8a vs.M8	0.963974708	Not Allowed
				p:	0.81868	0.10650	0.07483			
	M3	23	-501.866478	ω:	0.45911	0.45911	0.45911			[]
*nad*3	M0	19	-501.866375	ω0:	0.45911			M0 vs. M3	0.999999995	Not Allowed
				p:	1.00000	0.00000	0.00000			
	M2a	22	-501.866387	ω:	0.45911	1.00000	1.00000	M1a vs. M2a	0.999881007	[]

				p:	0.99999	0.00001				
	M1a	20	-501.866268	ω:	0.45910	1.00000				Not Allowed
				p0=0.99999	p=81.98217	q=96.51989				
	M8	22	-501.866338	(p1= 0.00001)	ω= 1.00000					84 P 0.509
	M7	20	-501.866396	p=	83.88435	q=	98.76095	M7 vs.M8	0.999942002	Not Allowed
				p0=0.99999	p=84.09960	q=99.00000				
	M8a	21	-501.866287	(p1= 0.00001)	ω= 1.00000			M8a vs.M8	0.991941898	Not Allowed
*nad*4				p:	0.00009	0.96497	0.03494			
	M3	23	-2065.939109	ω:	0.00000	0.00000	19.54983			[]
	M0	19	-2069.215697	ω0:	0.50705			M0 vs. M3	0.161470550	Not Allowed
				p:	0.96506	0.00000	0.03494			
	M2a	22	-2065.938447	ω:	0.00000	1.00000	19.54714			[]
				p:	0.62944	0.37056				
	M1a	20	-2068.356754	ω:	0.00000	1.00000		M1a vs. M2a	0.089072289	Not Allowed
				p0=0.96506	p=0.00500	q=5.37976				371 A 0.573,
	M8	22	-2065.938753	(p1= 0.03494)	ω= 19.54698					378 I 0.541, 439 L 0.550, 447 L 0.693, 476 P 0.575, 492 W 0.869, 493 N 0.553
	M7	20	-2068.372777	p=	0.01252	q=	0.02785	M7 vs.M8	0.087683284	Not Allowed
				p0=0.62940	p=0.00500	q=2.42461				
	M8a	21	-2068.354545	(p1= 0.37060)	ω= 1.00000			M8a vs.M8	0.027942947	Not Allowed

				p:	0.00000	0.00000	1.00000			
	M3	23	-358.123078	ω:	0.00000	0.00000	0.00000			[]
	M0	19	-358.123554	ω0:	0.00010			M0 vs. M3	0.999999887	Not Allowed
				p:	1.00000	0.00000	0.00000			
	M2a	22	-358.122936	ω:	0.00000	1.00000	1.00000			[]
*nad*4L				p:	0.99999	0.00001				
	M1a	20	-358.122995	ω:	0.00000	1.00000		M1a vs. M2a	0.999941002	Not Allowed
				p0=0.99999	p=0.00500	q=2.08857				
	M8	22	-358.123030	(p1= 0.00001)	ω= 1.00000					
	M7	20	-358.122968	p=	0.00500	q=	1.47155	M7 vs.M8	0.999938002	Not Allowed
				p0=0.99999	p=0.00500	q=2.73659				
	M8a	21	-358.123017	(p1= 0.00001)	ω= 1.00000			M8a vs.M8	0.995931589	Not Allowed
				p:	0.00000	0.99999	0.00001			
	M3	23	-1656.066160	ω:	0.00000	0.23472	0.30568			[]
	M0	19	-1656.065815	ω0:	0.23472			M0 vs. M3	0.999999941	Not Allowed
				p:	1.00000	0.00000	0.00000			
	M2a	22	-1656.066174	ω:	0.23472	1.00000	1.00000			[]
*nad*5				p:	0.99999	0.00001				
	M1a	20	-1656.065396	ω:	0.23473	1.00000		M1a vs. M2a	0.999222303	Not Allowed
				p0=0.99999	p=30.45684	q=99.00000				
	M8	22	-1656.065857	(p1= 0.00001)	ω= 2.14275					
	M7	20	-1656.065505	p=	30.45743	q=	99.00000	M7 vs.M8	0.999648062	Not Allowed
				p0=0.99999	p=30.45585	q=99.00000				
	M8a	21	-1656.065748	(p1= 0.00001)	ω= 1.00000			M8a vs.M8	0.988219804	Not Allowed

				p:	0.00000	0.97981	0.02019			
	M3	23	-910.060065	ω:	0.00000	0.21276	188.93337			[]
	M0	19	-933.994731	ω0:	0.54700			M0 vs. M3	0.000000001	Not Allowed
				p:	0.97981	0.00000	0.02019			
	M2a	22	-910.060065	ω:	0.21276	1.00000	188.93499			[]
				p:	0.82619	0.17381				
	M1a	20	-927.568802	ω:	0.00000	1.00000		M1a vs. M2a	0.000000025	Not Allowed
*nad*6				p0=0.96831	p=0.00500	q=1.98685				57 H 0.637,
	M8	22	-911.672610	(p1= 0.03169)	ω= 61.21073					200 D 0.619,<br>201 L 0.969*,<br>202 L 0.993**,<br>203 K 0.974*,<br>204 E 0.999**
	M7	20	-928.536285	p=	0.00500	q=	0.00721	M7 vs.M8	0.000000047	Not Allowed
				p0=0.82619	p=0.00500	q=1.11860				
	M8a	21	-927.568781	(p1= 0.17381)	ω= 1.00000			M8a vs.M8	0.000000017	Not Allowed
				p:	0.00007	0.97140	0.02853			
	M3	23	-1691.707133	ω:	0.00000	0.00000	13.71106			[]
	M0	19	-1697.596794	ω0:	0.34340			M0 vs. M3	0.019069995	Not Allowed
				p:	0.97147	0.00000	0.02853			
*nad*7	M2a	22	-1691.708766	ω:	0.00000	1.00000	13.71142			[]
				p:	0.79701	0.20299				
	M1a	20	-1694.996575	ω:	0.00000	1.00000		M1a vs. M2a	0.037335562	Not Allowed
	M8	22	-1691.707131	p0=0.97147	p=0.00538	q=99.00000		M7 vs.M8	0.023987558	48 Q 0.598,

				(p1= 0.02853)	ω= 13.71134					49 C 0.931,<br>71 D 0.928,<br>75 T 0.638,<br>140 N 0.591,<br>325 P 0.602
	M7	20	-1695.437351	p=	0.01293	q=	0.03102			Not Allowed
				p0=0.79702	p=0.00500	q=1.50750				
	M8a	21	-1694.996567	(p1= 0.20298)	ω= 1.00000			M8a vs.M8	0.010319627	Not Allowed
				p:	0.97327	0.00000	0.02673			
	M3	23	-827.049425	ω:	0.17842	5.11677	41.65860			[]
	M0	19	-832.438187	ω0:	0.77072			M0 vs. M3	0.029181466	Not Allowed
				p:	0.97326	0.00000	0.02674			
	M2a	22	-827.049533	ω:	0.17840	1.00000	41.65681			[]
				p:	0.66265	0.33735				
	M1a	20	-831.266715	ω:	0.00000	1.00000		M1a vs. M2a	0.014740124	Not Allowed
*nad*9				p0=0.96120	p=0.00500	q=3.50014				
	M8	22	-827.077402	(p1= 0.03880)	ω= 34.31480					2 L 0.973*,<br>3 H 0.591,<br>46 Q 0.576,<br>56 S 0.608,<br>123 S 0.603
	M7	20	-831.266717	p=	0.01552	q=	0.03337	M7 vs.M8	0.015156664	Not Allowed
				p0=0.62821	p=0.02186	q=1.61391				
	M8a	21	-831.471428	(p1= 0.37179)	ω= 1.00000			M8a vs.M8	0.003032099	Not Allowed
*rpl*5	M3	23	-913.724963	p:	0.84115	0.08915	0.06969	M0 vs. M3	0.105865357	[]

				ω:	0.07140	0.07140	2.98547			
	M0	19	-917.542895	ω0:	0.25635					Not Allowed
				p:	0.93031	0.00000	0.06969			
	M2a	22	-913.724963	ω:	0.07140	1.00000	2.98545			[]
				p:	0.78931	0.21069				
	M1a	20	-914.178164	ω:	0.00000	1.00000		M1a vs. M2a	0.635590367	Not Allowed
				p0=0.93051	p=3.43378	q=42.87778				73 S 0.867,
										82 S 0.859,
	M8	22	-913.725103	(p1= 0.06949)	ω= 2.98622					162 F 0.817
	M7	20	-914.806679	p=	0.03373	q=	0.13456	M7 vs.M8	0.339060745	Not Allowed
				p0=0.78931	p=0.00500	q=1.41189				
	M8a	21	-914.178158	(p1= 0.21069)	ω= 1.00000			M8a vs.M8	0.341148661	Not Allowed
				p:	0.00000	0.00001	0.99999			
	M3	23	-676.756795	ω:	0.00000	0.00000	1.02201			[]
	M0	19	-676.756458	ω0:	1.02196			M0 vs. M3	0.999999943	Not Allowed
				p:	0.00000	1.00000	0.00000			
	M2a	22	-676.757216	ω:	0.00000	1.00000	86.67832			[]
*rpl*10				p:	0.00001	0.99999				
	M1a	20	-676.756856	ω:	1.00000	1.00000		M1a vs. M2a	0.999640065	Not Allowed
				p0=0.00001	p=19.32291	q=99.00000				80 S 0.697,
	M8	22	-676.757109	(p1= 0.99999)	ω= 1.02199					111 R 0.698
	M7	20	-676.756888	p=	99.00000	q=	0.00500	M7 vs.M8	0.999779024	Not Allowed
				p0=0.00001	p=0.00500	q=1.96351				
	M8a	21	-676.756663	(p1= 0.99999)	ω= 1.00000			M8a vs.M8	0.976173627	Not Allowed

				p:	0.41161	0.00554	0.58285			
	M3	23	-1822.461336	ω:	0.05161	20.71909	20.71966			[]
	M0	19	-1866.108279	ω0:	0.39673			M0 vs. M3	0.000000000	Not Allowed
				p:	0.41161	0.00000	0.58839			
	M2a	22	-1822.461336	ω:	0.05161	1.00000	20.71980			[]
				p:	0.34916	0.65084				
	M1a	20	-1832.488308	ω:	0.00000	1.00000		M1a vs. M2a	0.000044192	Not Allowed
				p0=0.38370	p=0.00500	q=1.78454				3 A 0.765,<br>4 L 0.843,<br>5 R 0.864,<br>20 E 0.575,<br>39 I 0.914,
*rps*4										49 L 0.864,<br>65 H 0.986*,<br>66 G 0.987*,<br>67 D 0.976*,<br>68 L 0.638,<br>69 P 0.993**,<br>70 I 0.779,<br>71 T 0.883,<br>72 E 0.957*,<br>73 M 0.993**,<br>74 H 0.955*,<br>75 R 0.630,
	M8	22	-1822.843427	(p1= 0.61630)	ω= 21.42471			M7 vs.M8	0.000059319	76 G 0.932,

			77 T 0.993**,<br>79 R 0.902,<br>80 T 0.958*,<br>82 Y 0.994**,<br>83 I 0.842,<br>86 P 0.909,<br>87 L 0.790,<br>88 N 0.849,<br>89 P 0.993**,<br>90 E 0.616,<br>91 T 0.870,<br>92 R 0.984*,<br>93 S 0.997**,<br>95 V 0.991**,<br>96 I 0.749,<br>98 V 0.936,<br>99 R 0.993**,<br>100 L 0.928,<br>101 H 0.976*,<br>102 F 0.991**,<br>103 S 0.972*,<br>104 E 0.996**,<br>105 T 0.634,<br>106 I 0.863,<br>108 Q 0.958*,

			111 Q 0.995**,<br>113 I 0.990*,<br>114 S 0.984*,<br>115 H 0.989*,<br>116 R 0.921,<br>117 R 0.997**,<br>118 L 0.805,<br>119 C 0.569,<br>120 V 0.946,<br>121 N 0.995**,<br>122 N 0.950,<br>123 V 0.701,<br>124 I 0.857,<br>125 V 0.860,<br>126 S 0.929,<br>127 I 0.993**,<br>128 T 0.955*,<br>130 F 0.866,<br>131 Q 0.905,<br>132 V 0.965*,<br>133 S 0.991**,<br>134 Q 0.875,<br>136 D 1.000**,<br>137 F 0.696,<br>138 I 0.892,

									140 L 0.998**,<br>142 E 0.951*,<br>143 N 0.994**,<br>144 D 0.975*,<br>145 A 0.931,<br>147 I 0.986*,<br>148 Y 0.992**,<br>149 S 0.926,<br>150 E 0.988*,<br>152 R 0.948,<br>153 R 0.993**,<br>154 S 0.999**,<br>155 F 0.987*,<br>156 Y 0.992**,<br>157 I 0.778,<br>159 I 0.795,<br>160 S 0.993**,<br>162 S 0.975*,<br>163 K 0.903,<br>165 I 0.983*,<br>166 G 0.835,<br>167 K 0.897,<br>168 S 0.998**,<br>169 L 0.997**,<br>170 D 0.995**,

			171 S 0.984*,<br>173 V 0.987*,<br>174 R 0.917,<br>175 M 0.992**,<br>177 R 0.984*,<br>178 R 0.829,<br>179 T 0.995**,<br>180 K 0.980*,<br>181 T 0.878,<br>183 L 0.994**,<br>185 K 0.939,<br>186 T 0.720,<br>187 K 0.984*,<br>190 C 0.997**,<br>191 R 0.997**,<br>192 L 0.983*,<br>194 L 0.990*,<br>195 K 0.804,<br>196 L 0.677,<br>197 Q 0.926,<br>199 L 0.675,<br>201 S 0.995**,<br>202 S 0.994**,<br>203 M 0.914,<br>204 Q 0.817,

										205 D 0.889,<br>206 E 0.975*,<br>207 D 0.807,<br>209 R 0.592,<br>210 T 0.917,<br>211 K 0.595,<br>212 K 0.942,<br>215 S 0.998**,<br>219 C 0.758,<br>220 L 0.558,<br>221 G 0.941,<br>222 S 0.928,<br>223 S 0.867,<br>225 I 0.990*,<br>226 V 0.991**,<br>227 Y 0.987*,<br>228 N 0.968*,<br>231 R 0.978*
	M7	20	-1832.576007	p=	0.02012	q=	0.01137			Not Allowed
	M8a	21	-1832.488291	p0=0.34916	p=0.00500	q=2.91075		M8a vs.M8	0.000011231	Not Allowed
				(p1= 0.65084)	ω= 1.00000					
*rps*12	M3	23	-502.658002	p:	0.88551	0.02998	0.08451			[]
				ω:	0.00000	0.00000	0.00000			
	M0	19	-502.659318	ω0:	0.00010			M0 vs. M3	0.999999135	Not Allowed
	M2a	22	-502.657949	p:	1.00000	0.00000	0.00000	M1a vs. M2a	0.99998	[]

				ω:	0.00000	1.00000	1.00000			
				p:	0.99999	0.00001				
	M1a	20	-502.657969	ω:	0.00000	1.00000				Not Allowed
				p0=0.99999	p=0.00500	q=2.39690				
	M8	22	-502.658171	(p1= 0.00001)	ω= 1.00000					
	M7	20	-502.657839	p=	0.00500	q=	1.90486	M7 vs.M8	0.999668055	Not Allowed
				p0=0.99999	p=0.00500	q=1.98502				
	M8a	21	-502.658044	(p1= 0.00001)	ω= 1.00000			M8a vs.M8	0.987284351	Not Allowed
				p:	0.85163	0.13350	0.01487			
	M3	23	-474.464005	ω:	0.00000	0.00000	10.21891			[]
	M0	19	-474.532516	ω0:	0.09061			M0 vs. M3	0.997757608	Not Allowed
				p:	0.98513	0.00000	0.01487			
	M2a	22	-474.464091	ω:	0.00000	1.00000	10.22046			[]
*rps*13				p:	0.90510	0.09490				
	M1a	20	-474.519921	ω:	0.00000	1.00000		M1a vs. M2a	0.945699891	Not Allowed
				p0=0.98513	p=0.00500	q=4.35450				
	M8	22	-474.464004	(p1= 0.01487)	ω= 10.21870					
	M7	20	-474.529846	p=	0.05338	q=	0.33652	M7 vs.M8	0.936278785	Not Allowed
				p0=0.90510	p=0.00500	q=1.88275				
	M8a	21	-474.519949	(p1= 0.09490)	ω= 1.00000			M8a vs.M8	0.738002573	Not Allowed

Table S12. Regions for designing PCR primers to distinguish the *Artemisia* species.

ID	Species Name	Accession Number	Positions on the Plastomes (start-end)		The sequence of the regions to design the forward primers	The sequence of the regions to design the reverse primers
			Forward Regions	Reverse Regions		
1	*A. absinthium var. calcigena*	MK188885.1	79729-79798	110048-110136	CAGCAACAATAACGTCACCAATACGAGCATATCGACGATTGCTAGCTCCTATGATTCGAATACACATCAA	ACGAAGACTTTTTGTTGCCGTCGGAAAAATAAGAAGTCCCAACCCTATTAACATAGGAACTGGAAGTGGAAGAAAAGGTATTATCCACG
	A. tangutica	MT701043.1	79632-79701	110017-110105		
	A. lactiflora	MW411453.1	79714-79783	110100-110188		
	A. frigida	NC_020607.1	79503-79572	109903-109991		
	A. montana	NC_025910.1	79600-79669	110004-110092		
	A. argyi	NC_030785.1	79672-79741	110061-110149		
	A. gmelinii	NC_031399.1	79808-79877	110194-110282		
	A. capillaris	NC_031400.1	79565-79634	109972-110060		
	A. annua	NC_034683.1	79534-79603	109907-109995		
	A. selengensis	NC_039647.1	79679-79748	110056-110144		
	A. fukudo	NC_044156.1	79519-79588	109890-109978		
	A. maritima	NC_045093.1	79542-79611	109939-110027		
	A. scoparia	NC_045286.1	79582-79651	109976-110064		
	A. ordosica	NC_046571.1	79736-79805	110131-110219		
	A. freyniana	NC_049570.1	79726-79795	110122-110210		
	A. hallaisanensis	NC_049571.1	79576-79645	109938-110026		
	A. stolonifera	NC_049572.1	79615-79684	110010-110098		
	A. giraldii	OK128342.1	79595-79664	109960-110048		

Table S13 Size difference between mitogenomes and plastomes available in GenBank by August 1st, 2022. For multichromosomal mitochondrial genomes, the number in front of the mitogenome accession number represents the chromosome number. The mitogenome size of the species with multichromosomal mitochondrial genomes was the sum of their chromosome lengths. The species were ordered based on the size differences between their mitogenomes and plastomes.

Species	Mitogenome size (bp)	Mitogenome accession number	Plastome size (bp)	Plastome accession number	Difference length (bp)
Haematococcus lacustris	124,604	NC_044670.1	1352310	NC_044670.1	-1227706
Yamagishiella unicocca	30876	NC_033969.1	300175	NC_039754.1	-269299
Leontynka pallida	104812	NC_063674.1	362307	NC_063675.1	-257495
Pleodorina starrii	20375	NC_021108.1	269857	NC_021109.1	-249482
Dunaliella salina	28331	NC_012930.1	269044	NC_016732.1	-240713
Gonium pectorale	15993	NC_020437.1	222582	NC_020438.1	-206589
Pseudomuriella schumacherensis	43134	NC_024763.1	220357	NC_029669.1	-177223
Jenufa perforata	27198	NC_046779.1	198040	NC_028581.1	-170842
Jenufa minuta	41488	NC_046780.1	206680	NC_028582.1	-165192
Pectinodesmus pectinatus	32195	NC_036659.1	196809	NC_036668.1	-164614
Chlorotetraedron incus	38406	NC_024757.1	193197	NC_029673.1	-154791
Chlamydomonas leiostraca	14029	NC_026573.1	167394	NC_032109.1	-153365
Entransia fimbriata	61645	NC_022861.1	206025	NC_030313.1	-144380
Chromochloris zofingiensis	44840	NC_024758.1	188935	NC_029672.1	-144095
Neochloris aquatica	38021	NC_024761.1	166767	NC_029670.1	-128746
Tetradesmus obliquus	41704	CM007918.1	167272	CM007919.1	-125568
Bracteacoccus aerius	47158	NC_024755.1	165732	NC_029675.1	-118574
Chara vulgaris	67737	NC_005255.1	184933	NC_008097.1	-117196

Coccomyxa sp. Obi	64287	AP025007.1	177965	AP025008.1	-113678
Coccomyxa subellipsoidea	65497	NC_015316.1	175731	NC_015084.1	-110234
Pseudendoclonium akinetum	95880	NC_005926.1	195867	NC_008114.1	-99987
Oltmannsiellopsis viridis	56761	NC_008256.1	151933	NC_008099.1	-95172
Botryococcus braunii	84583	NC_027722.1	172826	NC_025545.1	-88243
Mychonastes homosphaera	25149	NC_024760.1	102718	NC_029671.1	-77569
Trebouxiophyceae sp.	74423	NC_018568.1	149707	NC_018569.1	-75284
Klebsormidium nitens	106468	DF238763.1	181482	DF238762.1	-75014
Chaetosphaeridium globosum	56574	NC_004118.1	131183	NC_004115.1	-74609
Chlorella vulgaris	91560	CM041648.1	165504	CM041649.1	-73944
Roya anglica	69371	NC_046950.1	138275	NC_024168.1	-68904
Roya obtusa	69465	NC_022863.1	138272	NC_030315.1	-68807
Scherffelia dubia	78958	NC_045363.1	137161	NC_029807.1	-58203
Chlorella sorokiniana	52528	NC_024626.1	109940	CM017271.1	-57412
Pycnococcus provasolii	24321	NC_013935.1	80211	NC_012097.1	-55890
Micractinium conductrix	95661	CM009643.1	149364	CM009644.1	-53703
Monomastix sp.	60883	NC_022797.1	114528	NC_012101.1	-53645
Ulva gigantea	66743	NC_053630.1	117606	NC_053613.1	-50863
Closterium baillyanum	152089	NC_022860.1	201341	NC_030314.1	-49252
Chlorella variabilis	78500	NC_025413.1	124579	NC_015359.1	-46079
Ulva australis	64466	NC_053628.1	109820	NC_053611.1	-45354
Monoraphidium neglectum	93840	NW_014013625.1	135362	NW_014013626.1	-41522
Picochlorum costavermella	34178	CM022108.1	74290	CM022109.1	-40112
Picocystis salinarum	41858	NC_042491.1	81133	NC_024828.1	-39275
Ulva fenestrata U64	59026	NC_053629.1	94654	NC_053612.1	-35628

Ulva fasciata	61614	NC_028081.1	96005	NC_029040.1	-34391
Ulva compressa	62477	NC_041082.1	96808	NC_050739.1	-34331
Picochlorum sp. 'soloecismus'	38692	CM008992.1	72761	CM008993.1	-34069
Chloroparvula japonica	40432	NC_042601.1	71262	NC_042487.1	-30830
Syntrichia filaris	106343	KP984758.1	136227	NC_050352.1	-29884
Ulva pseudorotundata	88416	NC_053633.1	118206	NC_053616.1	-29790
Ulva rigida	88416	NC_053633.1	118206	NC_053616.1	-29790
Ulva prolifera	63845	KT428794.1	93066	NC_036137.1	-29221
Bathycoccus prasinos	43614	NC_023273.1	72700	NC_024811.1	-29086
Chloropicon mariensis	35545	NC_042600.1	64323	NC_042485.1	-28778
Chloropicon maureeniae	36041	NC_042602.1	64094	NC_042488.1	-28053
Ostreococcus tauri	44237	NC_008290.1	71666	NC_008289.1	-27429
Auxenochlorella protothecoides	57274	NC_026009.1	84576	NC_023775.1	-27302
Chloropicon sieburthii	37591	NC_042598.1	64565	NC_042483.1	-26974
Chloropicon laureae	37769	NC_042492.1	64740	NC_042484.1	-26971
Chloropicon roscoffensis	38736	NC_042599.1	64534	NC_042486.1	-25798
Pohlia nutans	99864	NC_046778.1	125199	NC_045869.1	-25335
Micromonas commoda	47425	NC_012643.1	72585	NC_012575.1	-25160
Ostreococcus mediterraneus	49312	CM022724.1	74215	CM022725.1	-24903
Ulva laetevirens	79723	NC_053631.1	103444	NC_053614.1	-23721
Prasinoderma coloniale	54546	NC_023355.1	77750	NC_024817.1	-23204
Buxbaumia aphylla	100725	KC784954.1	123907	NC_046056.1	-23182
Hypnum imponens	103830	KC784951.1	125195	NC_058545.1	-21365
Anomodon attenuatus	104252	JX402749.1	125282	NC_058540.1	-21030
Climacium dendroides	104860	NC_053886.1	124957	NC_051864.1	-20097

Chloroparvula pacifica	49744	NC_042603.1	69644	NC_042489.1	-19900
Sanionia uncinata	104497	NC_027974.1	124374	NC_025668.1	-19877
Tetraphis pellucida	107730	NC_024290.1	127489	NC_024291.1	-19759
Bartramia pomiformis	106198	NC_024519.1	125886	NC_050047.1	-19688
Myurella julacea	104979	NC_054351.1	124457	NC_053275.1	-19478
Orthotrichum stellatum	104131	KC784958.1	123253	NC_058546.1	-19122
Ulota hutchinsiae	104608	KC784952.1	123615	NC_058548.1	-19007
Ptychomnion cygnisetum	104480	KC784949.1	123452	NC_058547.1	-18972
Stoneobryum bunyaense	104352	NC_031392.1	123040	NC_042479.1	-18688
Nyholmiella obtusifolia	104603	NC_031767.1	122895	NC_026979.1	-18292
Ulva flexuosa	71545	NC_035809.1	89414	NC_035823.1	-17869
Physcomitrium patens	105340	NC_007945.1	122905	NC_037465.1	-17565
Ulva linza	70858	NC_029701.1	86726	NC_030312.1	-15868
Funaria hygrometrica	109586	KC784959.1	122213	NC_058544.1	-12627
Polytrichum commune	114831	NC_039775.1	126323	NC_060348.1	-11492
Cymbomonas tetramitiformis	73520	NC_036614.1	84524	NC_030169.1	-11004
Atrichum angustatum	115146	KC784956.1	125602	NC_058541.1	-10456
Sphagnum palustre	141276	NC_024521.1	140040	NC_030198.1	1236
Prototheca wickerhamii	52179	CM009948.1	48161	CM009949.1	4018
Prototheca zopfii	38164	NC_037449.1	28698	NC_037450.1	9466
Douinia plicata	144205	NC_054214.1	118797	MT898431.1	25408
Scapania ampliata	143664	NC_052751.1	118026	MT644123.1	25638
Diplophyllum taxifolium	144129	NC_054215.1	118309	MT948954.1	25820
Nothoceros aenigmaticus	184908	NC_012651.1	153208	NC_020259.1	31700
Bidens pilosa	183061	NC_062673.1	150542	MN729611.1	32519

Nowellia curvifolia	148199	NC_063925.1	114423	NC_063926.1	33776
Gymnomitrion concinnatum	162572	NC_040132.1	120994	NC_040133.1	41578
Artemisia giraldii	194298	NC_064134.1	151072	OK128342.1	43226
Bidens parviflora	195825	NC_062670.1	151314	NC_060633.1	44511
Aneura pinguis	165603	NC_026901.1	120698	NC_035617.1	44905
Bidens biternata	198476	NC_062672.1	151487	NC_060634.1	46989
Bidens bipinnata	198476	NC_060635.1	151486	NC_062669.1	46990
Zostera marina	191481	NC_035345.1	143877	NC_036014.1	47604
Chlorokybus atmophyticus	201763	NC_009630.1	152254	NC_008822.1	49509
Dumortiera hirsuta	178019	NC_042873.1	122050	NC_039590.1	55969
Leiosporoceros dussii	212153	NC_039751.1	155956	NC_039750.1	56197
Chrysanthemum indicum	208097	MH716014.1	150972	NC_020320.1	57125
Spirodela polyrhiza	228493	NC_017840.1	168788	NC_015891.1	59705
Chrysanthemum boreale	211002	NC_039757.1	151012	NC_037388.1	59990
Caulerpa ashmeadii	197427	NC_045849.1	135722	NC_045914.1	61705
Riccia fluitans	185621	NC_043906.1	121682	NC_042887.1	63939
Wiesnerella denudata	186911	NC_053538.1	122500	MT712073.1	64411
Marchantia paleacea	186609	NC_001660.1	121024	NC_001319.1	65585
Marchantia polymorpha subsp. *ruderalis*	186196	NC_037508.1	120304	NC_037507.1	65892
Brassica rapa	219736	NC_049892.1	153483	NC_040849.1	66253
Brassica juncea	219766	NC_016123.1	153483	NC_028272.1	66283
Bidens tripartita	216786	NC_062671.1	150489	NC_058915.1	66297
Anthoceros agrestis	228021	NC_049004.1	160790	NC_049002.1	67231
Anthoceros punctatus	228048	NC_049003.1	160692	NC_049001.1	67356
Ageratum conyzoides	219198	NC_053927.1	151325	MK905238.1	67873

Brassica carinata	232241	JF920287.1	153641	NC_059807.1	78600
Brassica nigra	232407	NC_029182.1	153633	NC_030450.1	78774
Anthoceros angustus	242410	NC_037476.1	161162	NC_004543.1	81248
Sinapis arvensis	240024	NC_031896.1	153590	NC_035303.1	86434
Caulerpa lentillifera	209034	NC_038217.1	119402	NC_039377.1	89632
Silene latifolia	253413	NC_014487.1	151736	NC_016730.1	101677
Ipomoea nil	265768	NC_031158.1	161897	NC_031159.1	103871
Raphanus sativus	258426	NC_018551.1	153368	NC_024469.1	105058
Agrostemma githago	262903	NC_057604.1	151733	KF527884.1	111170
Mirabilis jalapa	267334	NC_056991.1	154480	MK397875.1	112854
Boechera stricta	271601	MH545496.1	155033	NC_049599.1	116568
Fragaria moschata	277672	NC_062590.1	155601	NC_062593.1	122071
Helianthus grosseserratus	273543	NC_051989.1	151017	NC_023108.1	122526
Daucus carota subsp. *sativus*	281132	NC_017855.1	155911	NC_008325.1	125221
Diplostephium hartwegii	277718	NC_034354.1	151994	NC_034832.1	125724
Calystegia soldanella	279265	NC_060803.1	152317	NC_060788.1	126948
Fragaria orientalis	275143	NC_057524.1	147835	KY769126.1	127308
Fragaria tibetica	283001	NC_062832.1	155643	NC_062835.1	127358
Fragaria gracilis	283031	NC_062834.1	155630	NC_062837.1	127401
Helianthus strumosus	281056	NC_051990.1	151044	NC_023113.1	130012
Helianthus tuberosus	281287	NC_058585.1	151047	KF746361.1	130240
Capsella rubella	287405	NC_042883.1	154601	NC_027693.1	132804
Tolypanthus maclurei	256961	NC_056836.1	123581	NC_042257.1	133380
Fragaria viridis	289289	NC_062592.1	155458	MH938454.1	133831
Coleochaete scutata	242024	NC_045180.1	107236	NC_030358.1	134788

Potentilla anserina	294593	OW176988.1	155659	NC_060513.1	138934
Fragaria nubicola	299474	NC_062587.1	155608	MZ851763.1	143866
Saposhnikovia divaricata	293897	NC_058846.1	147834	MN857472.1	146063
Helianthus annuus	300945	NC_023337.1	151104	NC_007977.1	149841
Trifolium pratense	301823	NC_048499.1	146573	NC_047412.1	155250
Rosa chinensis	313448	CM009589.1	156546	CM009590.1	156902
Fragaria nilgerrensis	315211	NC_062589.1	155783	MZ851761.1	159428
Arctium lappa	312598	NC_058644.1	152708	MH671331.1	159890
Coriandrum sativum	307516	1：NC_059794.1<br>2：MW477238.1	146519	KR002656.1	160997
Panax vietnamensis	317650	1：MZ826164.1<br>2：MZ826165.1<br>3：MZ826166.1	155993	KP036470.1	161657
Allium cepa	316363	NC_030100.1	153538	NC_024813.1	162825
Chenopodium quinoa	315003	NC_041093.1	152099	NC_034949.1	162904
Trifolium aureum	294911	NC_048502.1	126970	NC_024035.1	167941
Polygonum aviculare	333387	OW204033.1	163448	NC_058892.1	169939
Fragaria iinumae	328819	NC_062591.1	155554	KC507759.1	173265
Nymphaea hybrid	335042	NC_060361.1	159968	NC_060360.1	175074
Spinacia oleracea	329613	NC_035618.1	150725	NC_002202.1	178888
Lagerstroemia indica	333948	NC_035616.1	152205	NC_030484.1	181743
Aquilaria sinensis	341829	NC_054354.1	159565	KT148967.1	182264
Ammopiptanthus nanus	339352	NC_046466.1	154140	NC_034743.1	185212
Evolvulus alsinoides	344184	NC_058741.1	157015	NC_058590.1	187169
Bougainvillea spectabilis	343746	NC_056281.1	154541	MK397858.1	189205

Ginkgo biloba	346544	NC_027976.1	156988	NC_016986.1	189556
Mirabilis himalaica	346363	NC_048974.1	154348	NC_048975.1	192015
Fragaria iturupensis	351183	NC_062833.1	155637	NC_062836.1	195546
Salvia splendens	347308	CM031494.1	150604	NC_050901.1	196704
Scyphiphora hydrophyllacea	354155	NC_057654.1	155132	NC_049078.1	199023
Ajuga reptans	352069	NC_023103.1	149963	NC_023102.1	202106
Morus notabilis	362069	NC_041177.1	158680	KP939360.1	203389
Ziziphus jujuba	365190	NC_029809.1	161466	NC_030299.1	203724
Ziziphus jujuba var. spinosa	365812	CM036902.1	161185	CM036903.1	204627
Trifolium meduseum	348724	NC_048500.1	142595	NC_024166.1	206129
Brassica oleracea	360271	JF920286.1	153364	NC_041167.1	206907
Lactuca sativa	363324	NC_042756.1	152765	NC_007578.1	210559
Arabidopsis thaliana	367808	NC_037304.1	154478	NC_000932.1	213330
Beta vulgaris subsp. *maritima*	364950	FP885845.1	149724	NC_059015.1	215226
Beta vulgaris subsp. *vulgaris*	368801	NC_002511.2	149723	NC_059012.1	219078
Apium graveolens	371275	NC_058313.1	152050	MK036045.1	219225
Trifolium grandiflorum	347723	NC_048501.1	125628	NC_024034.1	222095
Sorbus aucuparia	384977	NC_052880.1	160108	NC_052878.1	224869
Sorbus torminalis	386758	NC_052879.1	160390	NC_033975.1	226368
Xanthoceras sorbifolium	388931	CM036794.1	161231	NC_037448.1	227700
Quercus robur	390906	OW028777.1	161172	NC_046388.1	229734
Prunus avium	389709	NC_044768.1	157886	MK622380.1	231823
Pisum abyssinicum	354692	NC_059791.1	122174	NC_037830.1	232518
Ophioglossum californicum	372339	NC_030900.1	138270	NC_020147.1	234069
Beta macrocarpa	385220	FQ378026.1	149727	NC_059016.1	235493

Malus domestica	396947	NC_018554.1	160288	NC_061549.1	236659
Nicotiana attenuata	394341	NC_036467.1	155886	MF577082.1	238455
Pisum sativum	363498	CM044352.1	122169	NC_014057.1	241329
Phaseolus vulgaris	395516	NC_045135.1	150285	NC_009259.1	245231
Vigna radiata	401262	NC_015121.1	151271	NC_013843.1	249991
Glycine soja	402545	NC_039768.1	152217	NC_022868.1	250328
Glycine max	402558	NC_020455.1	152218	NC_007942.1	250340
Cycas taitungensis	414903	NC_010303.1	163403	NC_009618.1	251500
Vigna angularis	404466	NC_021092.1	151683	NC_021091.1	252783
Pisum fulvum	379922	NC_059792.1	120837	NC_036828.1	259085
Cannabis sativa	415602	NC_029855.1	153854	NC_027223.1	261748
Aegiceras corniculatum	425282	NC_056358.1	157241	MN167882.1	268041
Solanum pennellii	423596	NC_035964.1	155254	KY887589.1	268342
Millettia pinnata	425718	NC_016742.1	152968	NC_016708.2	272750
Nicotiana tabacum	430597	NC_006581.1	155943	NC_001879.2	274654
Eriobotrya japonica	434980	NC_045228.1	159137	NC_034639.1	275843
Vaccinium macrocarpon	459678	NC_023338.1	176045	NC_019616.1	283633
Aconitum kusnezoffii	440720	NC_053920.1	155862	KT820671.1	284858
Senna occidentalis	447106	NC_038221.1	159993	NC_038222.1	287113
Solanum lycopersicum	446257	NC_035963.1	155461	NC_007898.3	290796
Butomus umbellatus	450826	NC_021399.1	158107	NC_051949.1	292719
Saccharum officinarum	445555	1：OK037503.1<br>2：OK037504.1	141176	NC_035224.1	304379
Bupleurum falcatum	463792	NC_035962.1	155989	KM207676.1	307803
Carica papaya	476890	NC_012116.1	160100	NC_010323.1	316790

Triticum aestivum	452526	NC_036024.1	134545	NC_002762.1	317981
Eucalyptus grandis	478813	NC_040010.1	160137	NC_014570.1	318676
Ammopiptanthus mongolicus	475396	NC_039660.1	153935	KY034453.1	321461
Suaeda glauca	474330	NC_060419.1	149807	NC_045303.1	324523
Styphnolobium japonicum	484916	NC_039596.1	158656	NC_047059.1	326260
Prunus salicina x Prunus armeniaca	484858	NC_060491.1	157916	NC_060490.1	326942
Sorghum bicolor	468628	NC_008360.1	140754	NC_008602.1	327874
Solanum melongena	482343	NC_050334.1	154289	KU682719.1	328054
Epirixanthes elongata	365168	NC_046014.1	36275	NC_046014.1	328893
Asparagus officinalis	492062	NC_053642.1	156699	NC_053642.1	335363
Glycyrrhiza uralensis	463869	NC_053919.1	127887	NC_053919.1	335982
Castilleja paramensis	495499	NC_031806.1	152926	NC_031806.1	342573
Buddleja alternifolia	499191	CM033409.1	154280	CM033409.1	344911
Fagus sylvatica	504715	NC_050960.1	158462	NC_050960.1	346253
Salvia miltiorrhiza	499236	NC_023209.1	151328	NC_023209.1	347908
Capsicum annuum	511530	NC_024624.1	156781	NC_024624.1	354749
Oryza sativa Japonica Group	490520	NC_011033.1	134525	NC_011033.1	355995
Oryza sativa Indica Group	491515	NC_007886.1	134496	NC_007886.1	357019
Boea hygrometrica	510519	NC_016741.1	153493	NC_016468.1	357026
Ilex pubescens	517520	MK714017.1	157872	MK714017.1	359648
Nelumbo nucifera	524797	NC_030753.1	163330	NC_030753.1	361467
Nepenthes ventricosa x Nepenthes alata	520764	NC_039531.1	156637	NC_039531.1	364127
Citrus maxima	538434	NC_057143.1	160133	NC_057143.1	378301
Oryza minuta	515022	NC_029816.1	135094	NC_030298.1	379928
Zea mays	525405	CM025451.1	140384	NC_001666.2	385021

Eleusine indica	520691	NC_040989.1	135151	NC_030486.1	385540
Rhazya stricta	548608	NC_024293.1	154841	NC_024292.1	393767
Liriodendron tulipifera	553721	NC_021152.1	159886	NC_008326.1	393835
Capsicum chinense	552407	1：MZ736638.1	156807	KU041709.1	395600
		2：MZ736639.1			
Zea luxurians	539368	NC_008333.1	140710	NC_030301.1	398658
Malania oleifera	527575	NC_053625.1	125050	MG799332.1	402525
Senna tora	566589	NC_038053.1	162426	NC_030193.1	404163
Hibiscus cannabinus	569915	NC_035549.1	162903	NC_045873.1	407012
Osmanthus fragrans	563202	NC_060346.1	155896	NC_042377.1	407306
Solanum aethiopicum	566695	NC_050335.1	155608	MH283717.1	411087
Chrysopogon zizanioides	551622	NC_056367.1	139971	KY596158.1	411651
Betula pendula	581505	LT855379.1	161148	LT855378.1	420357
Oryza rufipogon	559045	NC_013816.1	134544	NC_017835.1	424501
Zea perennis	570354	NC_008331.1	140647	NC_030300.1	429707
Gleditsia sinensis	594121	NC_058235.1	163175	MK817503.1	430946
Avicennia marina	578994	CM032784.1	147909	NC_047414.1	431085
Bombax ceiba	594390	NC_038052.1	158997	NC_037494.1	435393
Viscum album	565432	NC_029039.1	128921	NC_028012.1	436511
Sapindus mukorossi	602121	NC_050850.1	160481	NC_025554.1	441640
Salix purpurea	598970	NC_029693.1	155590	KP019639.1	443380
Libidibia coriaria	601574	NC_045039.1	158045	KJ468095.1	443529
Panax quinquefolius	600905	1：MZ826160.1	156088	KM088018.1	444817
		2：MZ826161.1			
		3：MZ826162.1			

		4：MZ826163.1			
Tamarindus indica	607282	NC_045038.1	159551	KJ468103.1	447731
Salix brachista	608983	NC_058733.1	155600	NC_058984.1	453383
Nymphaea colorata	617195	NC_037468.1	159842	NC_057562.1	457353
Haematoxylum brasiletto	631094	NC_045040.1	157616	NC_047060.1	473478
Citrus sinensis	640906	NC_037463.1	160129	NC_008334.1	480777
Gossypium thurberi	644395	NC_035074.1	160264	NC_015204.1	484131
Gossypium davidsonii	644311	NC_035075.1	160072	NC_033395.1	484239
Gossypium trilobum	644460	NC_035076.1	160109	NC_033397.1	484351
Salix suchowensis	644437	NC_029317.1	155214	NC_026462.1	489223
Psilotum nudum	628553	1：NC_030952.1	138829	NC_003386.1	489724
		2：KX171639.1			
Hesperelaea palmeri	658522	NC_031323.1	155820	NC_025787.1	502702
Gossypium harknessii	666081	NC_027407.1	160129	NC_033333.1	505952
Gossypium hirsutum	668584	NC_027406.1	160301	NC_007944.1	508283
Spondias mombin	674158	NC_045035.1	162302	KY828469.1	511856
Gossypium raimondii	676078	NC_029998.1	160161	NC_016668.1	515917
Gossypium barbadense	677434	NC_028254.1	160317	NC_008641.1	517117
Manihot esculenta	682840	NC_045136.1	161453	NC_010433.1	521387
Macadamia integrifolia	682814	NC_058888.1	159714	NC_025288.1	523100
Asclepias syriaca	682498	NC_022796.1	158719	NC_022432.1	523779
Acacia ligulata	698138	NC_040998.1	174233	NC_026134.2	523905
Cocos nucifera	678653	NC_031696.1	154731	NC_022417.1	523922
Gossypium arboreum	687482	NC_035073.1	160230	NC_016712.1	527252
Physochlaina orientalis	684857	NC_044153.1	156321	NC_044154.1	528536

Passiflora edulis	680480	NC_050950.1	151406	NC_034285.1	529074
Solanum tuberosum	688698	1：MW594251.1<br>2：MW594252.1<br>3：MW594253.1<br>4：MW594254.1	155296	NC_008096.2	533402
Camellia sinensis	707441	NC_043914.1	157103	NC_020019.1	550338
Salix dunnii	711422	NC_058734.1	155647	NC_058985.1	555775
Salix wilsonii	711456	NC_064688.1	155026	MK748469.1	556430
Phoenix dactylifera	715001	NC_016740.1	158462	NC_013991.2	556539
Leucaena trichandra	722009	NC_039738.1	164692	NC_028733.1	557317
Tripsacum dactyloides	704100	NC_008362.1	141050	NC_037087.1	563050
Mangifera longipes	728635	NC_060990.1	157853	NC_057291.1	570782
Geranium maderense	737091	NC_027000.1	155694	NC_029999.1	581397
Olea europaea subsp. *europaea*	755572	LR743801.1	155658	LR743800.1	599914
Vitis vinifera	773279	NC_012119.1	160928	NC_007957.1	612351
Spondias tuberosa	779106	NC_045036.1	162039	NC_030527.1	617067
Populus davidiana	779361	NC_035157.1	155853	NC_032717.1	623508
Populus tremula x Populus alba	783513	NC_028329.1	156641	NC_028504.1	626872
Populus tremula	783442	NC_028096.1	156067	NC_027425.1	627375
Acer yangbiense	803281	NC_059858.1	155706	MN652924.1	647575
Rhododendron simsii	802707	NC_053763.1	152214	NC_053764.1	650493
Populus alba	838420	NC_041085.1	156505	NC_008235.1	681915
Tetracentron sinense	856059	NC_059859.1	164467	KC608752.1	691592
Cuscuta japonica	813731	NC_060804.1	120975	NC_060789.1	692756
Populus tomentosa	853269	1：MZ707547.1	156446	MK251149.1	696823

		2：MZ707545.1			
		3：MZ707546.1			
		4：MZ707544.1			
Populus adenopoda	869108	1：MZ707540.1	158591	KX425622.1	710517
		2：MZ707541.1			
		3：MZ707543.1			
		4：MZ707542.1			
Mangifera indica	871458	CM021857.1	157780	NC_035239.1	713678
Magnolia biondii	967100	NC_049134.1	160002	NC_034687.1	807098
Cyperus esculentus	1002700	NC_058697.1	186255	NC_058698.1	816445
Welwitschia mirabilis	978846	NC_029130.1	119726	NC_010654.1	859120
Cynanchum auriculatum	1083752	1：MH931260.1	160840	NC_029460.1	922912
		2：NC_041494.1			
		3：MH410148.1			
Schisandra sphenanthera	1101770	NC_042758.1	146843	NC_037145.1	954927
Anemone maxim	1122550	NC_053368.1	160876	NC_045909.1	961674
Pinus taeda	1191050	NC_039746.1	121530	NC_021440.1	1069520
Platycodon grandiflorus	1249590	NC_035958.1	171818	KX352464.1	1077772
Panax notoginseng	1538907	1：MZ826156.1	156387	NC_026447.1	1382520
		2：MZ826157.1			
		3：MZ826158.1			
		4：MZ826159.1			
Cucumis sativus	1684597	1：NC_016005.1	155293	NC_007144.1	1529304
		2：NC_016004.1			
		3：NC_016006.1			

Table S14 The result of sequence alignment using the BLASTN suite in NCBI (https://blast.ncbi.nlm.nih.gov/Blast.cgi). The query sequence is the *A. giraldii* mitogenome (NC_064134.1).

Scientific Name	Max Score	Total Score	Query Cover	E value	Per. ident	Acc. Len	Accession
Artemisia giraldii	3.59E+05	3.74E+05	100%	0	100	194298	NC_064134.1
Artemisia giraldii	3.59E+05	3.74E+05	100%	0	100	194298	OK268247.2
Chrysanthemum zawadskii	54106	4.46E+05	93%	0	99.62	277934	ON053202.1
Chrysanthemum makinoi	53681	3.33E+05	92%	0	99.37	223189	OU343227.1
Chrysanthemum boreale	52532	3.29E+05	90%	0	99.51	211002	NC_039757.1
Chrysanthemum boreale	52532	3.29E+05	90%	0	99.51	211002	MH004292.1
Chrysanthemum indicum	32725	3.25E+05	90%	0	99.53	208097	MH716014.1
Diplostephium hartwegii	22341	2.78E+05	79%	0	98.43	277718	KX063855.1
Paraprenanthes diversifolia	18044	3.08E+05	78%	0	98.94	360751	MN661146.1
Lactuca virosa	18044	3.17E+05	77%	0	98.94	373019	MZ159960.1
Lactuca virosa	18044	3.17E+05	77%	0	98.94	373019	MZ159959.1
Lactuca serriola	18044	3.21E+05	77%	0	98.94	367647	MZ159958.1
Lactuca serriola	18044	3.21E+05	77%	0	98.94	367647	MZ159957.1
Lactuca sativa	18039	3.16E+05	77%	0	98.93	363324	NC_042756.1

Lactuca serriola	18039	3.16E+05	77%	0	98.93	363328	NC_042378.1
Lactuca serriola	18039	3.16E+05	77%	0	98.93	363328	MK820672.1
Lactuca sativa	18039	3.16E+05	77%	0	98.93	363324	MK642355.1
Lactuca sativa var. capitata	18039	3.16E+05	77%	0	98.93	363324	MZ159953.1
Lactuca virosa	18033	3.16E+05	77%	0	98.92	373019	MZ159961.1
Lactuca saligna	18022	3.15E+05	77%	0	98.9	368269	NC_042406.1
Lactuca saligna	18022	3.15E+05	77%	0	98.9	368269	MK759657.1
Lactuca saligna	18022	3.15E+05	77%	0	98.9	368269	MZ159956.1
Saussurea costus	16223	2.91E+05	76%	0	98.88	320439	NC_059793.1
Arctium lappa	16183	3.09E+05	76%	0	98.78	312598	NC_058644.1
Arctium tomentosum	16183	3.09E+05	76%	0	98.78	312609	NC_058643.1
Bidens tripartita	13708	2.44E+05	62%	0	98.21	216786	NC_062671.1
Bidens tripartita	13708	2.44E+05	62%	0	98.21	216786	MW838188.1
Bidens biternata	13494	2.19E+05	64%	0	98.25	198476	NC_062672.1
Bidens bipinnata	13494	2.19E+05	64%	0	98.25	198476	MW838193.1
Bidens biternata	13494	2.19E+05	64%	0	98.25	198476	MW838191.1
Bidens biternata	13494	2.19E+05	64%	0	98.25	198476	MW838190.1

Bidens bipinnata	13494	2.19E+05	64%	0	98.25	198476	NC_060635.1
Bidens alba var. radiata	13485	2.21E+05	64%	0	98.22	213288	MW838194.1
Helianthus annuus	12994	2.34E+05	67%	0	97.96	300947	MN171345.1
Helianthus annuus	12994	2.36E+05	68%	0	97.98	295586	MH704580.1
Helianthus annuus	12994	2.34E+05	67%	0	97.96	305217	MG735191.1
Helianthus annuus	12994	2.34E+05	67%	0	97.96	300945	KF815390.1
Helianthus grosseserratus	12994	2.25E+05	64%	0	97.96	273543	NC_051989.1
Helianthus annuus	12990	2.43E+05	68%	0	97.94	316582	MG770607.2
Helianthus occidentalis	12990	2.34E+05	67%	0	97.94	281175	NC_058584.1
Helianthus strumosus	12979	2.42E+05	66%	0	97.92	281056	NC_051990.1
Helianthus tuberosus	12975	2.34E+05	67%	0	97.93	281287	NC_058585.1
Helianthus annuus	12971	2.40E+05	69%	0	97.9	306018	MN175741.1
Bidens parviflora	12901	2.22E+05	60%	0	98.64	195825	NC_062670.1
Bidens pilosa	12883	2.06E+05	60%	0	98.59	183061	NC_062673.1
Bidens pilosa	12883	2.06E+05	60%	0	98.59	183061	MW838192.1
Heuchera parviflora var. saurensis	12139	1.59E+05	51%	0	93.29	542954	KR559021.1
Ageratum conyzoides	10255	1.94E+05	60%	0	97.98	219198	NC_053927.1

Saussurea diamantiaca	9790	16626	5%	0	98.75	173114	NC_062692.1
Aphyllon epigalium subsp. *epigalium*	9371	31556	11%	0	98.47	147128	MH129026.1
Diospyros oleifera	7336	1.44E+05	49%	0	93.8	493958	MW970112.1
Lonicera japonica	7275	1.69E+05	52%	0	93.5	884352	MZ504724.1
Triosteum pinnatifidum	7033	1.55E+05	50%	0	92.65	803609	NC_064333.1
Triosteum pinnatifidum	7033	1.55E+05	50%	0	92.65	803609	MW526076.1
Juglans mandshurica	6556	35188	11%	0	95.26	161386	MZ900993.1
Panax vietnamensis	6497	96047	28%	0	95.63	317650	MZ826164.1
Panax quinquefolius	6497	82361	24%	0	95.63	270043	MZ826160.1
Panax ginseng	6475	1.40E+05	43%	0	95.55	464661	MZ389476.1
Panax ginseng	6469	1.40E+05	43%	0	95.52	464705	MW029460.1
Actinidia eriantha	6453	1.44E+05	47%	0	93.83	768883	MZ959063.1
Panax notoginseng	6429	31657	10%	0	95.36	159699	MZ826159.1
Panax notoginseng	6429	76844	17%	0	95.36	332082	MZ826157.1
Panax notoginseng	6429	1.85E+05	43%	0	95.36	792375	MZ826156.1
Actinidia chinensis	6421	1.30E+05	44%	0	93.72	718940	MZ959061.1
Betula pendula	6407	1.43E+05	47%	0	94.64	581505	LT855379.1

Bombax ceiba	6336	1.45E+05	45%	0	94.93	594390	NC_038052.1
Bombax ceiba	6336	1.45E+05	45%	0	94.93	594390	MG788014.1
Ilex pubescens	6300	1.56E+05	52%	0	95.34	517520	NC_045078.1
Ilex pubescens	6300	1.56E+05	52%	0	95.34	517520	MK714017.1
Bupleurum chinense	6298	1.57E+05	43%	0	94.71	435023	OK166971.1
Gossypium trilobum	6283	1.52E+05	45%	0	94.63	644460	KR736346.1
Gossypium thurberi	6283	1.52E+05	45%	0	94.63	644395	KR736343.1
Gossypium arboreum	6274	1.62E+05	45%	0	94.59	687482	KR736342.1
Gossypium barbadense	6274	1.64E+05	45%	0	94.59	677434	KP898249.1
Xanthoceras sorbifolium	6272	1.54E+05	44%	0	94.61	575633	MK333231.1
Gossypium raimondii	6272	1.52E+05	45%	0	94.58	643914	KR736345.1
Gossypium davidsonii	6272	1.52E+05	45%	0	94.58	644311	KR736344.1
Gossypium raimondii	6272	1.57E+05	45%	0	94.58	676078	KU317325.1
Hibiscus cannabinus	6268	1.32E+05	45%	0	94.58	570031	MK961310.1
Gossypium hirsutum	6268	1.47E+05	45%	0	94.58	621884	JX065074.1
Gossypium harknessii	6266	1.53E+05	45%	0	94.56	668464	JX536494.1
Gossypium harknessii	6266	1.50E+05	45%	0	94.56	666081	JX944506.1

Gossypium hirsutum	6266	1.53E+05	45%	0	94.56	668584	JX944505.1
Hibiscus cannabinus	6259	1.33E+05	45%	0	94.53	569915	MF163174.1
Abelmoschus esculentus	6244	86965	28%	0	94.5	355223	OL348387.1
Toona ciliata	6224	1.29E+05	44%	0	94.35	683000	OM574630.1
Acer truncatum	6207	1.44E+05	45%	0	94.36	791052	MZ318049.1
Acer miaotaiense	6196	1.39E+05	45%	0	94.3	819227	MZ636518.1
Spondias mombin	6180	1.22E+05	43%	0	94.24	674158	NC_045035.1
Spondias mombin	6180	1.22E+05	43%	0	94.24	674158	MN057920.1
Spondias tuberosa	6174	1.38E+05	47%	0	94.22	779106	NC_045036.1
Carica papaya	6170	1.37E+05	47%	0	94.23	476890	EU431224.1
Tribulus terrestris	6165	1.53E+05	46%	0	94.22	415781	MK431825.1
Toona sinensis	6156	1.27E+05	44%	0	94.1	638482	OM574631.1
Cotinus coggygria	6143	1.34E+05	46%	0	94.14	564322	MZ089853.1
Ricinus communis	6143	1.36E+05	46%	0	93.59	502773	HQ874649.1
Helianthus annuus	6133	6238	1%	0	99.47	3373	MF828620.1
Helianthus annuus	6133	6238	1%	0	99.47	3373	MF828619.1
Sapindus mukorossi	6109	1.45E+05	45%	0	94	602121	NC_050850.1

Sapindus mukorossi	6109	1.45E+05	45%	0	94	602121	MT806100.1

Supplementary Figures:

(A)

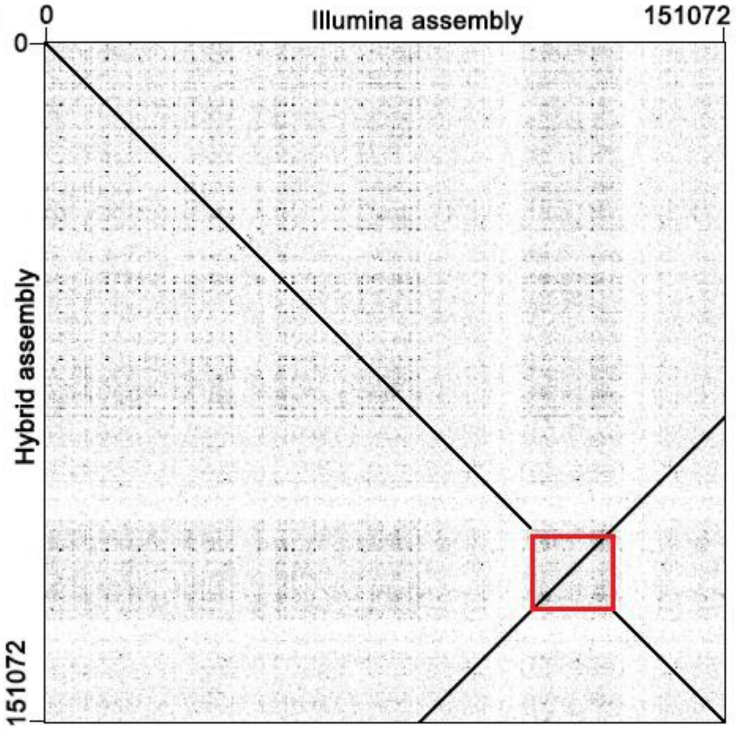

(B)

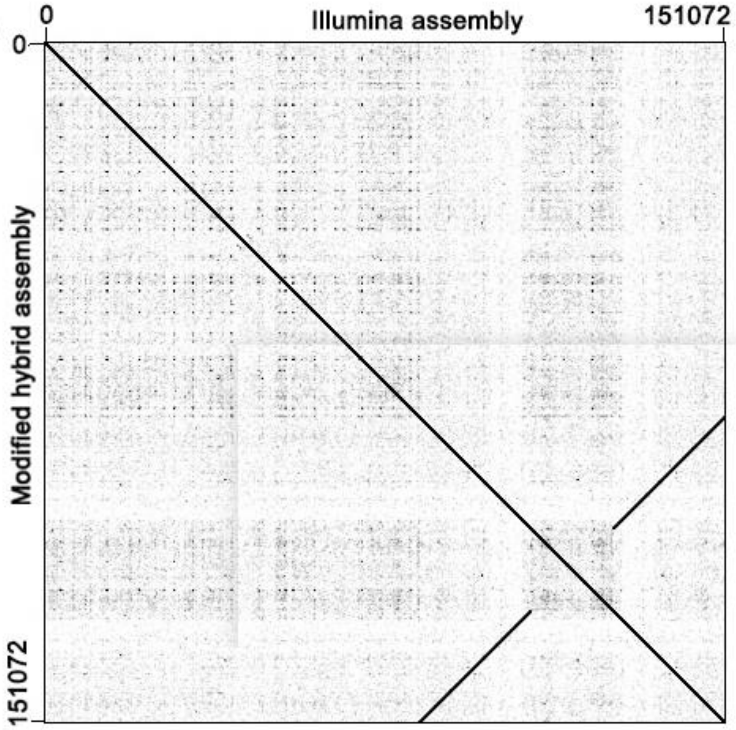

Figure S1. Dotplot of *A. giraldii* plastome assembled by Illumina data and hybrid of Illumina and Nanopore data (A). Dotplot of *A. giraldii* plastome assembled by Illumina data and modified hybrid of Illumina and Nanopore data (B). In (A), the horizontal direction is the *A. giraldii* plastome assembled by Illumina data. The vertical direction is the *A. giraldii* plastome assembled by hybrid data of Illumina and Nanopore. The red box indicates that the hybrid assembled plastome has its small single-copy (SSC) region inverted compared to the plastome assembled with Illumina data. In (B), the horizontal direction is the *A. giraldii* plastome assembled by Illumina data. The vertical direction is the SSC region modification of the hybrid assembled plastome.

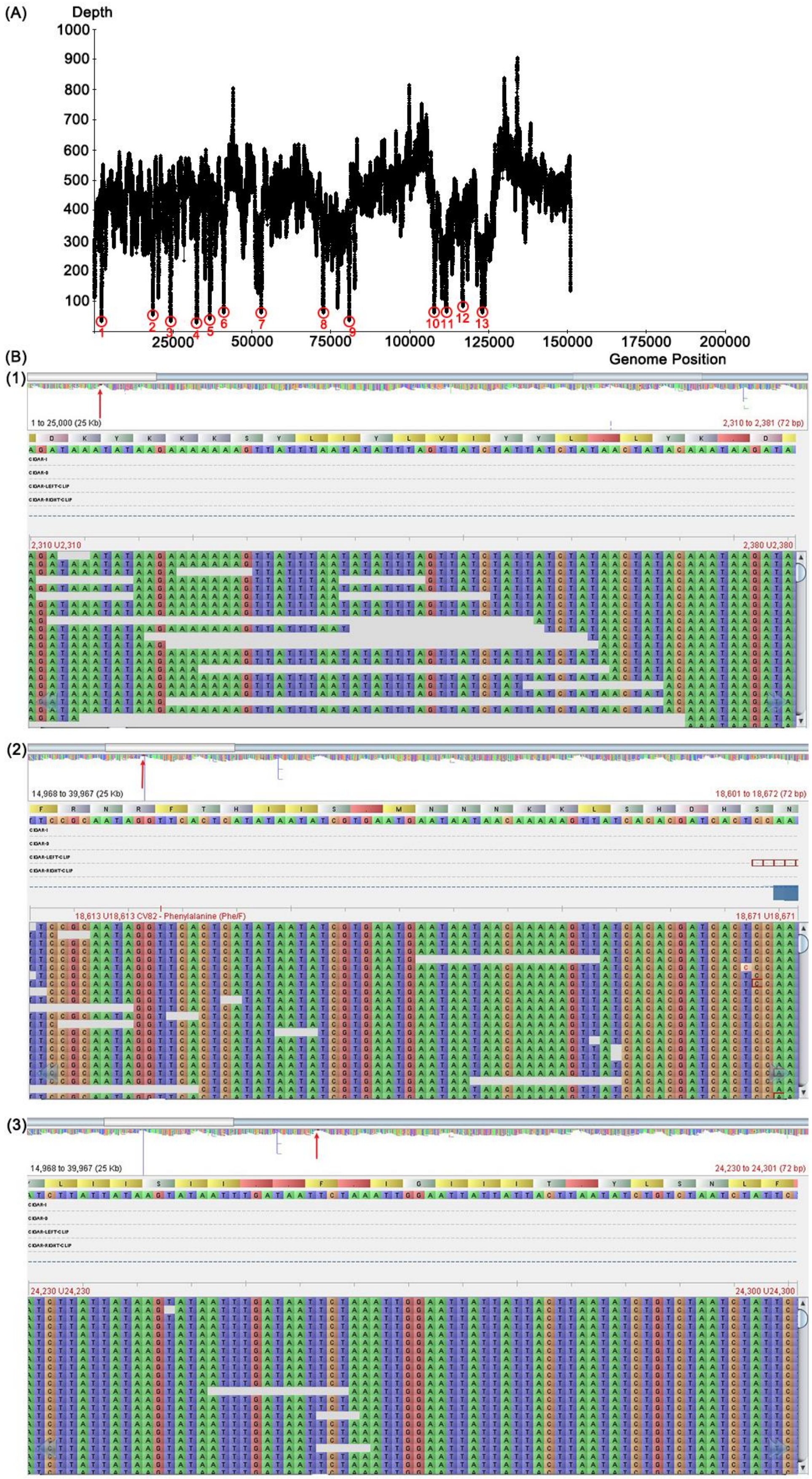
(A)
Depth
1000
900
800
700
600
500
400
300
200
100
1
2
3
4
5
6
7
8
9
10
11
12
13
25000
50000
75000
100000
125000
150000
175000
200000
Genome Position
(B)
(1)
1 to 25,000 (25 Kb)
2,310 to 2,381 (72 bp)
2,310 U2,310
2,380 U2,380
(2)
14,968 to 39,967 (25 Kb)
18,601 to 18,672 (72 bp)
18,613 U18,613 CV82 - Phenylalanine (Phe/F)
18,671 U18,671
(3)
14,968 to 39,967 (25 Kb)
24,230 to 24,301 (72 bp)
24,230 U24,230
24,300 U24,300

(4)

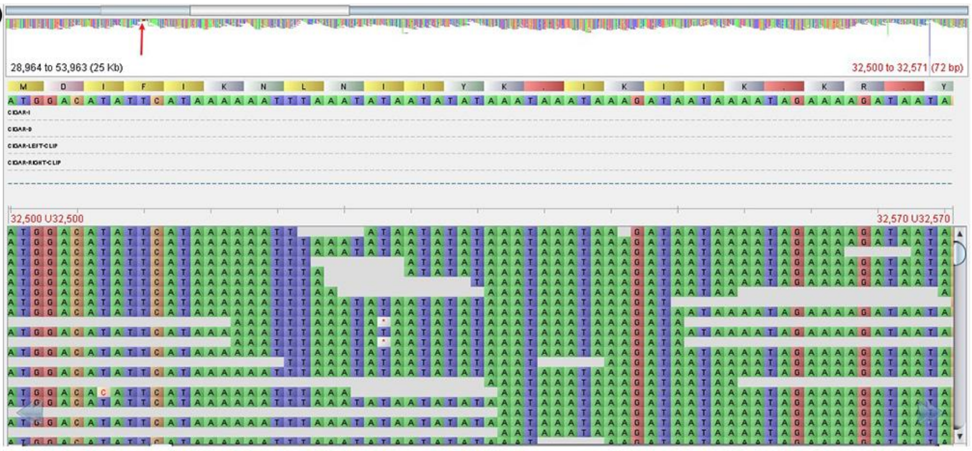
28,964 to 53,963 (25 Kb)
32,500 to 32,571 (72 bp)
32,500 U32,500
32,570 U32,570

(5)

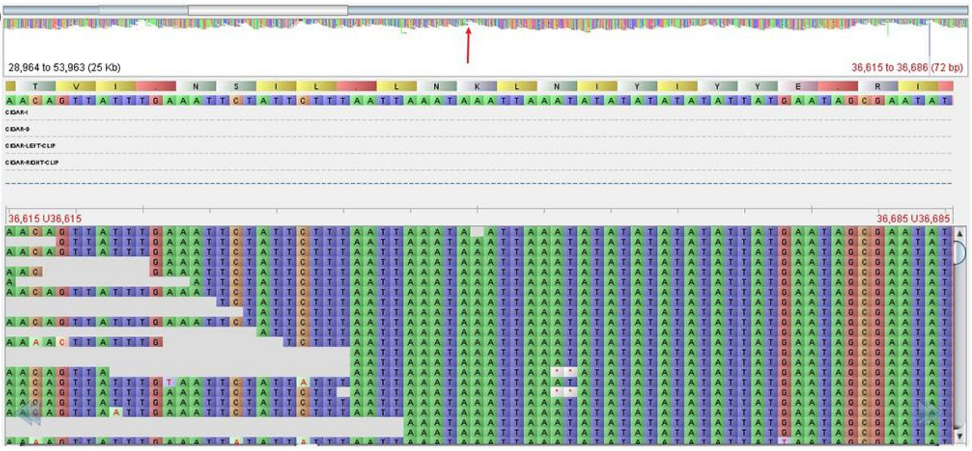
28,964 to 53,963 (25 Kb)
36,615 to 36,686 (72 bp)
36,615 U36,615
36,685 U36,685

(6)

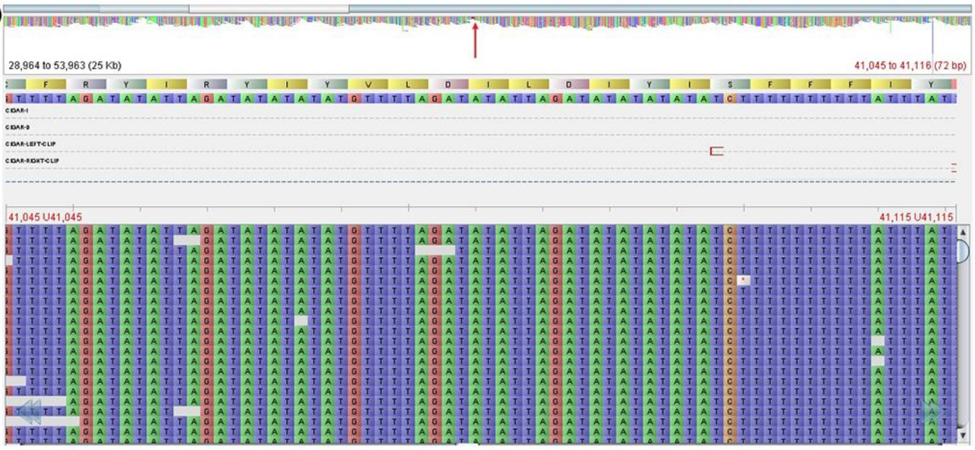
28,964 to 53,963 (25 Kb)
41,045 to 41,116 (72 bp)
41,045 U41,045
41,115 U41,115

(7)

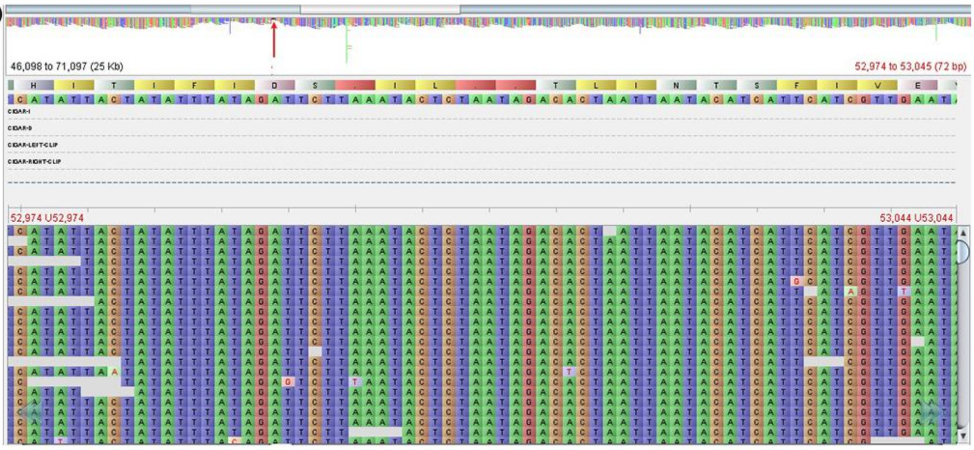
46,098 to 71,097 (25 Kb)
52,974 to 53,045 (72 bp)
52,974 U52,974
53,044 U53,044

(8)
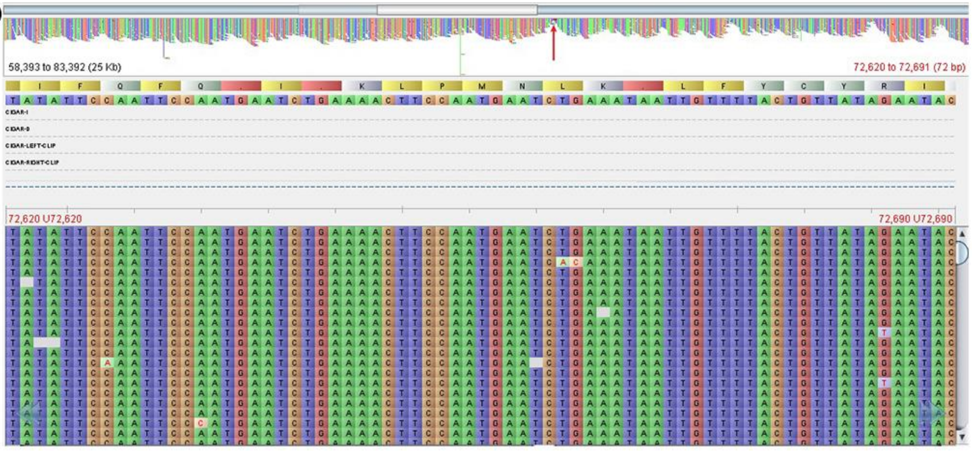
58,393 to 83,392 (25 Kb)
72,620 to 72,691 (72 bp)
72,620 U72,620
72,690 U72,690
(9)
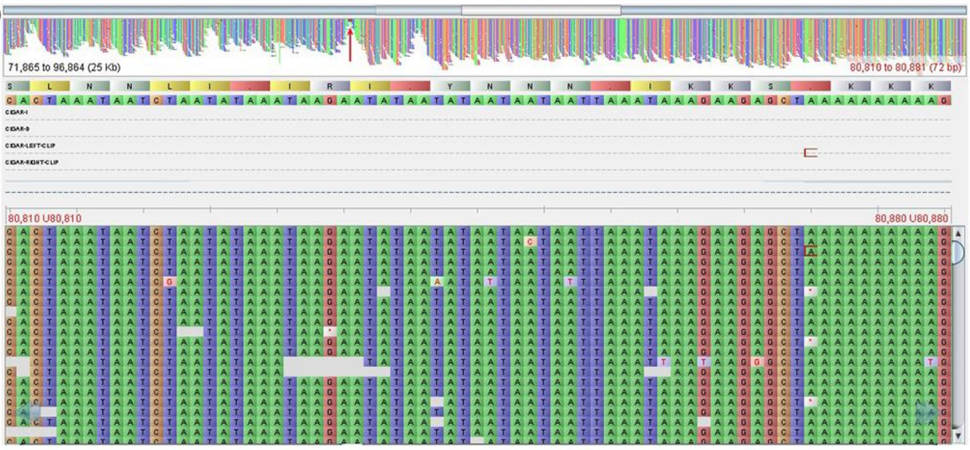
71,865 to 96,864 (25 Kb)
80,810 to 80,881 (72 bp)
80,810 U80,810
80,880 U80,880
(10)
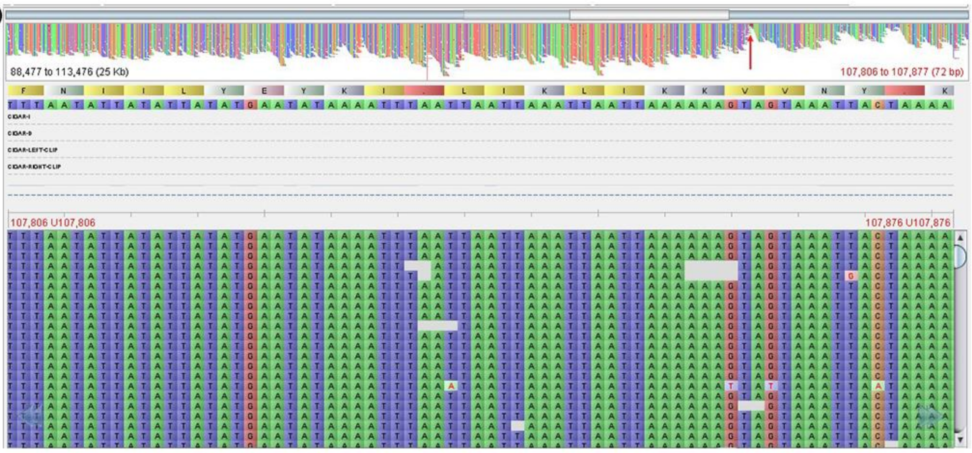
88,477 to 113,476 (25 Kb)
107,806 to 107,877 (72 bp)
107,806 U107,806
107,876 U107,876
(11)
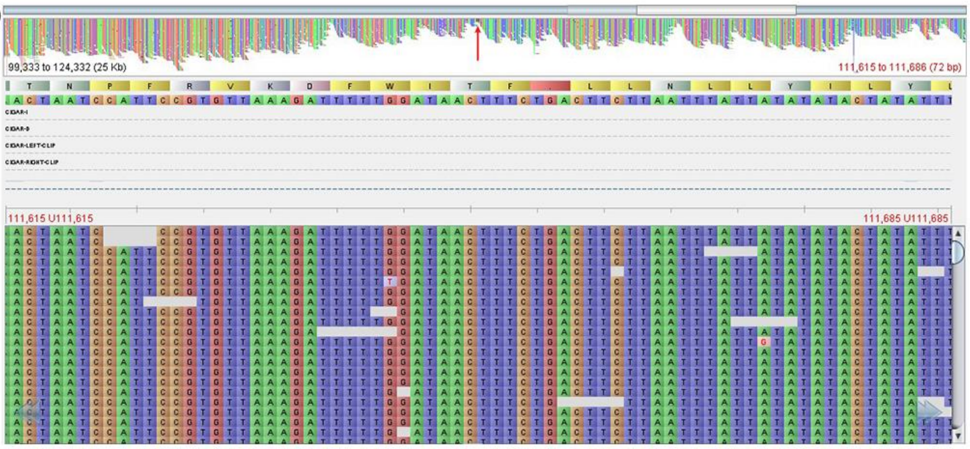
99,333 to 124,332 (25 Kb)
111,615 to 111,686 (72 bp)
111,615 U111,615
111,685 U111,685

(12)

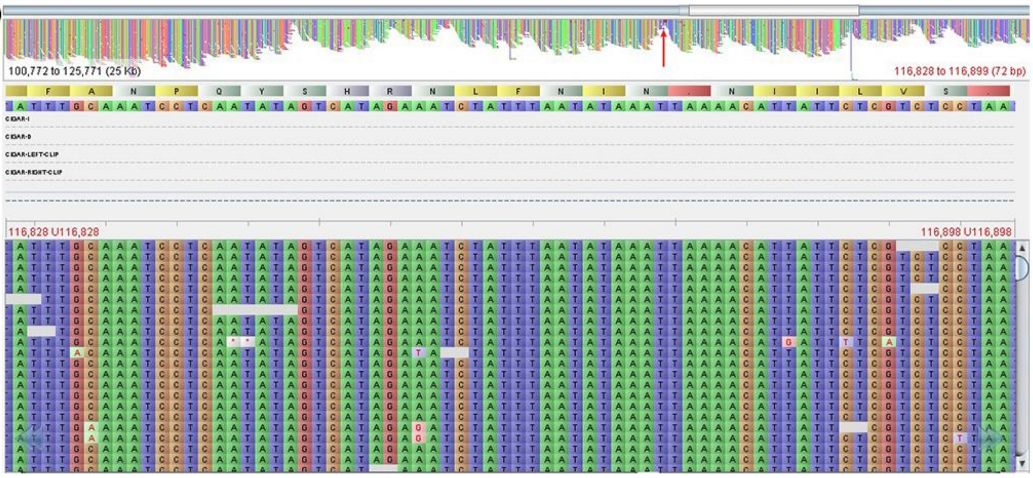

(13)

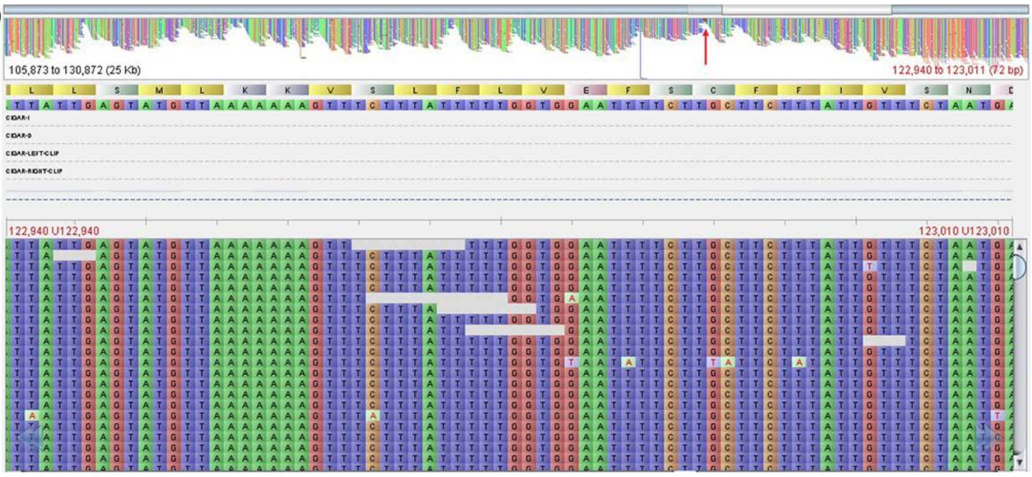

Figure S2. The coverage depth of the *A. giraldii* plastome (A) and the alignments of reads to the assembled genome for regions (1-13) having low coverage depth, which is marked with a red circle in figure A (B). In (A), the horizontal axis represents the genome positions. The vertical axis represents the coverage depth. (B) In each panel, the top of each image refers to a certain area of reads coverage and the position pointed by the red arrow on each image is the position with low coverage depth. The (A) was drawn by minimap2 software and the (B) was drawn by Tablet software.

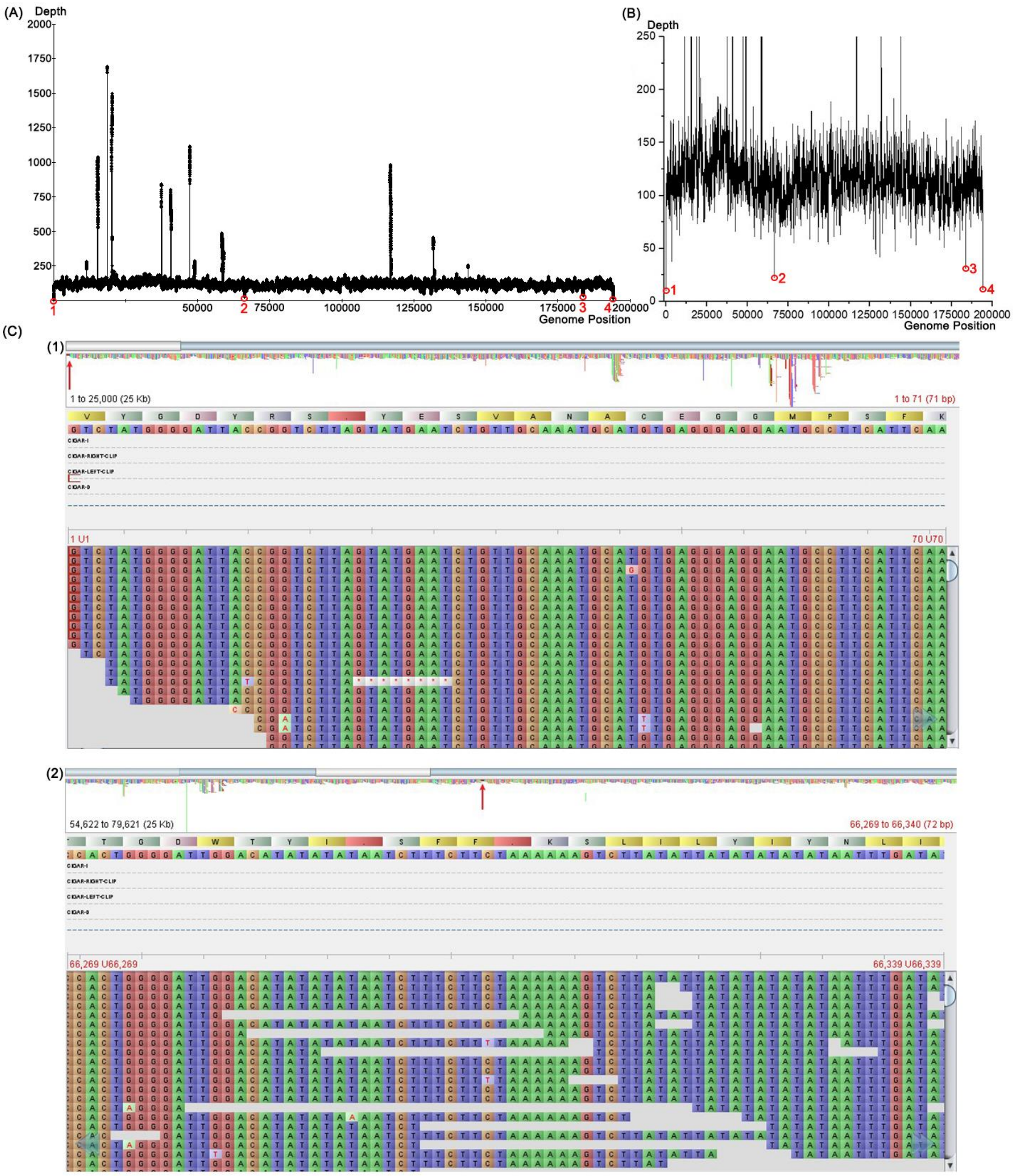
(A)
Depth
Genome Position
(B)
Depth
Genome Position
(C)
(1)
1 to 25,000 (25 Kb)
1 to 71 (71 bp)
(2)
54,622 to 79,621 (25 Kb)
66,269 to 66,340 (72 bp)

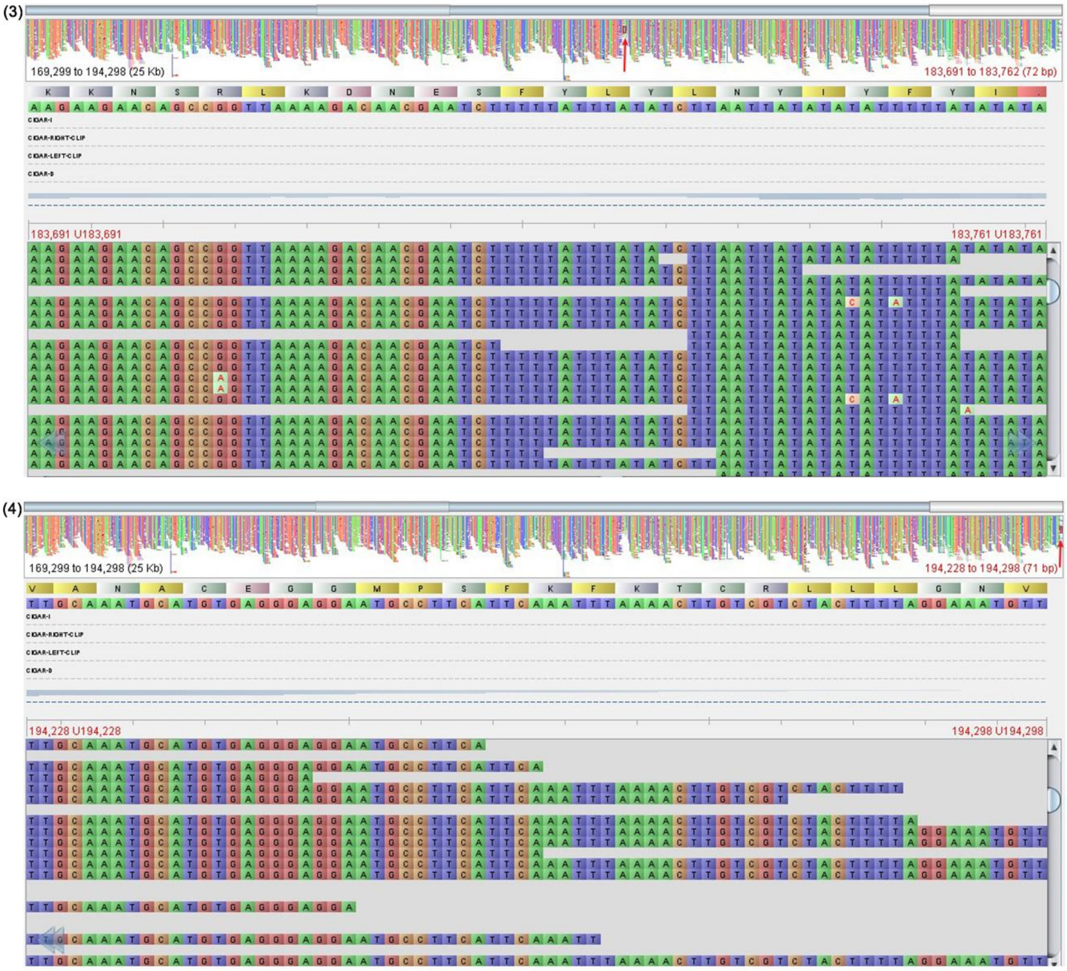

Figure S3. The coverage depth of the *A. giraldii* mitogenome (A), the detailed coverage depth with the maximum y-axis value is 250 (B), and the alignment of reads to the assembled mitogenome for the regions (1-4) having a low coverage depth (C). In (A) and (B), the horizontal axis represents the genome positions. The vertical axis represents the coverage depth. The high spikes in figure (A) represent one or more reads covering this position have base sequencing errors. In (C), the top of each image refers to a certain area of reads coverage and the position pointed by the red arrow on each image is the position with low coverage depth. The (A) and (B) were drawn by minimap2 software and the (C) were drawn by Tablet software.

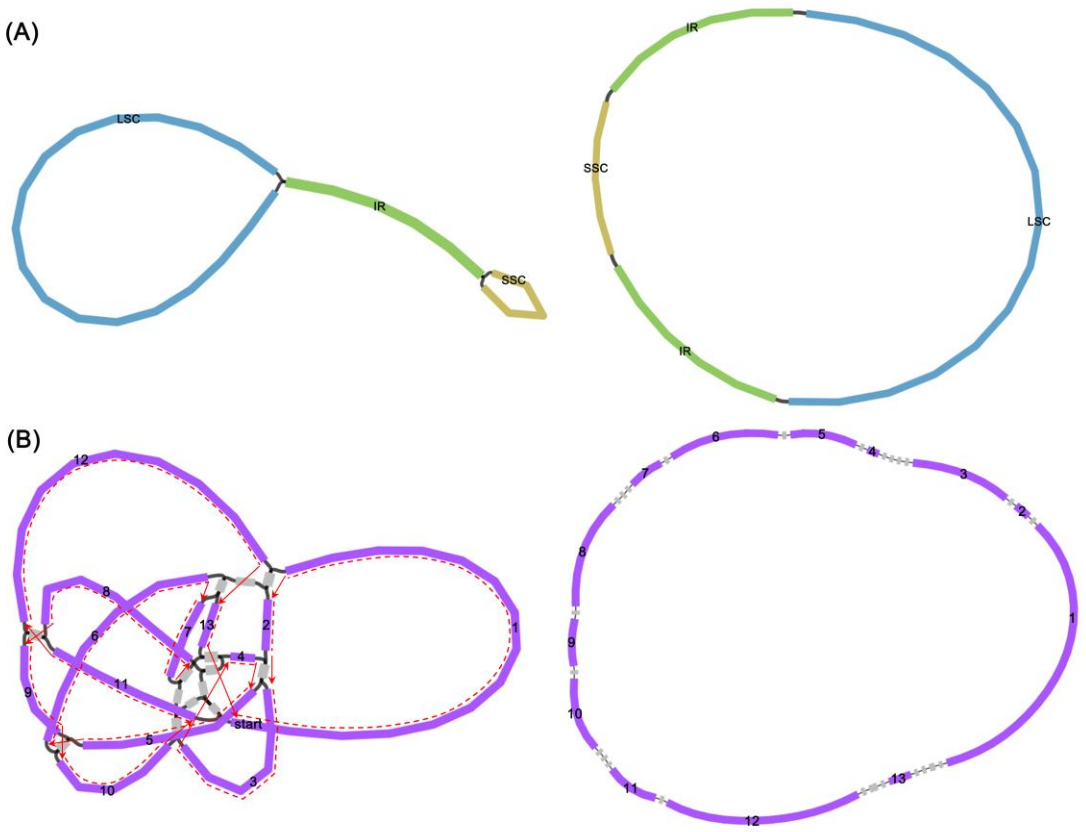

Figure S4. Untig graphs of *A. giraldii* plastome (A) and mitogenome assembly (B). In (A) the circular and linear structures from the left to the right represent the LSC, IR, and SSC regions, respectively in the plastome. In (B), each edge represents a contig. The number on each edge represents the order of contig connection. The arrow between the two contigs represents the direction of the contig connection. The gray fragment represents the repeated sequence between two contigs. This unit graph represents the branched polymeric structure of the mitogenome. The circle on the right is the principal chromosome that is joined together by contigs shown on the left side. These untig graphs of *A. giraldii* plastome (A) and mitogenome assembly (B) were drawn by Bandage software.

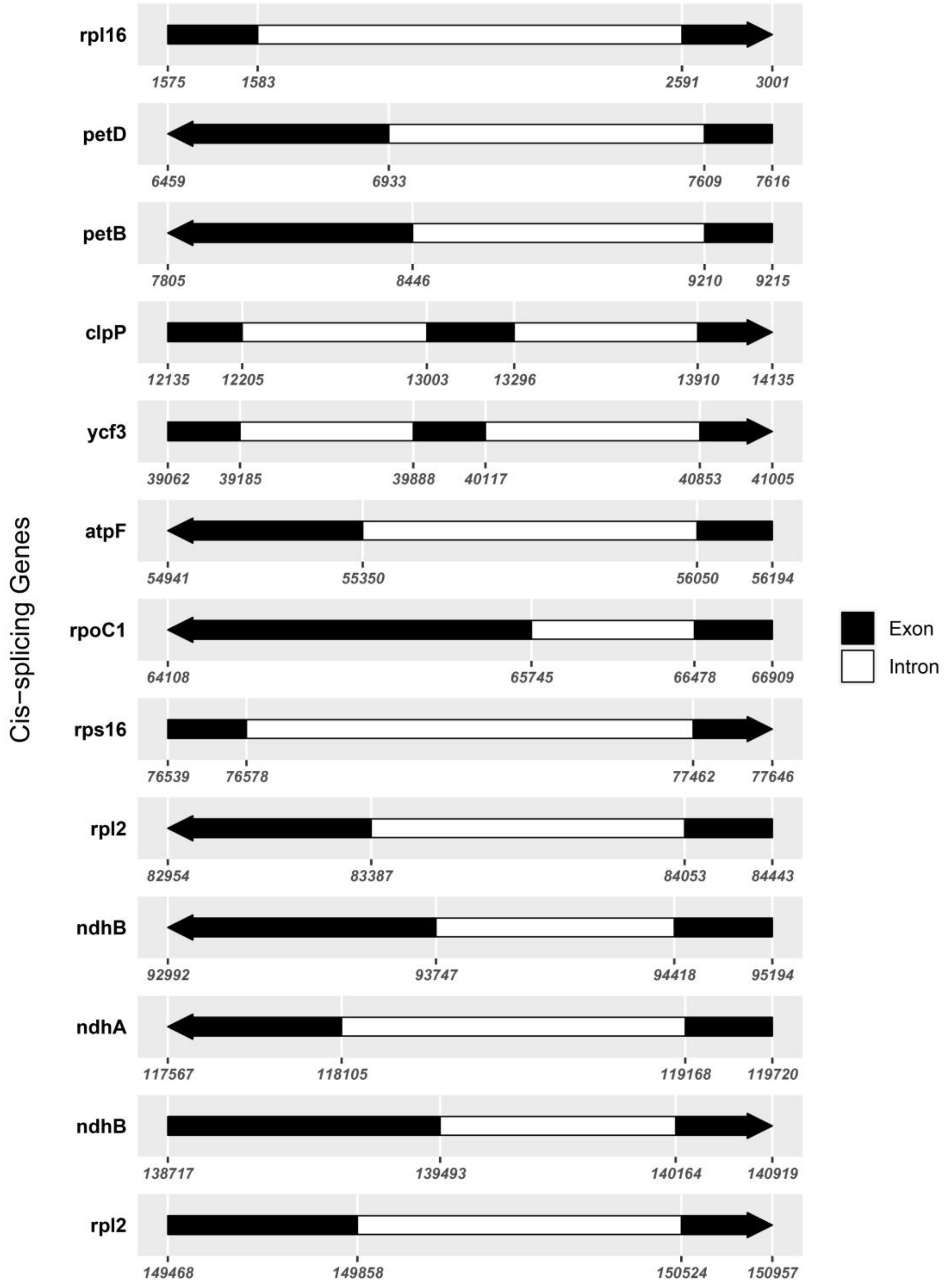

Figure S5. The cis-splicing genes of the *A. giraldii* plastome. The graph was created using CPGview-RSG (http://www.1kmpg.cn/cpgviewer_cn/). The column on the left is the name of the gene. The black and white blocks represent the exon and intron, respectively. The numbers below the arrow of each gene represent the start and end sites of the exons and introns. This graph was created using CPGview-RSG (http://www.herbalgenomics.org/cpgview)**.**

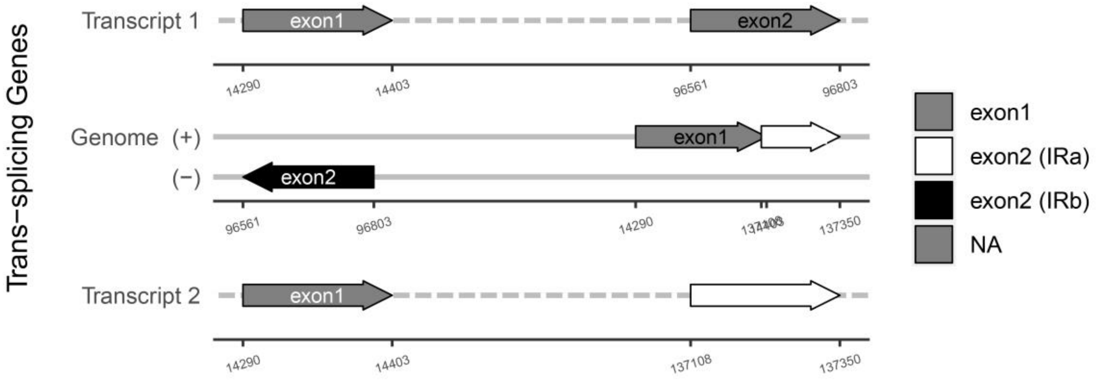

Figure S6. The map of the trans-splicing gene *rps*12 in the *A. giraldii* plastome. The graph was created using CPGview-RSG (http://www.1kmpg.cn/cpgviewer_cn/). The black, grey, and white colors represent exons from different regions, as shown on the right. Transcripts 1 and 2 represent the products of two copies of the *rps12* gene. The numbers below the arrow of each exon represent the start and end sites. This graph was created using CPGview-RSG (http://www.herbalgenomics.org/cpgview)**.**

(A)

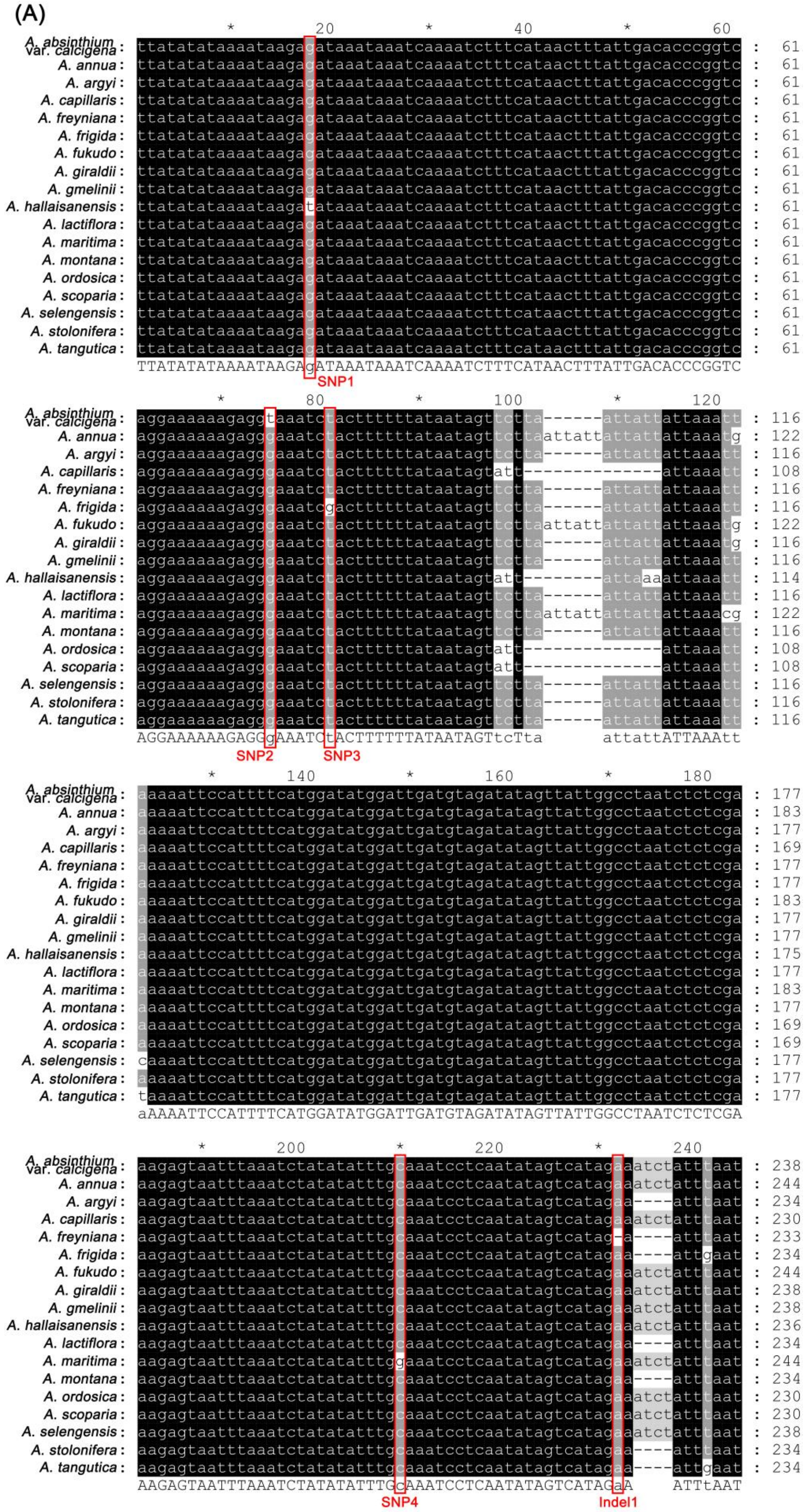
A. absinthium var. calcigena
A. annua
A. argyi
A. capillaris
A. freyniana
A. frigida
A. fukudo
A. giraldii
A. gmelinii
A. hallaisanensis
A. lactiflora
A. maritima
A. montana
A. ordosica
A. scoparia
A. selengensis
A. stolonifera
A. tangutica
SNP1
SNP2
SNP3
SNP4
Indel1

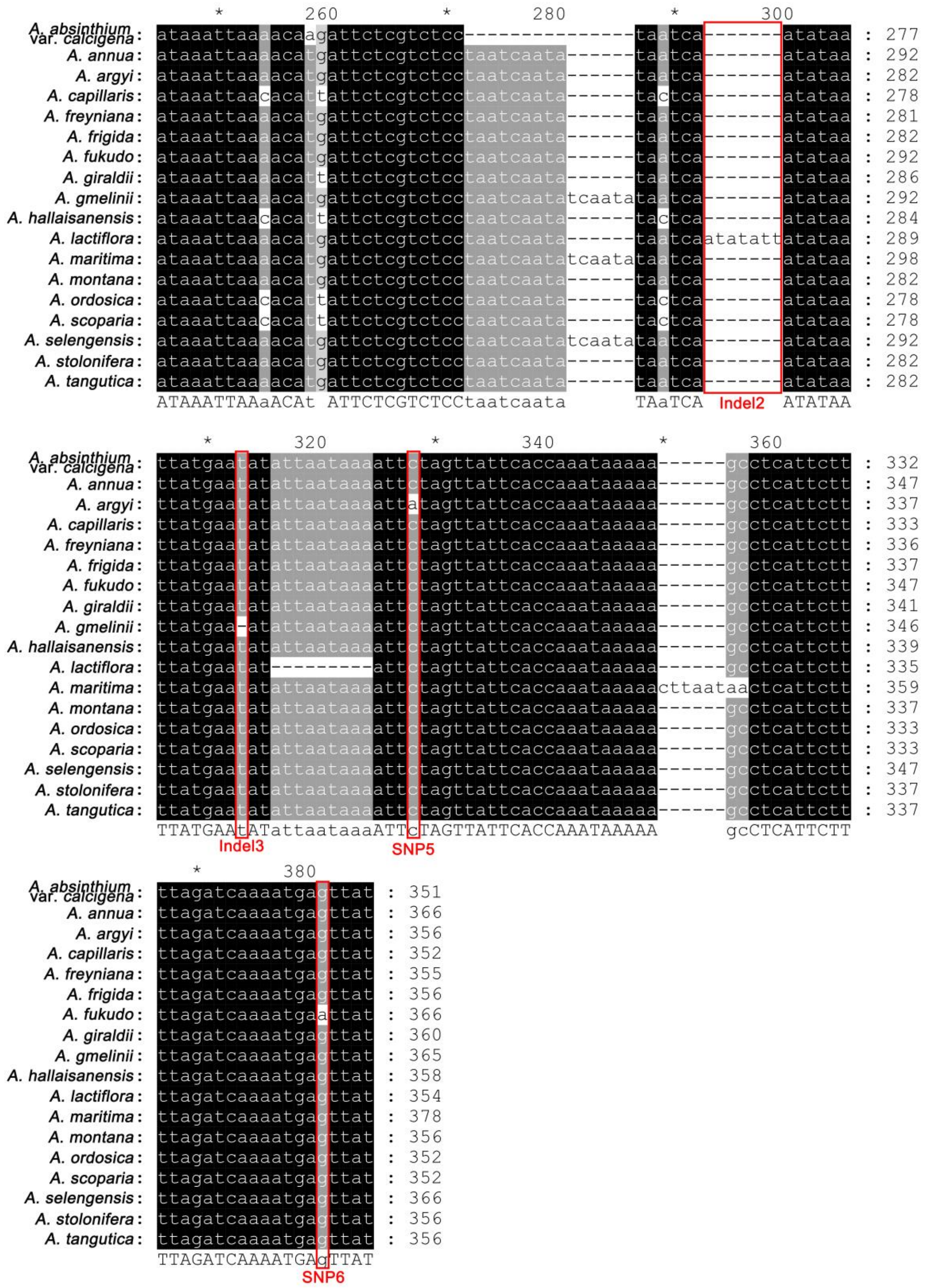
A. absinthium var. calcigena
A. annua
A. argyi
A. capillaris
A. freyniana
A. frigida
A. fukudo
A. giraldii
A. gmelinii
A. hallaisanensis
A. lactiflora
A. maritima
A. montana
A. ordosica
A. scoparia
A. selengensis
A. stolonifera
A. tangutica
Indel2
Indel3
SNP5
SNP6

(B)

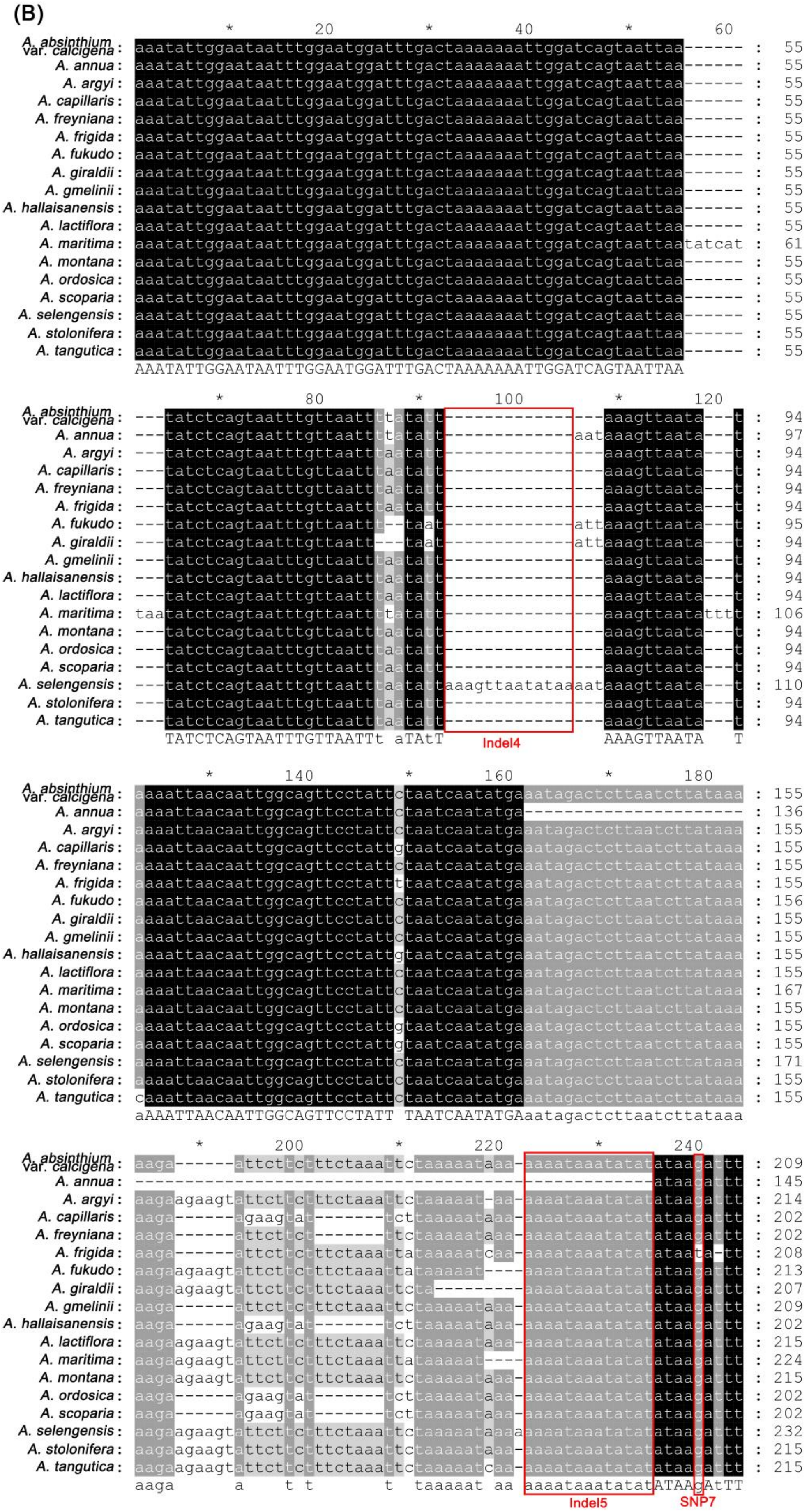
A. absinthium var. calcigena
A. annua
A. argyi
A. capillaris
A. freyniana
A. frigida
A. fukudo
A. giraldii
A. gmelinii
A. hallaisanensis
A. lactiflora
A. maritima
A. montana
A. ordosica
A. scoparia
A. selengensis
A. stolonifera
A. tangutica
Indel4
Indel5
SNP7

```
                              *       260         *       280         *       300
A. absinthium var. calcigena : tttatttgatttttttagtatattttcattcagattttggtggtggggagtcttcttttcc : 270
A. annua          : tttatttgaattttttagtatattttcattcagattttggtggtggggagtcttcttttcc : 206
A. argyi          : tttatttgatttttttagtatattttcattcagattttggtggtggggagtcttcttttcc : 275
A. capillaris     : tttatttgatttttttagtatattttcattcagattttggtggtggggagtcttcttttcc : 263
A. freyniana      : tttatttgatttttttagtatattttcattcagattttggtggtggggagtcttcttttcc : 263
A. frigida        : tttatttgatttttttagtatattttcattcagattttggtggtggggagtcttcttttcc : 269
A. fukudo         : tttatttgaattttttagtatattttcattcagattttggtggtggggagtcttcttttcc : 274
A. giraldii       : tttatttg-attttttagtatattttcattcagattttggtggtggggagtcttcttttcc : 267
A. gmelinii       : tttatttgatttttttagtatattttcattcagattttggtggtggggagtcttcttttcc : 270
A. hallaisanensis : tttatttgatttttttagtagattttcattcagattttggtggtggggagtcttcttttcc : 263
A. lactiflora     : tttatttgatttttttagtatattttcattcagattttggtggtggggagtcttcttttcc : 276
A. maritima       : tttatttgaattttttagtatattttcattcagattttggtggtggggagtcttcttttcc : 285
A. montana        : tttatttgatttttttagtatattttcattcagattttggtggtggggagtcttcttttcc : 276
A. ordosica       : tttatttgatttttttagtatattttcattcagattttggtggtggggagtcttcttttcc : 263
A. scoparia       : tttatttgatttttttagtatattttcattcagattttggtggtggggagtcttcttttcc : 263
A. selengensis    : tttatttgatttttttagtatattttcattcagattttggtggtggggagtattcttttcc : 293
A. stolonifera    : tttatttgatttttttagtatattttcattcagattttggtggtggggagtcttcttttcc : 276
A. tangutica      : tttatttgatttttttagtatattttcattcagattttggtggtggggagtcttcttttcc : 276
                    TTTATTTGatTTTTTTAGTAtATTTTCATTCAGATTTTGGTGGTGGGGAGTcTTCTTTTCC

                        *       320         *       340         *       360
A. absinthium var. calcigena : ccatcgaccttaaaagaataaataatgaaaaaatttatatttatcaggaagggcagata : 331
A. annua          : ccatcgacctttaaaagaataaataatgaaaaaatttatatttatcaggaagggcagata : 267
A. argyi          : ccatcgactttaaaagaataaataatgaaaaaatttatatttatcaggaagggcagata : 336
A. capillaris     : ccatcgaccttaaaagaataaataatgcaaaaatttatatttatcaggaagggcagata : 324
A. freyniana      : ccatcgaccttaaaagaataaataatgaaaaaatttatatttatcaggaagggcagata : 324
A. frigida        : ccatcgaccttaaaagaataaataatgaaaaaatttatatttatcaggaagggcagata : 330
A. fukudo         : ccatcgaccttaaaagaataaataatgaaaaaatttatatttatcaggaagggcagata : 335
A. giraldii       : ccatcgaccttaaaagaataaataatgaaaaaatttatatttatcaggaagggcagata : 328
A. gmelinii       : ccatcgaccttaaaagaataaataatgaaaaaatttatatttatcaggaagggcagata : 331
A. hallaisanensis : ccatcgaccttaaaagaataaataatgaaaaaatttatatttatcaggaagggcagata : 324
A. lactiflora     : ccatcgactttaaaagaataaataatgaaaaaatttatatttatcaggaagggcagata : 337
A. maritima       : ccatcgaccttaaaagaataaataatgaaaaaatttatatttatcaggaagggcatata : 346
A. montana        : ccatcgactttaaaagaataaataatgaaaaaatttatatttatcaggaagggcagata : 337
A. ordosica       : ccatcgaccttaaaagaataaataatgaaaaaatttatatttatcaggaagggcagata : 324
A. scoparia       : ccatcgaccttaaaagaataaataatgaaaaaatttatatttatcaggaagggcagata : 324
A. selengensis    : ccatcgaccttaaaagaataaataatgaaaaaatttgatatttatcaggaagggcagata : 354
A. stolonifera    : ccatcgactttaaaagaataaataatgaaaaaatttatatttatcaggaagggcagata : 337
A. tangutica      : ccatcgactttaaaagaataaataatgaaaaaatttatatttatcaggaagggcagata : 337
                    CCATCGAC TTTAAAAGAATAAATAATGaAAAAATTTtATATTTATCAGGAAGGGCAgATA
                                               SNP8

                        *       380         *       400         *       420
A. absinthium var. calcigena : gaatattttttagttcaaattaattaattgttcaaactaatccattccgtgttaaagatttt : 392
A. annua          : gaatattttttagttcaaattaattaattgttcaaactaatccattccgtgttaaagatttt : 328
A. argyi          : gaatattttttagttcaaattaattaattgttcaaactaatccattccgtgttaaagatttt : 397
A. capillaris     : gaatattttttagttcaaattaattaattgttcaaactaatccattccgtgttaaagatttt : 385
A. freyniana      : gaatattttttagttcaaattaattaattgttcaaactaatccattccgtgttaaagatttt : 385
A. frigida        : gaatattttttagttcaaattaattaattgttcaaactaatccattccgtgttaaagatttt : 391
A. fukudo         : gaatattttttagttcaaattaattaattgttcaaactaatccattccgtgttaaagatttt : 396
A. giraldii       : gaatattttttagttcaaattaattaattgttcaaactaatccattccgtgttaaagatttt : 389
A. gmelinii       : gaatattttttagttcaaattaattaattgttcaaactaatccattccgtgttaaagatttt : 392
A. hallaisanensis : gaatattttttagttcaaattaattaattgttcaaactaatccattccgtgttaaagatttt : 385
A. lactiflora     : gaatattttttagttcaaattaattaattgttcaaactaatccattccgtgttaaagatttt : 398
A. maritima       : gaatattttttagttcaaattaattaattgttcaaactaatccattccgtgttaaagatttt : 407
A. montana        : gaatattttttagttcaaattaattaattgttcaaactaatccattccgtgttaaagatttt : 398
A. ordosica       : gaatattttttagttcaaattaattaattgttcaaactaatccattccgtgttaaagatttt : 385
A. scoparia       : gaatattttttagttcaaattaattaattgttcaaactaatccattccgtgttaaagatttt : 385
A. selengensis    : gaatattttttagttcaaattaattaattgttcaaactaatacattccgtgttaaagatttt : 415
A. stolonifera    : gaatattttttagttcaaattaattaattgttcaaactaatccattccgtgttaaagatttt : 398
A. tangutica      : gaatattttttagttcaaattaattaattgttcaaactaatccattccgtgttaaagatttt : 398
                    GAATATTTTTAGTTCAAATTAATTAATTGTTCAAACTAATcCATTCCGTGTTAAAGATTTT

                        *       440         *       460         *       480
A. absinthium var. calcigena : tggataattttctgacttcttcatttatt--atatactatatttaatgtattttccatata : 451
A. annua          : tggataactttctgacttcttaatttattatatatactatatttaatgtattttccatata : 389
A. argyi          : tggataactttctgacttcttaatttattatatatactatatttaatttattttccatata : 458
A. capillaris     : tggataactttctgacttcttaattgattatatatactatatttaatgtattttccatatc : 446
A. freyniana      : tggataactttctgacttcttaatttatt--atatactatatttaatgtattttcaatata : 444
A. frigida        : tggataactttctgacttcttaatttatt--atatactatatttaatgtattttccatatc : 450
A. fukudo         : tggataactttctgacttcttaatttattatatatactatatttaatgtattttccatata : 457
A. giraldii       : tggataactttctgacttcttaatttattatatatactatatttaatgtattttccatata : 450
A. gmelinii       : tggataactttctgacttcttaatttatt--atatactatatttaatgtattttcaatata : 451
A. hallaisanensis : tggataactttctgacttcttaatttattagatatactatatttaatgtattttccatatc : 446
A. lactiflora     : tggataactttctgacttcttaatttattatatatactatatttaatttattttccatata : 459
A. maritima       : tggataactttctgacttcttaattcattatatatactatatttaatgtattttccatata : 468
A. montana        : tggataactttctgacttcttaatttattatatatactatatttaatttatttgccatata : 459
A. ordosica       : tggataactttctgacttcttaatttattatatatactatatttaatgtattttccatatc : 446
A. scoparia       : tggataactttctgacttcttaatttattagatatactatatttaatgtattttacatatc : 446
A. selengensis    : tggataactttctgacttcttaatttattatatatactatattaaatgtattttccatata : 476
A. stolonifera    : tggataactttctgacttcttaatttattatatatactatatttcatttattttccatata : 459
A. tangutica      : tggataactttctgacttcttaatttattatatatactatatttaattttttttccatata : 459
                    TGGATAAcTTTCTGACTTCTTaATTtATTa ATATACTATATTTtaAT TaTTTtccATAT
                                                               SNP9  SNP10 SNP11
```

```
                             *      500         *      520          *      540
A. absinthium var. calcigena : tatccaattttcggcccaatgaaaaatcaatgaataatc-aataaaagaacttaattgttg : 511
A. annua                     : tatccaattttcagcc-----------caatgaataatc-aataaaagaacttaattgttg : 438
A. argyi                     : tatccaattttcagcc-----------caatgaaaaatccaataaaagaacttaattgttg : 508
A. capillaris                : tatccaattttcagcccaatgaataatcaatgaataatc-aataaaagaacttaattgttg : 506
A. freyniana                 : tatccaattttcagcccaatgaaaaatcaatgaataatc-aataaaagaacttaattgttg : 504
A. frigida                   : tatccaattttcagcccaatgaaaaatcaatgaataatc-aataaaagaacttaattgttg : 510
A. fukudo                    : tatccaattttcagcc-----------caatgaataatc-aataaaagaacttaattgttg : 506
A. giraldii                  : tatccaattttcagcccaatgaataatcaatgaataatc-aataaaagaacttaattgttg : 510
A. gmelinii                  : tatccaattttcagcccaatgaaaaatcaatgaataatc-aataaaagaacttaattgttg : 511
A. hallaisanensis            : tatccaattttcagcccaatgaataatcaatgaataatc-aataaaagaacttaattgttg : 506
A. lactiflora                : tatccaattttcagcc-----------caatgaaaaatccaataaaagaacttaattgttg : 509
A. maritima                  : tatccaattttcagcc-----------caatgaataatc-aataaaagaacttaattgttg : 517
A. montana                   : tatccaattttcagcc-----------caatgaaaaatctaataaaagaacttaattgttg : 509
A. ordosica                  : tatccaattttcagcccaatgaataatcaatgaataatc-aataaaagaacttaattgttg : 506
A. scoparia                  : tatccaattttcagcccaatgaataatcaatgaataatc-aataaaagaacttaattgttg : 506
A. selengensis               : tatccaattttcagcccaatgaataatcaatgaataatc-aataaaagaacttaattgttg : 536
A. stolonifera               : tatccaattttcagcc-----------caatgaaaaatccaataaaagaacttaattgttg : 509
A. tangutica                 : tatccaattttcagcc-----------taatgaaaaatccaataaaagaacttaattgttg : 509
                               TATCCAATTTTCaGCC           cAATGAA AATC AATAAAAGAACTTAATTGTTG

                             *      560         *      580          *      600         *
A. absinthium var. calcigena : agatattattatatgtacaagtggcaatttggagtaatccaaaatagacatattttagatt : 572
A. annua                     : agatattattatatgtacaagtggcaatttggagtaatccaaaatagacatattttagatt : 499
A. argyi                     : agatattattatatgtgcaagtggcaatttggagtaatccaaaatagacatattttagatt : 569
A. capillaris                : agatattattatatgtacaagtggcaatttggagtaatccaaaatagacatattttagatt : 567
A. freyniana                 : agatattattatatgtacaagtggcaatttggagtaatccaaaatagacatattttagatt : 565
A. frigida                   : agatattattatatgtacaagtggcaatttggagt-------aatagacatattttagatt : 564
A. fukudo                    : agatattattatatgtacaagtggcaatttggagtaatccaaaatagacatattttagatt : 567
A. giraldii                  : agatattattatatgtacaagtggcaatttggagtaatccaaaaaagacatattttagatt : 571
A. gmelinii                  : agatattattatatgtacaagtggcaatttggagtaatccaaaatagacatattttagatt : 572
A. hallaisanensis            : agatattattatatgtacaagtggcaatttggagtaatccaaaatagacatattttagatt : 567
A. lactiflora                : agatattattatatgtacaagtggcaatttggagtaatccaaaatagacatattttagatt : 570
A. maritima                  : agatattattatatgtacaagtggcaatttggagtaatacaaaatagacatattttagatt : 578
A. montana                   : agatattattatatgtacaagtggcaatttggagtaatccaaaatagacatattttagatt : 570
A. ordosica                  : agatattattatatgtacaagtggcaatttggagtaatccaaaatagacatattttagatt : 567
A. scoparia                  : agatattattatatgtacaagtggcaatttggagtaatccaaaatagacatattttagatt : 567
A. selengensis               : agatattattatatgtacaagtggcaattttgagtaatccaaaatagacatattttagatt : 597
A. stolonifera               : agatattattatatgtacaagtggcaatttggagtaatccaaaatagacatattttagatt : 570
A. tangutica                 : agatattattatatgtacaagtggcaatttggagtaatccaaaatagacatattttagatt : 570
                               AGATATTATTATATGTaCAAGTGGCAATTTgGAGTaatccaaAAtAGACATATTTTAGATT

                                    620         *      640          *      660         *
A. absinthium var. calcigena : cattgataaaatttagtttgtgtttcaaattgaatttattaaatcagataaaccagaaata : 633
A. annua                     : cattgataaaatttagtttgtgtttcaaattgaatttattaaatcagataaaccagaaata : 560
A. argyi                     : cattgataaaatttagtttgtgtttcaaattgaatttattaaatcagataaaccagaaata : 630
A. capillaris                : cattgataaaatttagtttgtgtttcaaattgaatttattaaatcagataaaccagaaata : 628
A. freyniana                 : cattgataaaatttagtttgtgtttcaaattgaatttattaaatcagataaaccagaaata : 626
A. frigida                   : cattgataaaattgagtttgtgtttcaaattgaatttattaaatcagataaaccagaaata : 625
A. fukudo                    : cattgataaaatttagtttgtgtttcaaattgaatttattaaatcaataaaccagaaata : 628
A. giraldii                  : cattgataaaatttagtttgtgtttcaaattgaatttattaaatcagataaaccagaaata : 632
A. gmelinii                  : cattgataaaatttagtttgtgtttcaaattgaatttattaaatcagataaaccagaaata : 633
A. hallaisanensis            : cattgataaaatttagtttgtgtttcaaattgaatttattaaatcagataaaccagaaata : 628
A. lactiflora                : cattgataaaatttagtttgtgtttcaaattgaatttattaaatcagataaaccagaaata : 631
A. maritima                  : cattgataaaattgagtttgtgtttcaaattgaatttattaaataagataaaccagaaata : 639
A. montana                   : cattgataaaatttagtttgtgtttcaaattgaatttgttaaatcagataaaccagaaata : 631
A. ordosica                  : cattgataaaatttagtttgtgtttcaaattgaatttattaaatcagataaaccagaaata : 628
A. scoparia                  : cattgataaaatttagtttgtgtttcaaattgaatttattaaatcagataaaccagaaata : 628
A. selengensis               : cattgataaaatttagtttgtgtttcaaattgaatttattaaatcagataaaccagaaata : 658
A. stolonifera               : cattgataaaatttagtttgtgtttcaaattgaatttattaaatcagataaaccagaaata : 631
A. tangutica                 : cattgataaaatttagtttgtgtttcaaattgaatttattaaatcagataaaccagaaata : 631
                               CATTGATAAAATTtAGTTTGTGTTTCAAATTGAATTTaTTAAATcAgATAAACCAGAAATA

                                      680         *      700          *      720         *
A. absinthium var. calcigena : atgacaataaaagaaaaaagtttttagtctatcgaagaattctatcgttgcaagagtcgt : 694
A. annua                     : atgacaataaaagaaaaaagtttttagtctatcgaagaattctatcgttgcaagagtcgt : 621
A. argyi                     : atgacaataaaagaaaaaagtttttgagtctatcgaagaattctatcgttgcaagagtcgt : 691
A. capillaris                : atgacaat-aaa-aaaaaagtttttagtctatcgaagaattctatcgttgcaagagtcgt : 687
A. freyniana                 : atgacaataaaagaaaaaagtttttagtctatcgaagaattctatcgttgcaagagtcgt : 687
A. frigida                   : atgacaataaaagaaaaaagtttttagtctatcgaagaattctatcgttgcaagagtcgt : 686
A. fukudo                    : atgacaataaaagaaaaaagtttttagtctatcgaagaattctatcgttacaagagtcgt : 689
A. giraldii                  : atgacaataaaagaaaaaagtttttagtctatcgaagaattctatcgttgcaagagtcgt : 693
A. gmelinii                  : atgacaataaaagaaaaaagtttttagtctatcgaagaattctatcgttgcaagagtcgt : 694
A. hallaisanensis            : atgacaataaaa-aaaaaagtttttagtctatcgaagaattctatcgttgcaagagtcgt : 688
A. lactiflora                : atgacaataaaagaaaaaagtttttgagtctatcgaagaattctatcgttgcaagagtcgt : 692
A. maritima                  : atgacaataaaagaaaaaagtttttagtctatcgaagaattctatcgttgcaagagtcgt : 700
A. montana                   : atgacaataaaagaaaaaagtttttgagtctatcgaagaattctatcgttgcaagagtcgt : 692
A. ordosica                  : atgacaat-aaa-aaaaaagtttttagtctatcgaagaattctatcgttgcaagagtcgt : 687
A. scoparia                  : atgacaat-aaa-aaaaaagtttttagtctatcgaagaattctatcgttgcaagagtcgt : 687
A. selengensis               : atgacaataaaagaaaaaagtttttgagtctatcgaagaattctatcgttgcaagagtcgt : 719
A. stolonifera               : atgacaataaaagaaaaaagtttttgagtctatcgaagaattctatcgttgcaagagtcgt : 692
A. tangutica                 : atgacaataaaagaaaaaagtttttgagtctatcgaagaattctatcgttgcaagagtcgt : 692
                               ATGACAATaAAAgAAAAAAGTTTTT AGTCTATCGAAGAATTCTATCGTTgCAAGAGTCGT
```

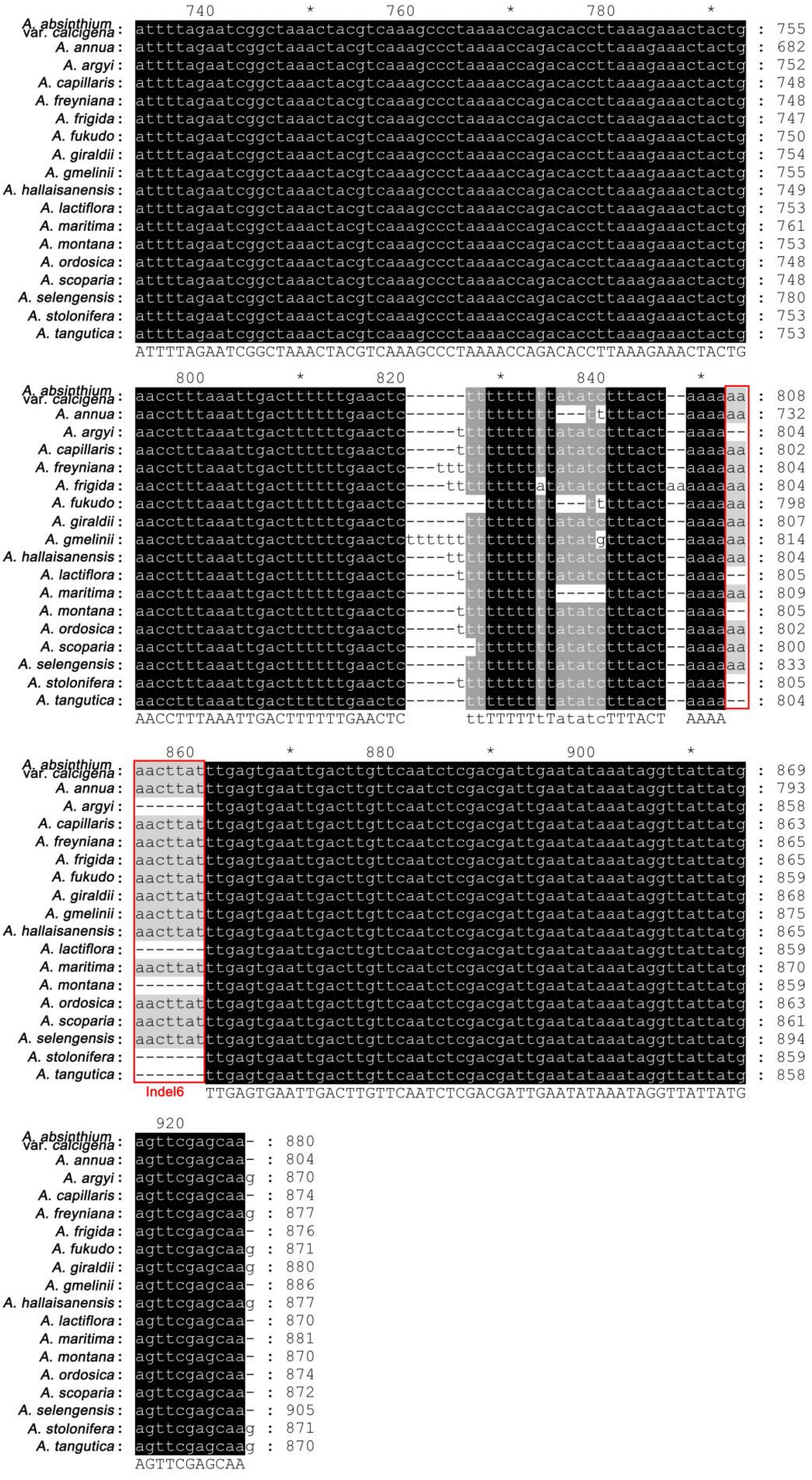

740 * 760 * 780 *
A. absinthium var. calcigena : attttagaatcggctaaactacgtcaaagccctaaaaccagacaccttaaagaaactactg : 755
A. annua : attttagaatcggctaaactacgtcaaagccctaaaaccagacaccttaaagaaactactg : 682
A. argyi : attttagaatcggctaaactacgtcaaagccctaaaaccagacaccttaaagaaactactg : 752
A. capillaris : attttagaatcggctaaactacgtcaaagccctaaaaccagacaccttaaagaaactactg : 748
A. freyniana : attttagaatcggctaaactacgtcaaagccctaaaaccagacaccttaaagaaactactg : 748
A. frigida : attttagaatcggctaaactacgtcaaagccctaaaaccagacaccttaaagaaactactg : 747
A. fukudo : attttagaatcggctaaactacgtcaaagccctaaaaccagacaccttaaagaaactactg : 750
A. giraldii : attttagaatcggctaaactacgtcaaagccctaaaaccagacaccttaaagaaactactg : 754
A. gmelinii : attttagaatcggctaaactacgtcaaagccctaaaaccagacaccttaaagaaactactg : 755
A. hallaisanensis : attttagaatcggctaaactacgtcaaagccctaaaaccagacaccttaaagaaactactg : 749
A. lactiflora : attttagaatcggctaaactacgtcaaagccctaaaaccagacaccttaaagaaactactg : 753
A. maritima : attttagaatcggctaaactacgtcaaagccctaaaaccagacaccttaaagaaactactg : 761
A. montana : attttagaatcggctaaactacgtcaaagccctaaaaccagacaccttaaagaaactactg : 753
A. ordosica : attttagaatcggctaaactacgtcaaagccctaaaaccagacaccttaaagaaactactg : 748
A. scoparia : attttagaatcggctaaactacgtcaaagccctaaaaccagacaccttaaagaaactactg : 748
A. selengensis : attttagaatcggctaaactacgtcaaagccctaaaaccagacaccttaaagaaactactg : 780
A. stolonifera : attttagaatcggctaaactacgtcaaagccctaaaaccagacaccttaaagaaactactg : 753
A. tangutica : attttagaatcggctaaactacgtcaaagccctaaaaccagacaccttaaagaaactactg : 753
ATTTTAGAATCGGCTAAACTACGTCAAAGCCCTAAAACCAGACACCTTAAAGAAACTACTG
800 * 820 * 840 *
A. absinthium var. calcigena : aacctttaaattgactttttgaactc------ttttttttatatctttact--aaaaaa : 808
A. annua : aacctttaaattgactttttgaactc------tttttttt---ttttact--aaaaaa : 732
A. argyi : aacctttaaattgactttttgaactc-----tttttttttatatctttact--aaaa-- : 804
A. capillaris : aacctttaaattgactttttgaactc-----tttttttttatatctttact--aaaaaa : 802
A. freyniana : aacctttaaattgactttttgaactc---tttttttttttatatctttact--aaaaaa : 804
A. frigida : aacctttaaattgactttttgaactc----tttttttttatatatctttactaaaaaaaa : 804
A. fukudo : aacctttaaattgactttttgaactc--------ttttttt---ttttact--aaaaaa : 798
A. giraldii : aacctttaaattgactttttgaactc------ttttttttatatctttact--aaaaaa : 807
A. gmelinii : aacctttaaattgactttttgaactcttttttttttttttatatgtttact--aaaaaa : 814
A. hallaisanensis : aacctttaaattgactttttgaactc----ttttttttttatatctttact--aaaaaa : 804
A. lactiflora : aacctttaaattgactttttgaactc-----tttttttttatatctttact--aaaa-- : 805
A. maritima : aacctttaaattgactttttgaactc------tttttttt-----tttact--aaaaaa : 809
A. montana : aacctttaaattgactttttgaactc-----tttttttttatatctttact--aaaa-- : 805
A. ordosica : aacctttaaattgactttttgaactc-----tttttttttatatctttact--aaaaaa : 802
A. scoparia : aacctttaaattgactttttgaactc-------tttttttatatctttact--aaaaaa : 800
A. selengensis : aacctttaaattgactttttgaactc------ttttttttatatctttact--aaaaaa : 833
A. stolonifera : aacctttaaattgactttttgaactc-----tttttttttatatctttact--aaaa-- : 805
A. tangutica : aacctttaaattgactttttgaactc------ttttttttatatctttact--aaaa-- : 804
AACCTTTAAATTGACTTTTTGAACTC ttTTTTTtTatatcTTTACT AAAA
860 * 880 * 900 *
A. absinthium var. calcigena : aacttatttgagtgaattgacttgttcaatctcgacgattgaatataaataggttattatg : 869
A. annua : aacttatttgagtgaattgacttgttcaatctcgacgattgaatataaataggttattatg : 793
A. argyi : -------ttgagtgaattgacttgttcaatctcgacgattgaatataaataggttattatg : 858
A. capillaris : aacttatttgagtgaattgacttgttcaatctcgacgattgaatataaataggttattatg : 863
A. freyniana : aacttatttgagtgaattgacttgttcaatctcgacgattgaatataaataggttattatg : 865
A. frigida : aacttatttgagtgaattgacttgttcaatctcgacgattgaatataaataggttattatg : 865
A. fukudo : aacttatttgagtgaattgacttgttcaatctcgacgattgaatataaataggttattatg : 859
A. giraldii : aacttatttgagtgaattgacttgttcaatctcgacgattgaatataaataggttattatg : 868
A. gmelinii : aacttatttgagtgaattgacttgttcaatctcgacgattgaatataaataggttattatg : 875
A. hallaisanensis : aacttatttgagtgaattgacttgttcaatctcgacgattgaatataaataggttattatg : 865
A. lactiflora : -------ttgagtgaattgacttgttcaatctcgacgattgaatataaataggttattatg : 859
A. maritima : aacttatttgagtgaattgacttgttcaatctcgacgattgaatataaataggttattatg : 870
A. montana : -------ttgagtgaattgacttgttcaatctcgacgattgaatataaataggttattatg : 859
A. ordosica : aacttatttgagtgaattgacttgttcaatctcgacgattgaatataaataggttattatg : 863
A. scoparia : aacttatttgagtgaattgacttgttcaatctcgacgattgaatataaataggttattatg : 861
A. selengensis : aacttatttgagtgaattgacttgttcaatctcgacgattgaatataaataggttattatg : 894
A. stolonifera : -------ttgagtgaattgacttgttcaatctcgacgattgaatataaataggttattatg : 859
A. tangutica : -------ttgagtgaattgacttgttcaatctcgacgattgaatataaataggttattatg : 858
Indel6
TTGAGTGAATTGACTTGTTCAATCTCGACGATTGAATATAAATAGGTTATTATG
920
A. absinthium var. calcigena : agttcgagcaa- : 880
A. annua : agttcgagcaa- : 804
A. argyi : agttcgagcaag : 870
A. capillaris : agttcgagcaa- : 874
A. freyniana : agttcgagcaag : 877
A. frigida : agttcgagcaa- : 876
A. fukudo : agttcgagcaag : 871
A. giraldii : agttcgagcaag : 880
A. gmelinii : agttcgagcaa- : 886
A. hallaisanensis : agttcgagcaag : 877
A. lactiflora : agttcgagcaa- : 870
A. maritima : agttcgagcaa- : 881
A. montana : agttcgagcaa- : 870
A. ordosica : agttcgagcaa- : 874
A. scoparia : agttcgagcaa- : 872
A. selengensis : agttcgagcaa- : 905
A. stolonifera : agttcgagcaag : 871
A. tangutica : agttcgagcaag : 870
AGTTCGAGCAA

Figure S7. The alignment of two hypervariable regions, *ndh*G-*ndh*I (A), and *rpl*32-*trn*L-UAG (B) in 18 *A. giraldii* plastomes. A total of 11 SNPs (SNP1-11) and six Indel (Indel 1-6) from these two hypervariable regions can distinguish these 18 *A. giraldii* species completely. The red box represents the specific sites.
